# Supplementary material for: Reticulate allopolyploidy and subsequent dysploidy drive evolution and diversification in the cotton family
Source: Nat Commun. 2025 Aug 12;16:7480. doi: 10.1038/s41467-025-62644-7 (PMC12343791; doi:10.1038/s41467-025-62644-7)
Supplement: Supplementary file 1 — Supplementary Information [file 41467_2025_62644_MOESM1_ESM.pdf]

Reticulate allopolyploidy and subsequent dysploidy drive evolution and diversification in the cotton family

Zhang *et al.*

Supplementary Notes

Supplementary Note 1: The paleopolyploid history of the Malvaceae remains unresolved or controversial

Based on genome synteny and synonymous substitution rates (*K*s), the cotton lineage was initially thought to have undergone an abrupt five- or six-fold ploidy increase (decaploidy or dodecaploidy), compared to the cacao or grape genomes, approximately 60 million years ago (Mya)[1]. Subsequent analyses, using various comparative, computational and statistical approaches, revealed a decaploid ancestor of the cotton genome[2]. Later, phylogenetic analysis of the durian genome suggested that durian and cotton shared a paleopolyploidization event[3]. However, re-analysis of the durian genome revealed that durian and cotton genomes were actually affected by two independent polyploidization events: hexaploidization in durian around 19 to 21 Mya and decaploidization in cotton around 13 to 14 Mya[4]. Meanwhile, analyses of transcriptomic and genomic data have proposed two possible hypotheses for complex evolutionary histories, each involving two independent autotetraploid events and one allopolyploid event[5]. One hypothesis suggests independent autopolyploidy events in Malvatheca and Sterculioideae/Tilioideae, with Helicteroideae being an allohexaploid. An alternative hypothesis proposed that Malvatheca, Sterculioideae and Tilioideae share an autotetraploidy and the members of Malvatheca (Bombacoideae + Malvoideae) are allodecaploid[5]. A more recent study revealed that the allohexaploid Helicteroideae are partially derived from the allotetraploid Sterculioideae and also form a component of the allodecaploid Malvatheca[6]. However, genomic data of the other three subfamilies (i.e., Brownlowioideae, Dombeyoideae and Tilioideae) were absent in this study, likely decreasing the power of the inference.

Supplementary Note 2: Nucleocytoplasmic conflict and gene tree discordance in the Malvaceae

We reconstructed phylogenetic trees using three genomes representing distinct genetic inheritances, i.e., nuclear, plastid and mitochondrial genomes. Analysis of the nuclear data revealed congruent topologies in both the coalescent and concatenation-based approaches (16,370 multi-copy and 1,904 single-copy genes, respectively), each being highly supported (100% bootstrap and 100% posterior probability values) (**Fig. 2A, Supplementary Fig. 10**). However, there were significant discrepancies between the nuclear phylogeny and those based on organelle-encoded genes (**Fig. 2A**). For example, within the Helicteroideae, *Du. zibethinus* and *R. pubescens* formed a monophyletic group in the plastid phylogeny, but displayed paraphyly in the mitochondrial phylogeny and polyphyly in the nuclear phylogeny, each with strong bootstrap supports (**Fig. 2A**). These results imply divergent evolutionary trajectories or rates of the three genomes.

Furthermore, we observed substantial discordance among the nuclear gene trees, with discrepancies ranging from 48 to 63% across all internal nodes of the nuclear phylogeny, except for the nodes representing the crown groups of Malvaceae, Malvadendrina and Malvatheca (**Supplementary Fig. 10**). Six of the seven internal nodes also exhibited a significant asymmetry (χ^2 test, $P < 0.001$) between two alternative topologies (i.e., q2 and q3) (**Supplementary Fig. 10**), indicating possible reticulation events beyond incomplete lineage sorting (ILS), such as ancient allopolyploidization and/or introgression.

Supplementary Note 3: Multiple rounds of species radiation and polyploidization within Malvaceae

Species radiation, characterized by rapid diversification, typically yields short branch lengths and discordance in the species tree, due to ILS and/or hybridization/introgression in the process. Here, short internal branch lengths (0.00030–0.0055 nucleotide substitutions per site or 0.038–0.74 coalescent units; **Supplementary Fig. 18**) and significant gene tree discordance (32–65% discordant gene trees; **Supplementary Fig. 21**) revealed multiple episodes of species radiation within the family. An initial radiation of ancient diploids led to the diversification of two Byttneriina subfamilies and the diploid ancestor of the Malvadendrina clade, followed by a second radiation that gave rise to at least five diploid progenitors (AA, BB, CC, DD, and EE) of the Malvadendrina paleopolyploids (**Supplementary Figs. 18** and **21**). This preceded the formation of the ancestral tetraploid genome (AABB, Mal-β event). Then radiation of the tetraploid resulted in six AABB ancestors of all AB-containing lineages (**Supplementary Figs. 18** and **21**). Later, the ancient decaploid originated (AABBCCDDEE, Mal-α) and the subsequent radiation resulted in the diversification into the three Malvatheca species (**Supplementary Figs. 18** and **21**).

Supplementary Note 4: Proposed taxonomic revision of Durionoideae subfam. nov.

Subgenome-aware phylogenetic and evolutionary analyses have also shown that *Du. zibethinus* and *R. pubescens* experienced different paleopolyploidy events during the early divergence of the Malvadendrina (**Figs. 2–4, Supplementary Figs. 18** and **19**). Whereas *R. pubescens* shares a close relationship with the C subgenome of *Du. zibethinus*, they do not form a sister group in the subgenome-aware phylogeny (**Fig. 4**). Instead, *R. pubescens* was identified as a sister group to the D subgenomes within the Malvatheca genomes (**Fig. 4, Supplementary Figs. 18** and **19**). The previous classification of *Du. zibethinus* and *R. pubescens* in the same subfamily was based on a plastome-based phylogenetic analysis[7,8]. Our results suggest that the maternal progenitor of *Du. zibethinus* likely contributed its C subgenome, whereas the maternal progenitor of the most recent common ancestor (MCRA) of the Malvatheca may be the AABB paleotetraploid (**Figs. 2A, 3** and **4**). This hypothesis could account for the observed sister relationship between *Du. zibethinus* and *R. pubescens*, as well as their non-sister relationship to the Malvatheca clade in the plastome-based phylogenetic tree (**Fig. 2A**). However, this hypothesis did not appear to be consistent with the mitochondrial phylogeny (**Fig. 2A**). Despite this discrepancy, our findings still suggest the assignment of *Du. zibethinus* and *R. pubescens* to different subfamilies. Consequently, we propose a novel subfamily, Durionoideae, to accommodate *Du. zibethinus* and its closely related taxa in the tribe Durioneae[9]. Conversely, while the subfamily Bombacoideae is recognized as paraphyletic, there is currently no compelling evidence for splitting or merging with the Malvoideae, considering the fact that they share a common paleodecaploid origin.

Durionoideae Ren-Gang Zhang, **subfam. nov. Type**: *Durio* Adans. **Description**: trees; simple, pinnate-veined leaves; lepidote or stellate indumentum on calyx, epicalyx and in many cases on the lower leaf surface and the ovary; presence of an initially fused epicalyx; large, muricate or generally spiny, capsular, five-locular fruits; large seeds mostly covered at least in part with an aril or sometimes with a fleshy outgrowth at the end of the funicle; flat, fleshy or foliaceous cotyledons; usually $2n = 56$ chromosomes. **Distribution**: Southeast Asia (tropical). **One tribe**: Durioneae Becc. **Six genera**: *Boschia* Korth., *Coelostegia* Benth., *Cullenia* Wight, *Durio* Adans., *Kostermansia* Soegeng, and *Neesia* Blume. **Note**: The classification of Durionoideae is nearly identical to that of Durioneae[9], with minor revisions to the chromosome number[10]. Recently, Colli-Silva et al. positioned *Neesia* as sister to Helicteroideae s.str. based on the Angiosperms353 target capture data from the Plant and Fungal Trees of Life project[11], challenging the monophyly of Durioneae[9]. However, the much shorter sequence length recovered for *Neesia* (84,732 bp compared to 178,803–283,283 bp for other Durioneae species) may have introduced bias in the phylogenetic analysis. Whole-genome sequencing for *Neesia* is strongly suggested to further resolve its phylogenetic placement and evolutionary history and confirm the monophyly of Durionoideae.

Supplementary Note 5: Limitations of the study

In the present study, while we have made significant progress in unraveling the complex evolutionary history of the Malvaceae family through polyploidy and dysploidy events, there are also certain limitations we must acknowledge. First, our analysis is primarily based on the genomic data available for the 11 representative species, which cover all nine subfamilies, but may not represent the full range of diversity of the family, particularly for subfamilies with a single representative genome. This could potentially limit the generalizability of our findings to all members of the Malvaceae. Second, the reconstruction of ancestral karyotypes and the inference of paleopolyploidy events rely on comparative genomic approaches, which, despite their robustness, depend on the accuracy of the underlying genome assemblies and the resolution of phylogenetic methods employed. Third, due to the prevalence and high degree of both ILS and introgression in the complex allopolyploidy process, there are many phylogenetic uncertainties, especially in the two "anomaly zones" (**Fig. 3**). The phylogenetic relationships of the subgenomes/lineages thus allow for alternative scenarios. Nevertheless, the impact of these uncertainties on the reconstruction of reticulate polyploidization events (e.g., Mal-α and Mal-β) is minimal, as our conclusions are supported by consistent evidence from multiple sources. Future work should integrate more ecological, geographical and environmental data to understand the significance of the observed patterns in the context of past and current environmental changes. Despite these limitations, our findings provide valuable insights into the evolutionary dynamics of the Malvaceae family and the evolutionary importance of polyploidy and dysploidy.

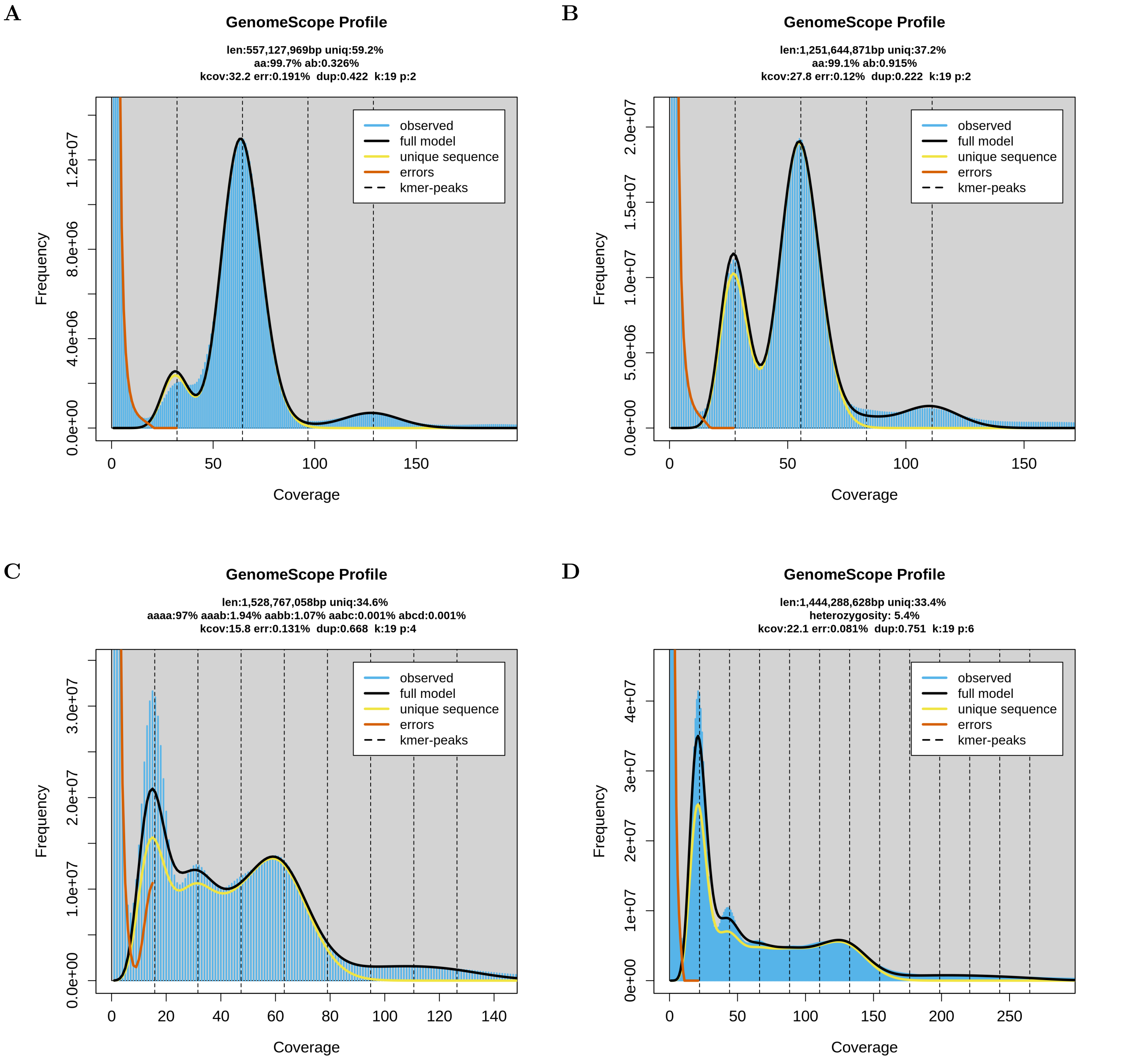

Supplementary Fig. 1. Genomic characteristics including genome size and heterozygosity rate, estimated through *k*-mer profile with GenomeScope2. (**A**) *Diplodiscus trichospermus*; (**B**) *Pterospermum kingtungense*; (**C**) *Craigia yunnanensis*; and (**D**) *Reevesia pubescens*.

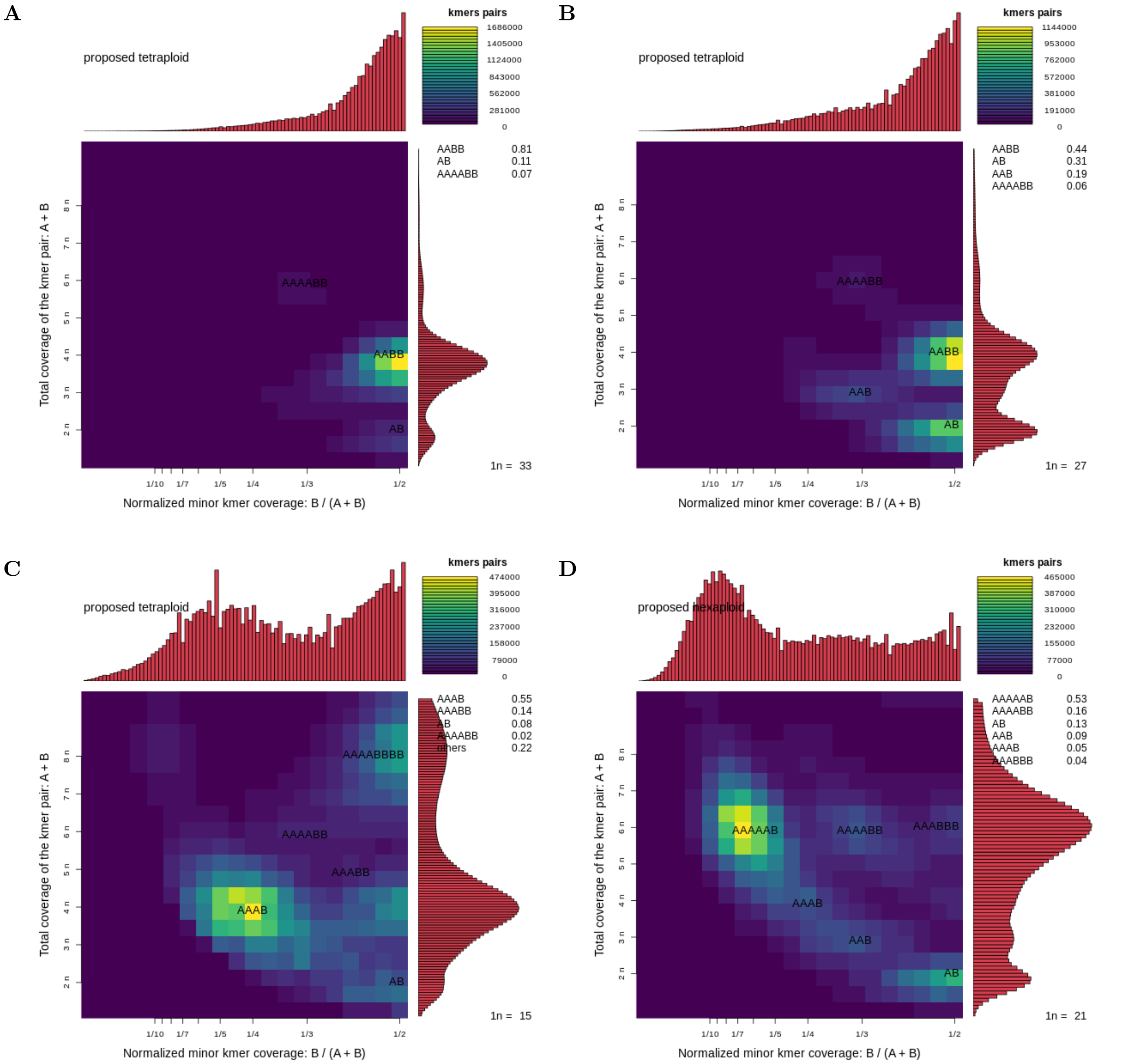

Supplementary Fig. 2. Ploidy level estimation using smudge plots. (**A**) *Diplodiscus trichospermus*; (**B**) *Pterospermum kingtungense*; (**C**) *Craigia yunnanensis*; and (**D**) *Reevesia pubescens*. The initial 'tetraploid' estimates for *D. trichospermus* and *P. kingtungense* (**A**–**B**) were incorrect, perhaps due to low heterozygosity, and was revisited as 'diploid' based on the inference from their *k*-mer profiles (**Supplementary Fig. 1**).

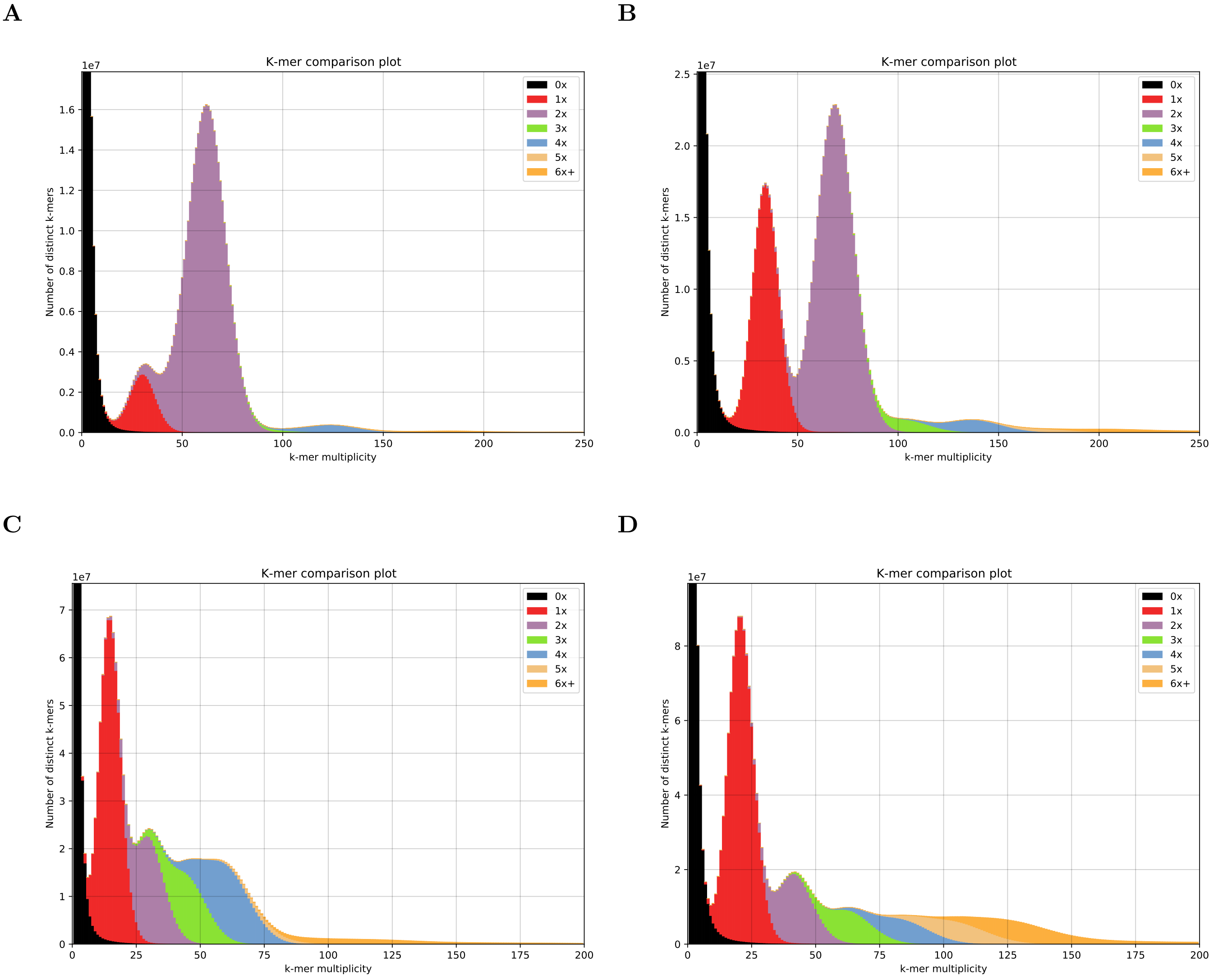

Supplementary Fig. 3. Comparison of *k*-mer spectra between haplotype-resolved assemblies and HiFi reads using KAT. (**A**) *Diplodiscus trichospermus*; (**B**) *Pterospermum kingtungense*; (**C**) *Craigia yunnanensis*; and (**D**) *Reevesia pubescens*.

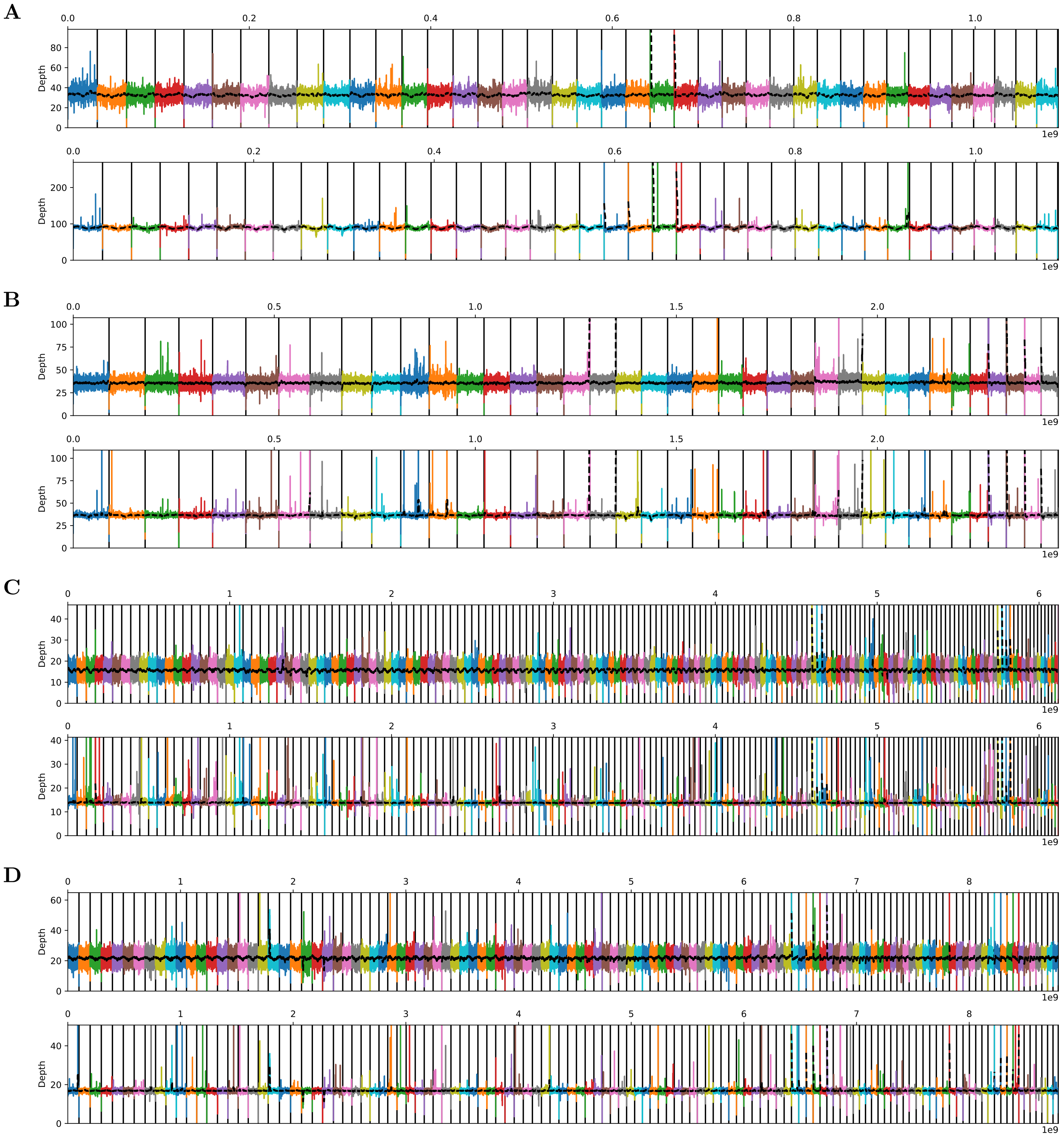

Supplementary Fig. 4. Coverage depth analysis of HiFi reads and short reads against haplotype-resolved assemblies. (**A**) *Diplodiscus trichospermus*; (**B**) *Pterospermum kingtungense*; (**C**) *Craigia yunnanensis*; and (**D**) *Reevesia pubescens*. The uniformity of the coverage depths does not indicate redundancies of haplotigs or large-scale homologous collapses.

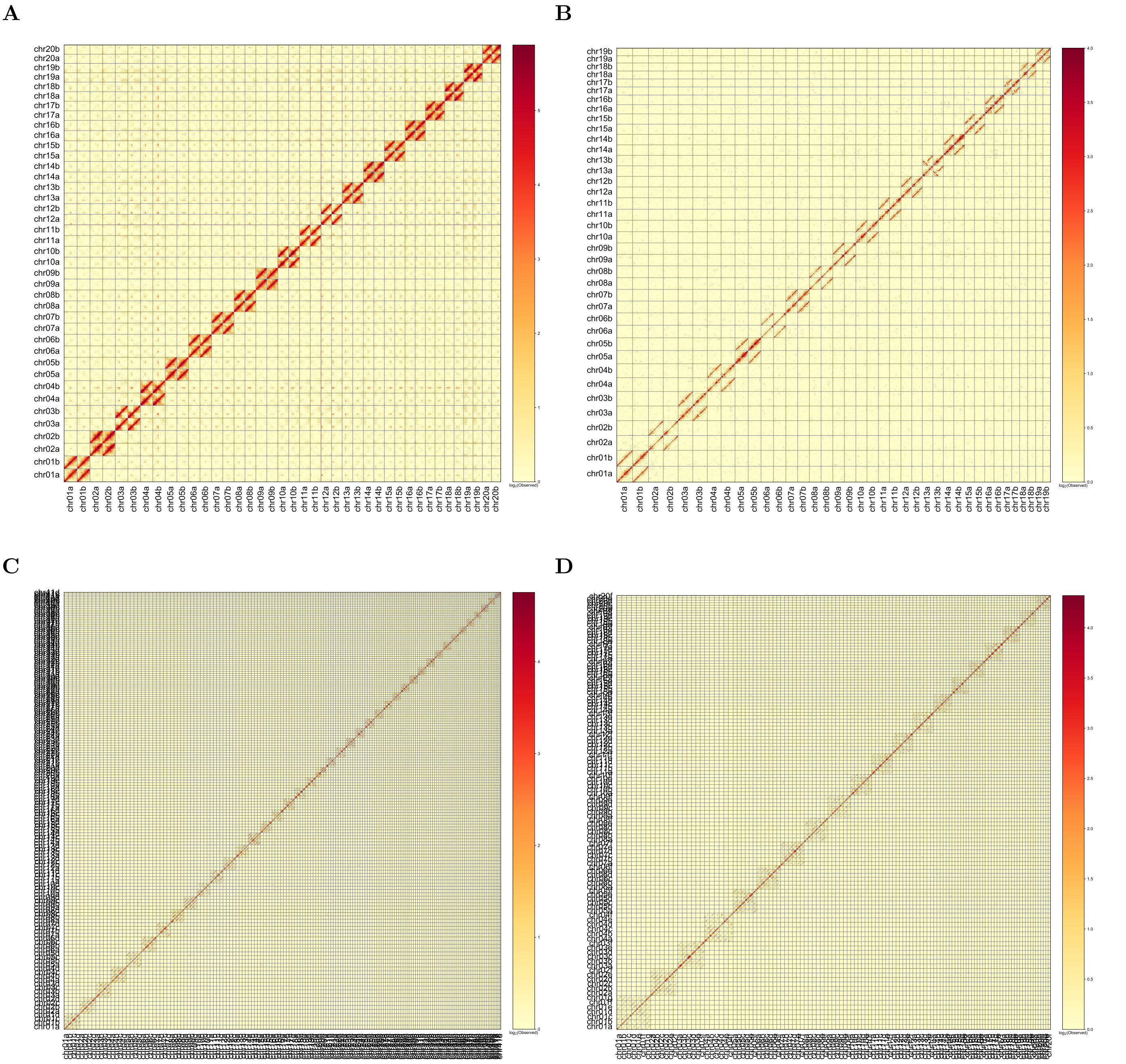

Supplementary Fig. 5. Hi-C interaction heatmap for haplotype-resolved assemblies. (**A**) *Diplodiscus trichospermus*; (**B**) *Pterospermum kingtungense*; (**C**) *Craigia yunnanensis*; and (**D**) *Reevesia pubescens*.

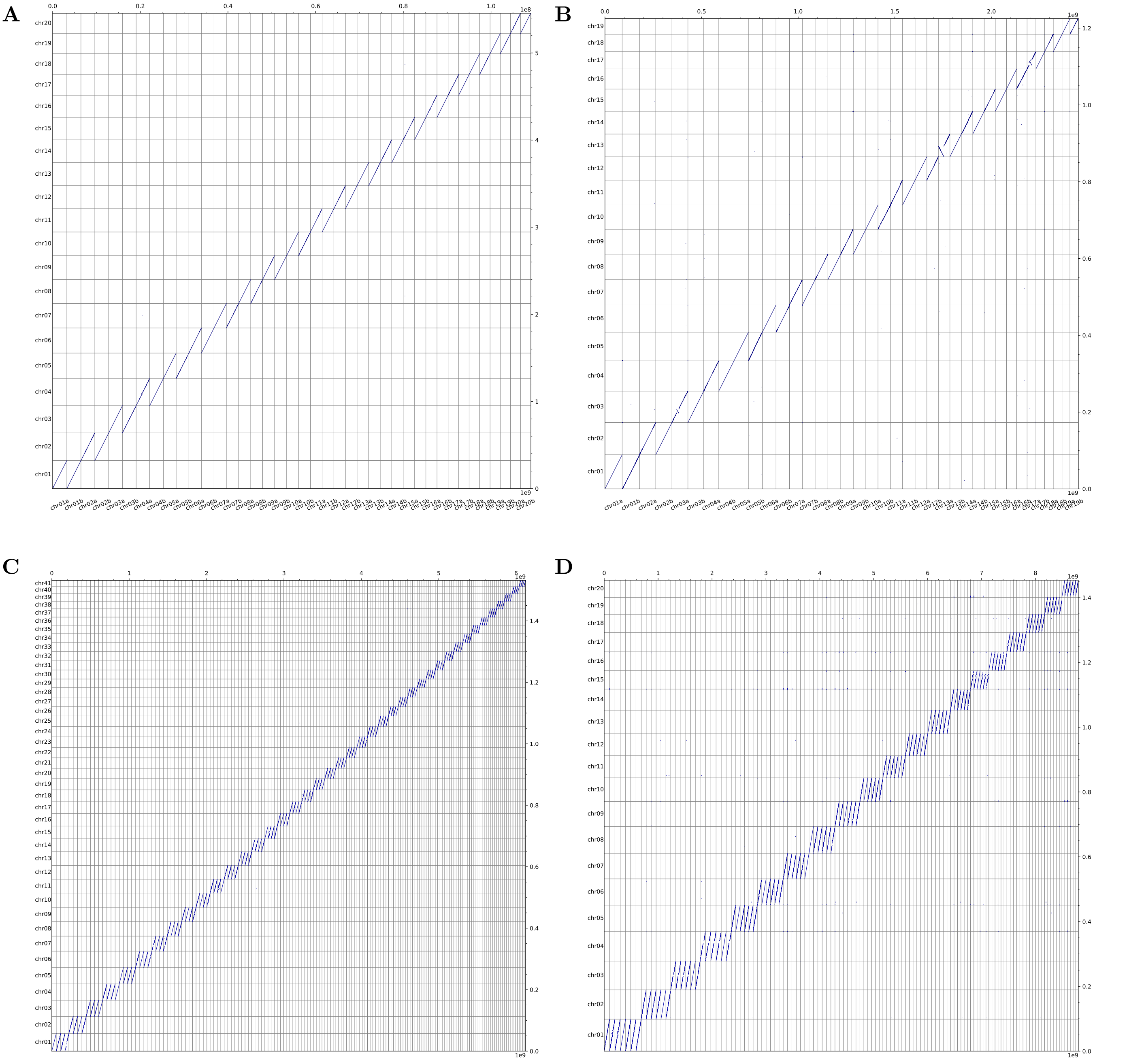

Supplementary Fig. 6. Alignments between the primary assembly (y-axis) and the haplotype-resolved assembly (x-axis). (**A**) *Diplodiscus trichospermus*; (**B**) *Pterospermum kingtungense*; (**C**) *Craigia yunnanensis*; and (**D**) *Reevesia pubescens*.

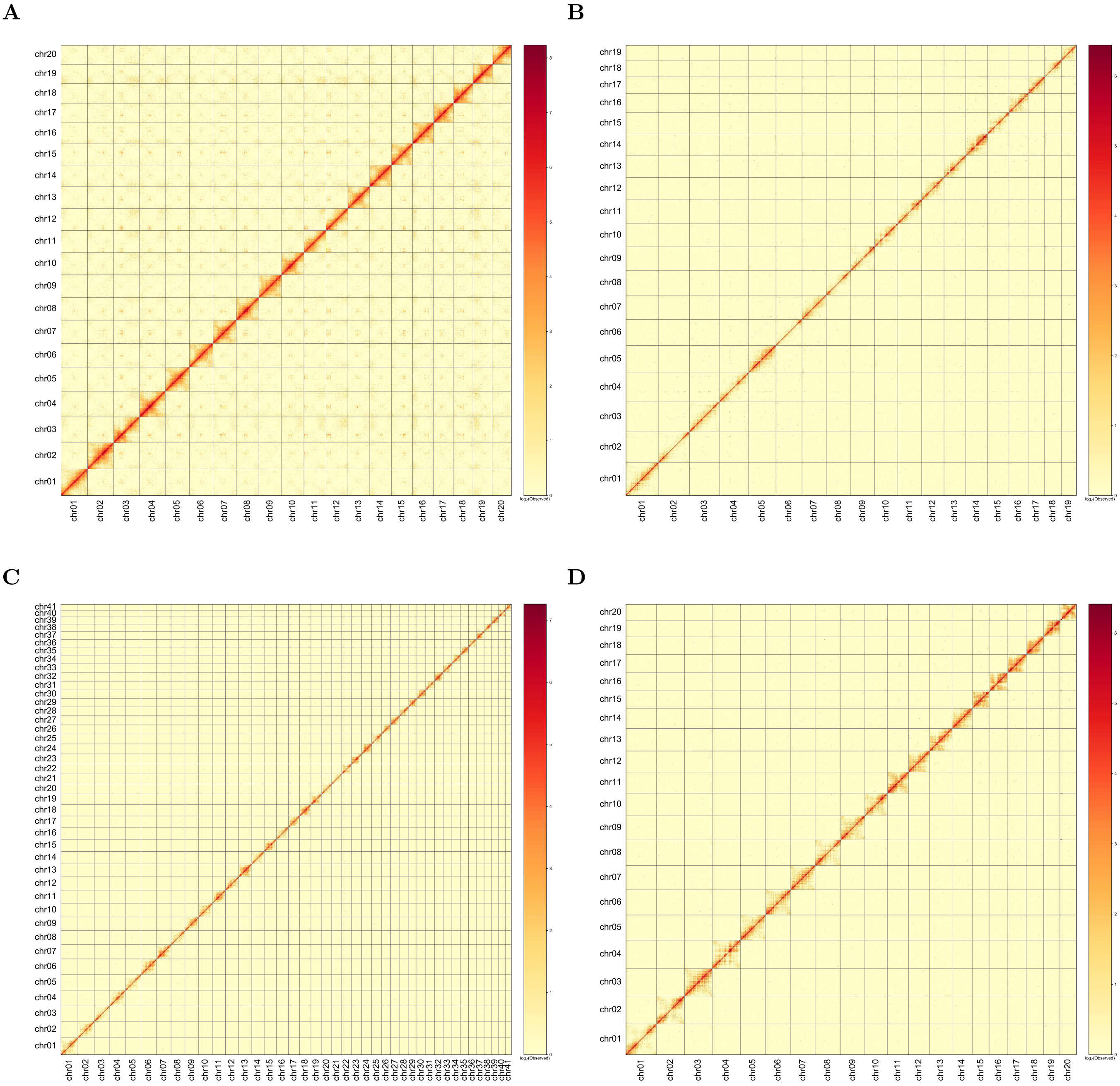

Supplementary Fig. 7. Hi-C interaction heatmaps for the primary assembly. (**A**) *Diplodiscus trichospermus*; (**B**) *Pterospermum kingtungense*; (**C**) *Craigia yunnanensis*; and (**D**) *Reevesia pubescens*.

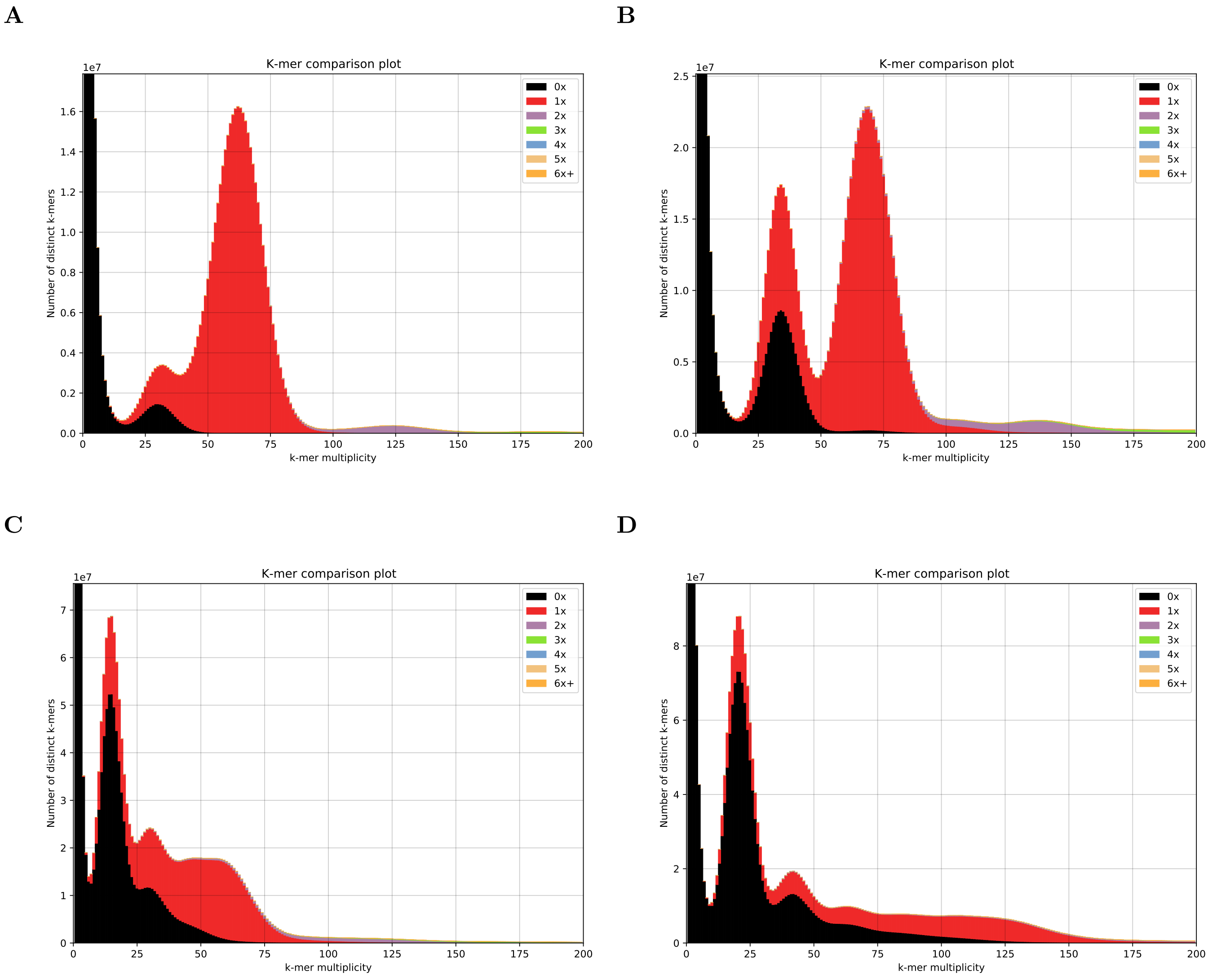

Supplementary Fig. 8. ***K*-mer analysis comparing primary assemblies and HiFi reads using KAT.** (**A**) *Diplodiscus trichospermus*; (**B**) *Pterospermum kingtungense*; (**C**) *Craigia yunnanensis*; and (**D**) *Reevesia pubescens*.

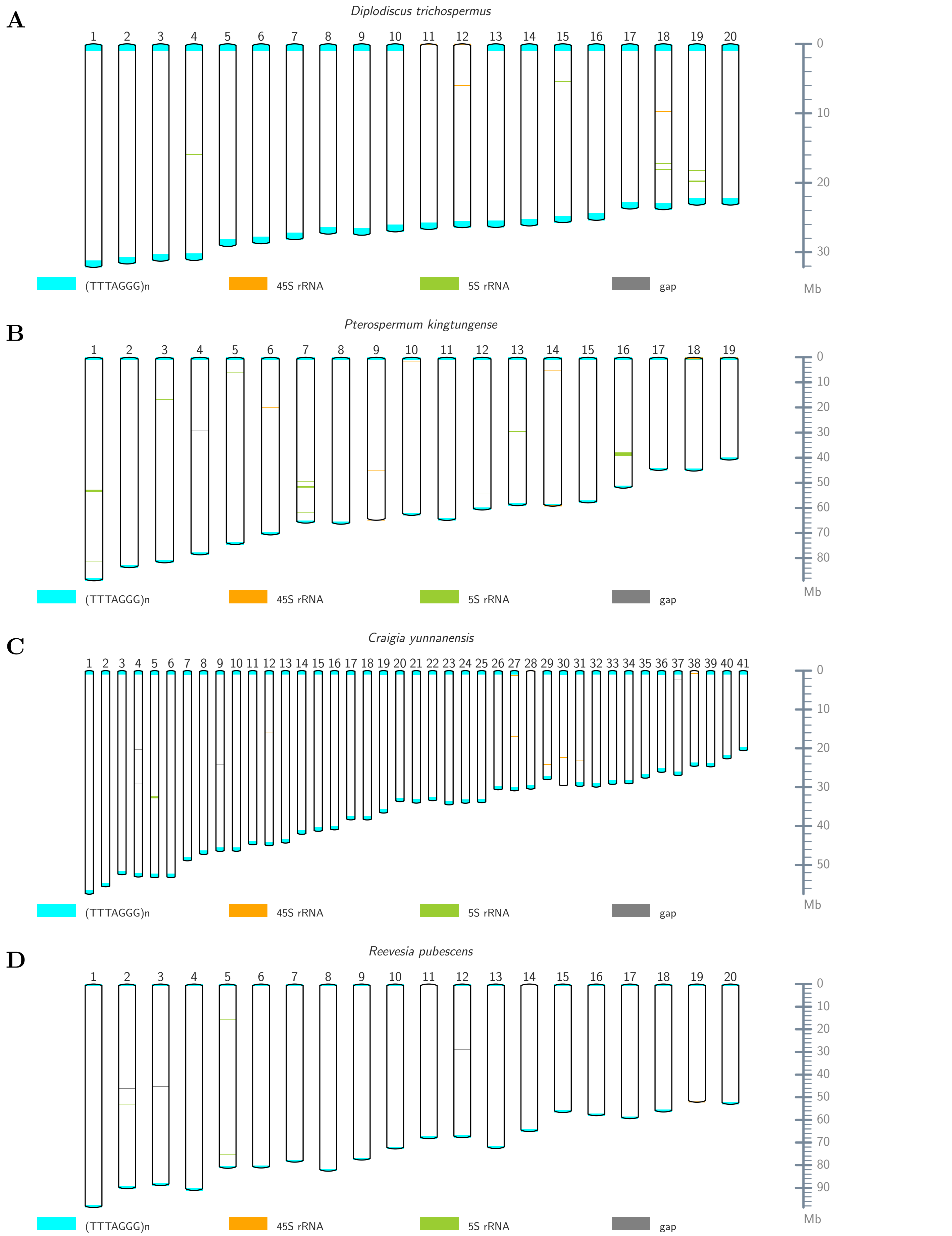

Supplementary Fig. 9. The distribution of telomeric (TTTAGGG)$_n$ repeats, 45S rDNAs, 5S rDNAs and gaps in the four primary assemblies. (**A**) *Diplodiscus trichospermus*; (**B**) *Pterospermum kingtungense*; (**C**) *Craigia yunnanensis*; and (**D**) *Reevesia pubescens*.

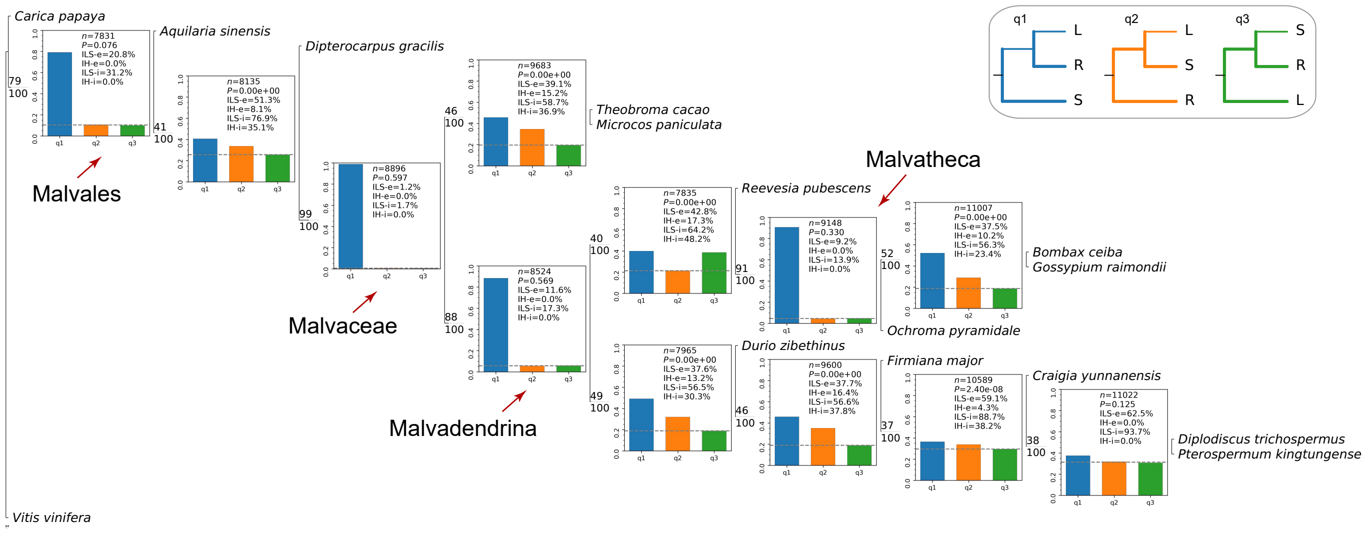

Supplementary Fig. 10. Nuclear phylogeny reconstructed with the coalescence-based method, employing ASTRAL for tree construction and PhyTop for visualization, showing the discordance of gene trees. The numbers above the branches represent the concordance percentages between gene and species trees and the numbers below the branches represent the local posterior probabilities calculated by ASTRAL. Bar plots at the nodes represent the frequency of three gene tree topologies (q1, q2 and q3) calculated in ASTRAL. In the bar plots, *n* is the number of gene trees; *P* is the *P* value of a χ^2 test to test whether the three topology numbers fit the expectation when only ILS occurred (ILS-only expects q2 = q3, while introgression/hybridization (IH) expects q2 > q3 or q2 < q3); ILS-e and IH-e, the discordant gene trees that can be explained by ILS and IH, respectively; ILS-i and IH-i, the indices to account for the extent of ILS and IH, respectively. Bar, 1.0 coalescent unit. The arrows indicate highly confident nodes having few gene tree discordances (q2 and q3) and low ILS and IH indices (ILS-i < 0.3 and IH-i < 0.05). Source data are provided as a Source Data file.

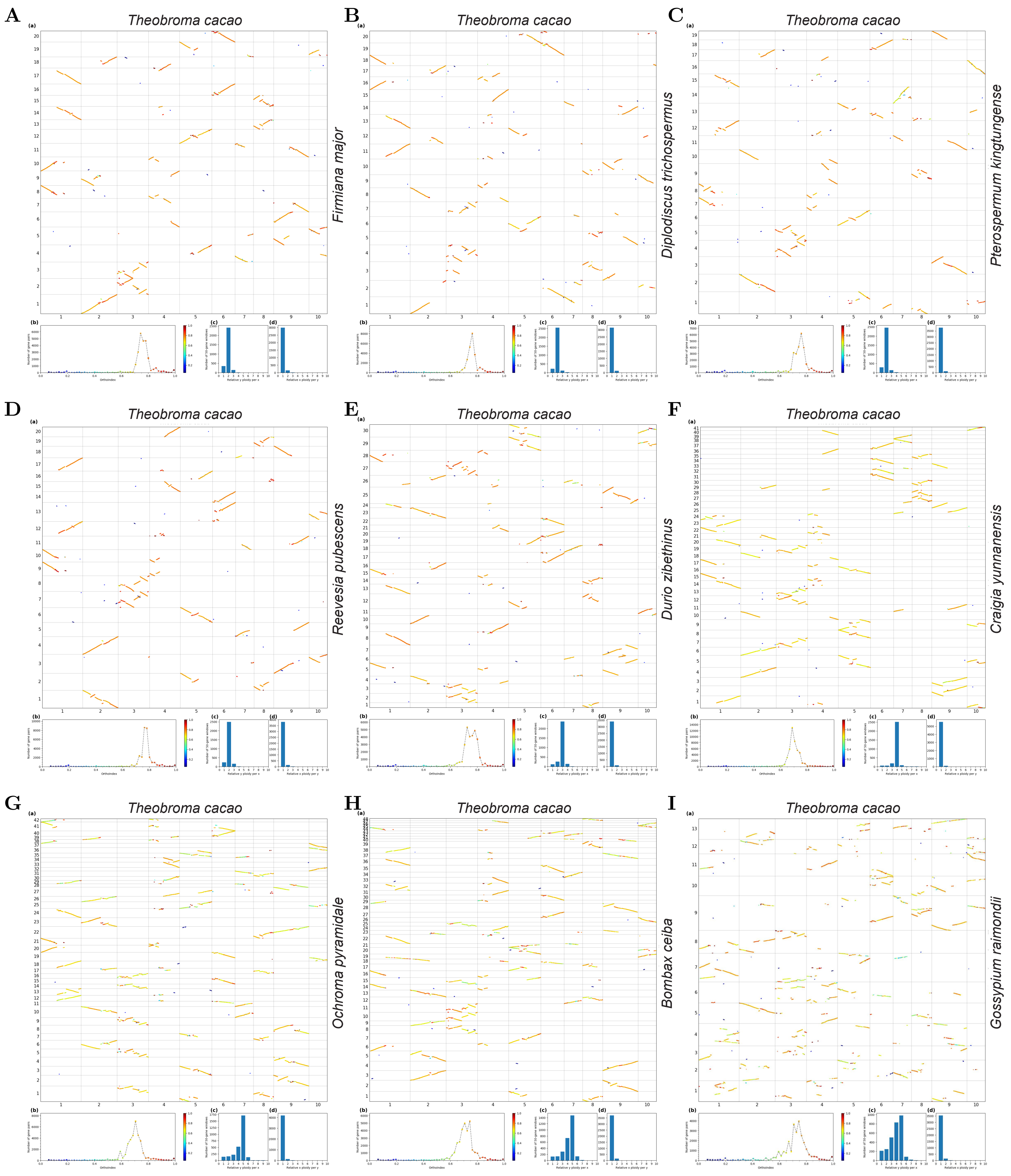

Supplementary Fig. 11. Orthologous synteny patterns between *Theobroma cacao* and nine Malvadendrina paleopolyploids using Orthology Index-colored dot plots to demonstrate patterns of relative ploidy. (**A–I**) Orthologous synteny patterns between *Theobroma cacao* and *Firmiana major* (**A**), *Diplodiscus trichospermus* (**B**), *Pterospermum kingtungense* (**C**), *Reevesia pubescens* (**D**), *Durio zibethinus* (**E**), *Craigia yunnanensis* (**F**), *Ochroma pyramidale* (**G**), *Bombax ceiba* (**H**) and *Gossypium raimondii* (**I**). Subplots include (**a**) dot plots colored by Orthology Index to show homologous gene pairs across chromosomes, (**b**) a histogram showing the distribution of Orthology Index against the number of homologous gene pairs, and (**c–d**) analyses of orthologous synteny depth (indicative of relative ploidy) across 50-gene windows of the x- (c) or y-axis (d) in subplot a. Points with Orthology Index <0.05 were considered to be from very ancient polyploidy events and therefore not shown. Source data are provided as a Source Data file.

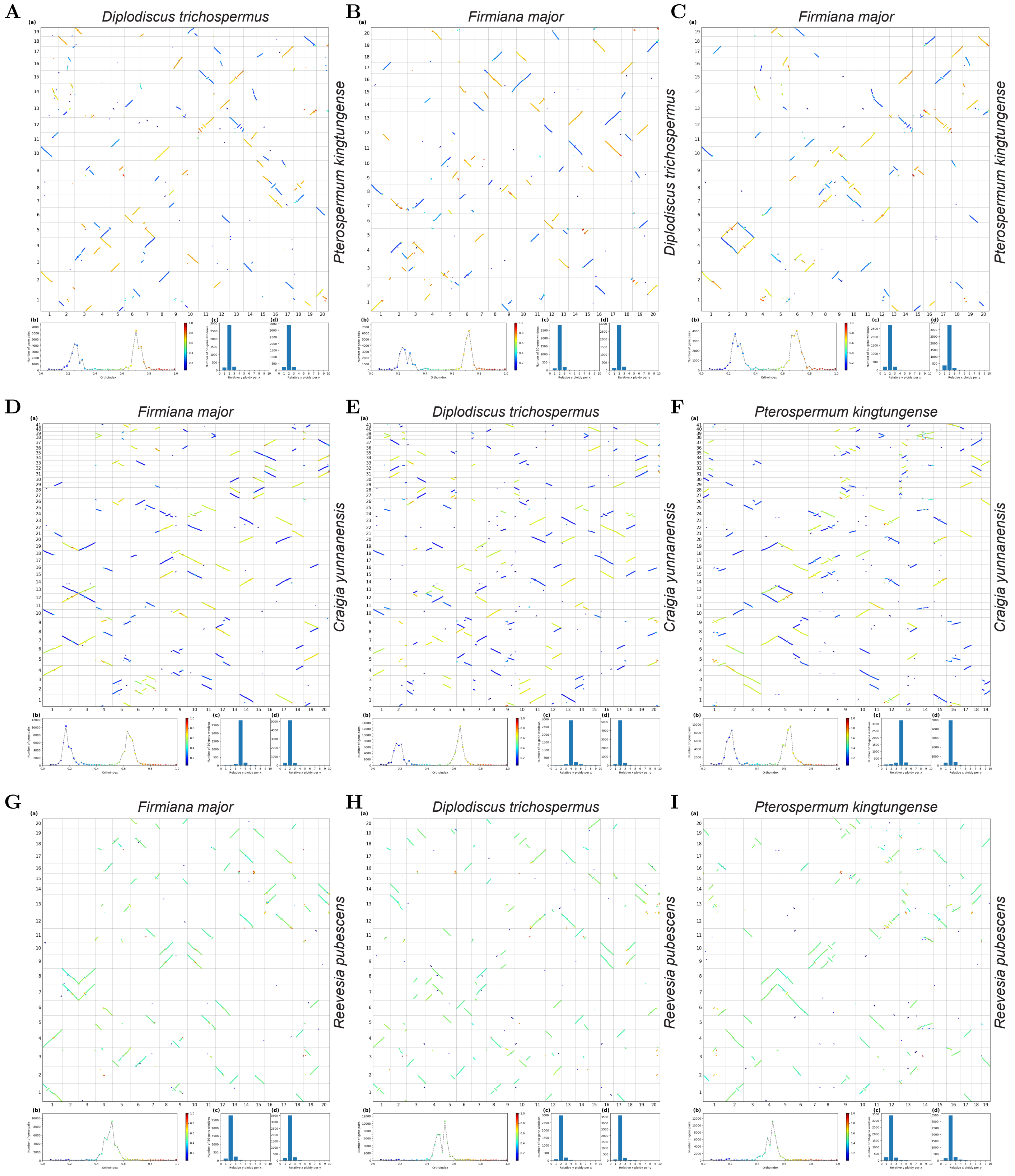

Supplementary Fig. 12. Orthologous synteny patterns to show orthologous relationships between subgenomes of paleotetraploids. (**A**–**C**) Orthologous synteny patterns across three paleotetraploids. (**D**–**F**) Orthologous synteny patterns between *Craigia yunnanensis* and the three paleotetraploids. (**G**–**I**) Orthologous synteny patterns between *Reevesia pubescens* and the three paleotetraploids. Subplots include (**a**) dot plots colored by Orthology Index to show homologous gene pairs across chromosomes, (**b**) a histogram showing the distribution of Orthology Index against the number of homologous gene pairs, and (**c–d**) analyses of synteny depth across 50-gene windows of the x- (c) or y-axis (d) in subplot a. Points with Orthology Index <0.05 were considered to be from very ancient polyploidy events and therefore not shown. Source data are provided as a Source Data file.

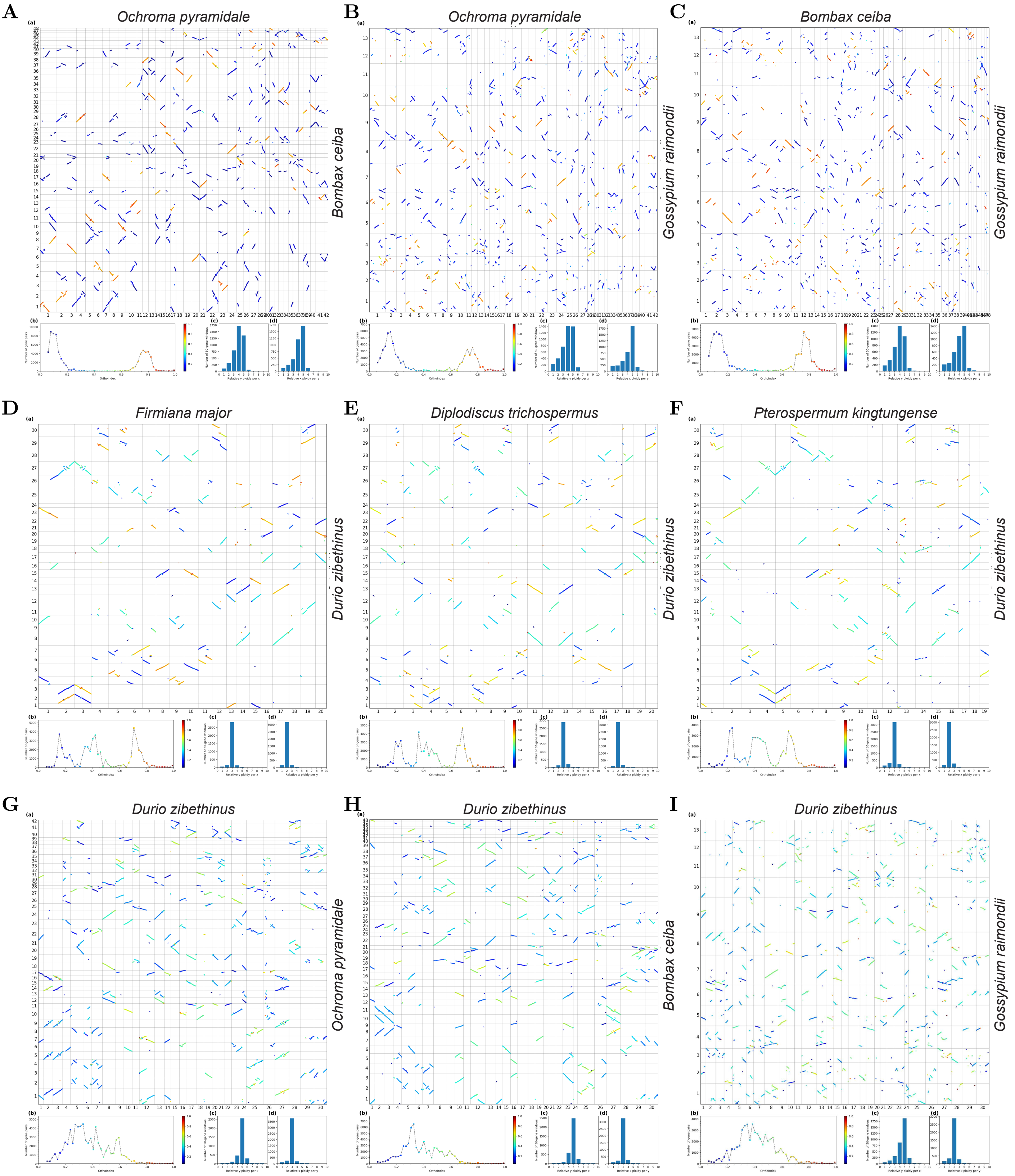

Supplementary Fig. 13. Orthologous synteny patterns to show orthologous relationships between subgenomes of paleopolyploids. (**A**–**C**) Orthologous synteny patterns across three paleodecaploids. (**D**–**F**) Orthologous synteny patterns between *Durio zibethinus* and the three paleotetraploids. (**G**–**I**) Orthologous synteny patterns between *Durio zibethinus* and the three paleodecaploids. Subplots include (**a**) dot plots colored by Orthology Index to show homologous gene pairs across chromosomes, (**b**) a histogram showing the distribution of Orthology Index against the number of homologous gene pairs, and (**c**–**d**) analyses of synteny depth across 50-gene windows of the x- (c) or y-axis (d) in subplot a. Points with Orthology Index <0.05 were considered to be from very ancient polyploidy events and therefore not shown. Source data are provided as a Source Data file.

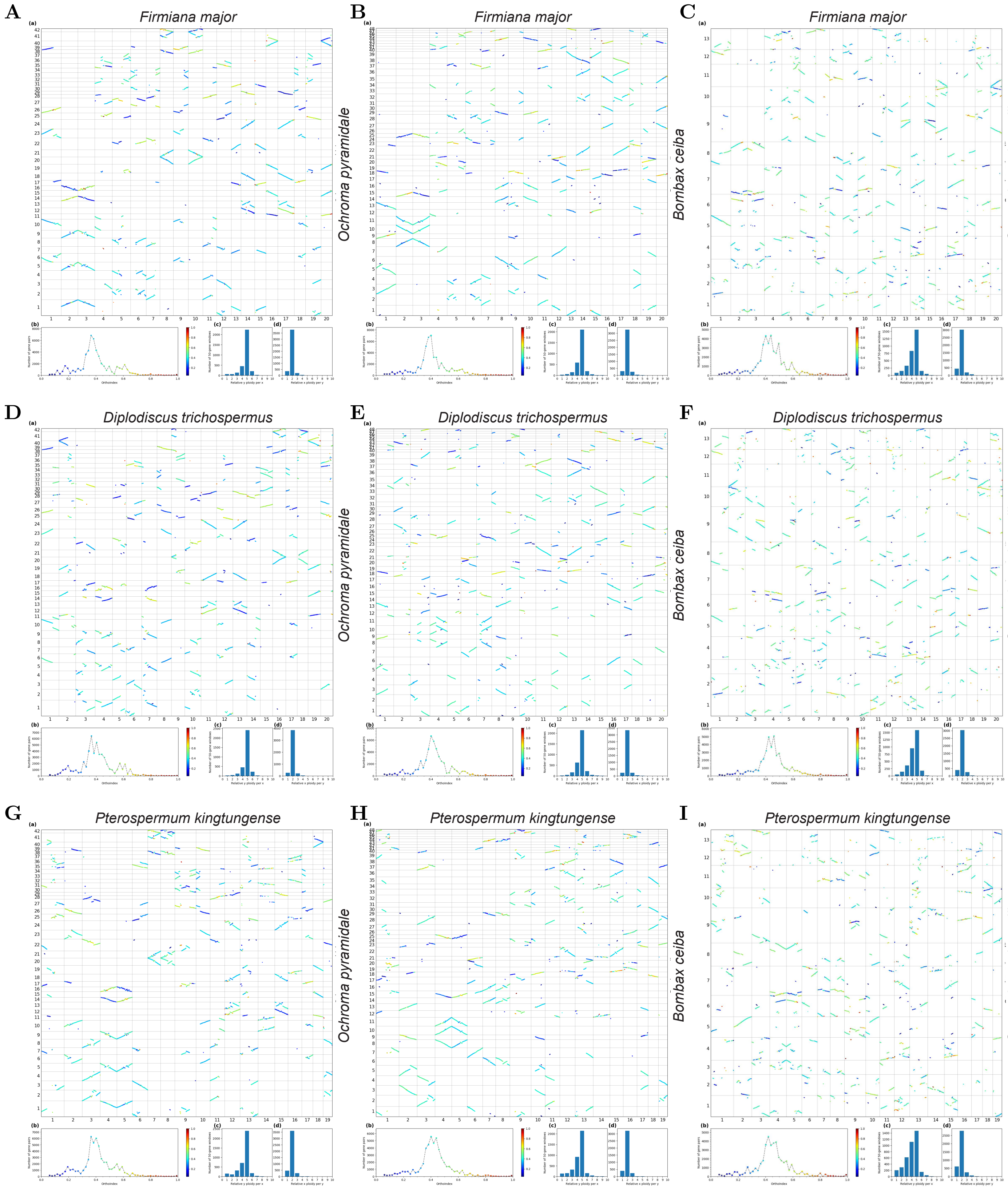

Supplementary Fig. 14. Orthologous synteny patterns to show orthologous relationships between paleotetraploids and paleodecaploids. (**A**–**I**) Orthologous synteny patterns across three paleotetraploids and three paleodecaploids. Subplots include (**a**) dot plots colored by Orthology Index to show homologous gene pairs across chromosomes, (**b**) a histogram showing the distribution of Orthology Index against the number of homologous gene pairs, and (**c**–**d**) analyses of synteny depth across 50-gene windows of the x- (c) or y-axis (d) in subplot a. Points with Orthology Index <0.05 were considered to be from very ancient polyploidy events and therefore not shown. Source data are provided as a Source Data file.

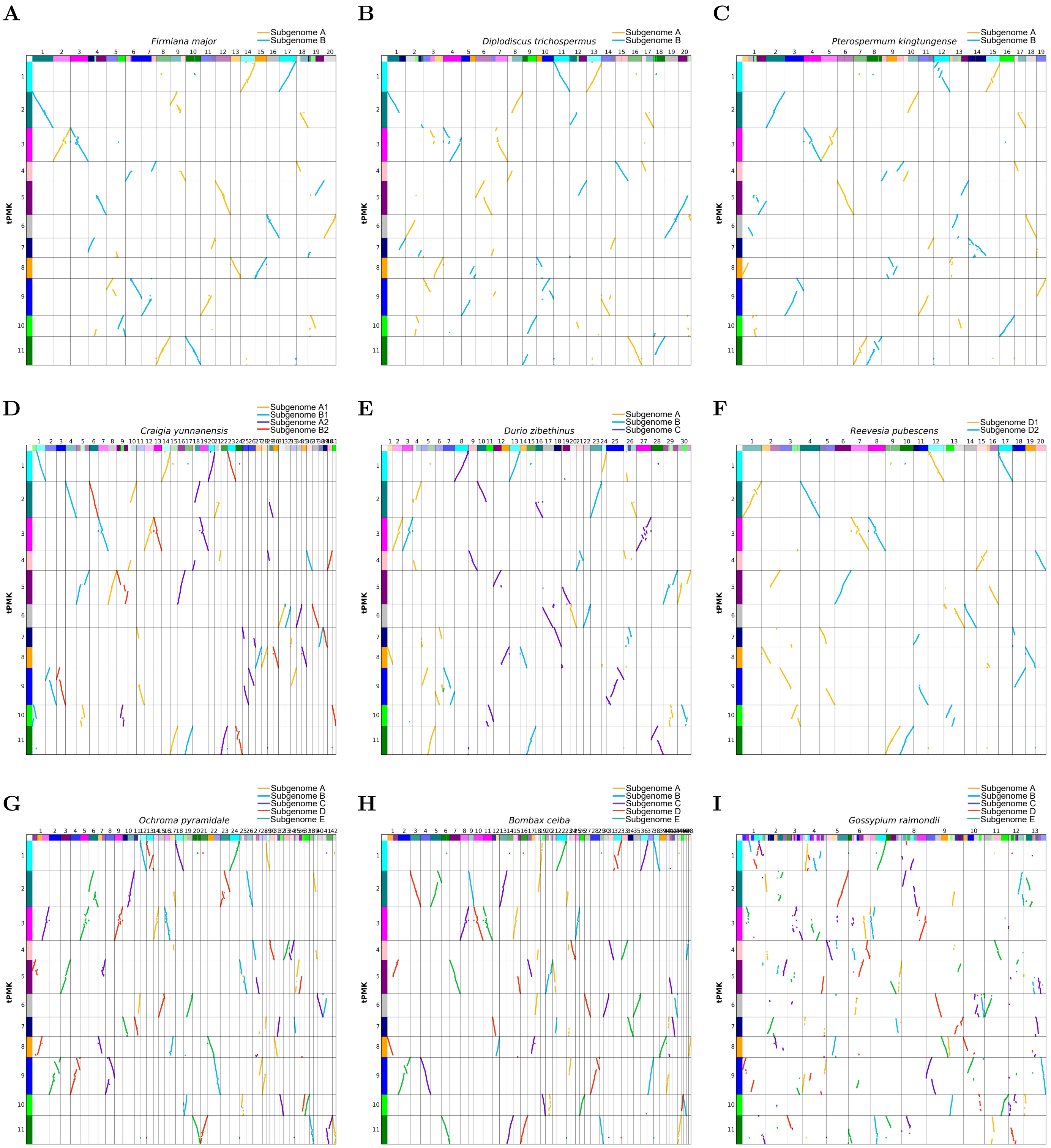

Supplementary Fig. 15. Dot plots illustrating subgenome phasing of the Malvadendrina paleopolyploids, with each dot representing an orthologous gene pair. (**A**–**I**) Subgenome assignments of *Firmiana major* (**A**), *Diplodiscus trichospermus* (**B**), *Pterospermum kingtungense* (**C**), *Craigia yunnanensis* (**D**), *Durio zibethinus* (**E**), *Reevesia pubescens* (**F**), *Ochroma pyramidale* (**G**), *Bombax ceiba* (**H**) and *Gossypium raimondii* (**I**). Source data are provided as a Source Data file.

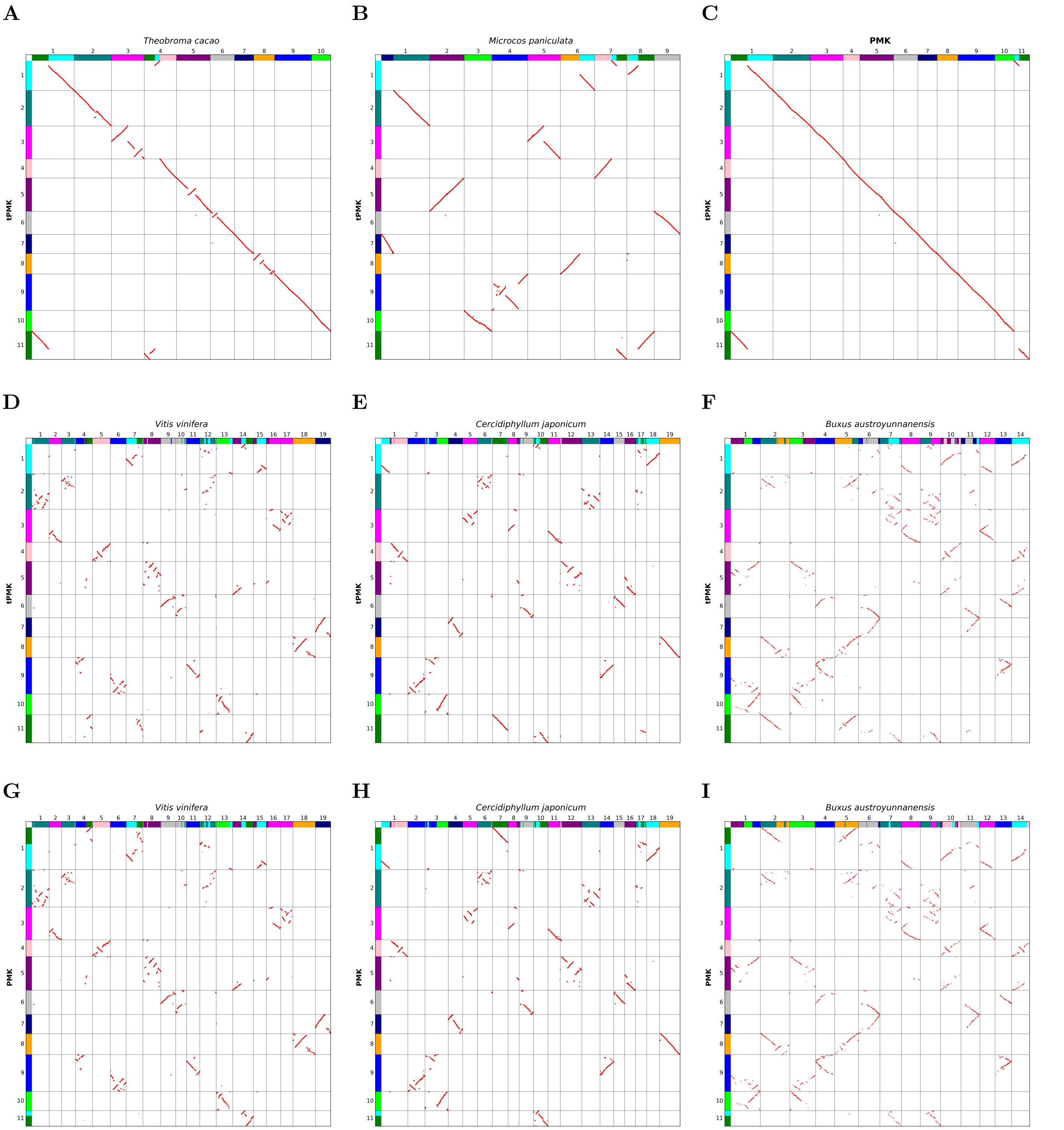

Supplementary Fig. 16. Dot plots illustrating synteny between PMK, tPMK, the diploid Byttneriina and outgroup genomes. (**A**–**C**) Synteny between tPMK and three Byttneriina genomes. (**D**–**F**) Synteny between tPMK and three outgroup genomes. (**G**–**I**) Synteny between PMK and three outgroup genomes, showing support for PMK from the three outgroups. Source data are provided as a Source Data file.

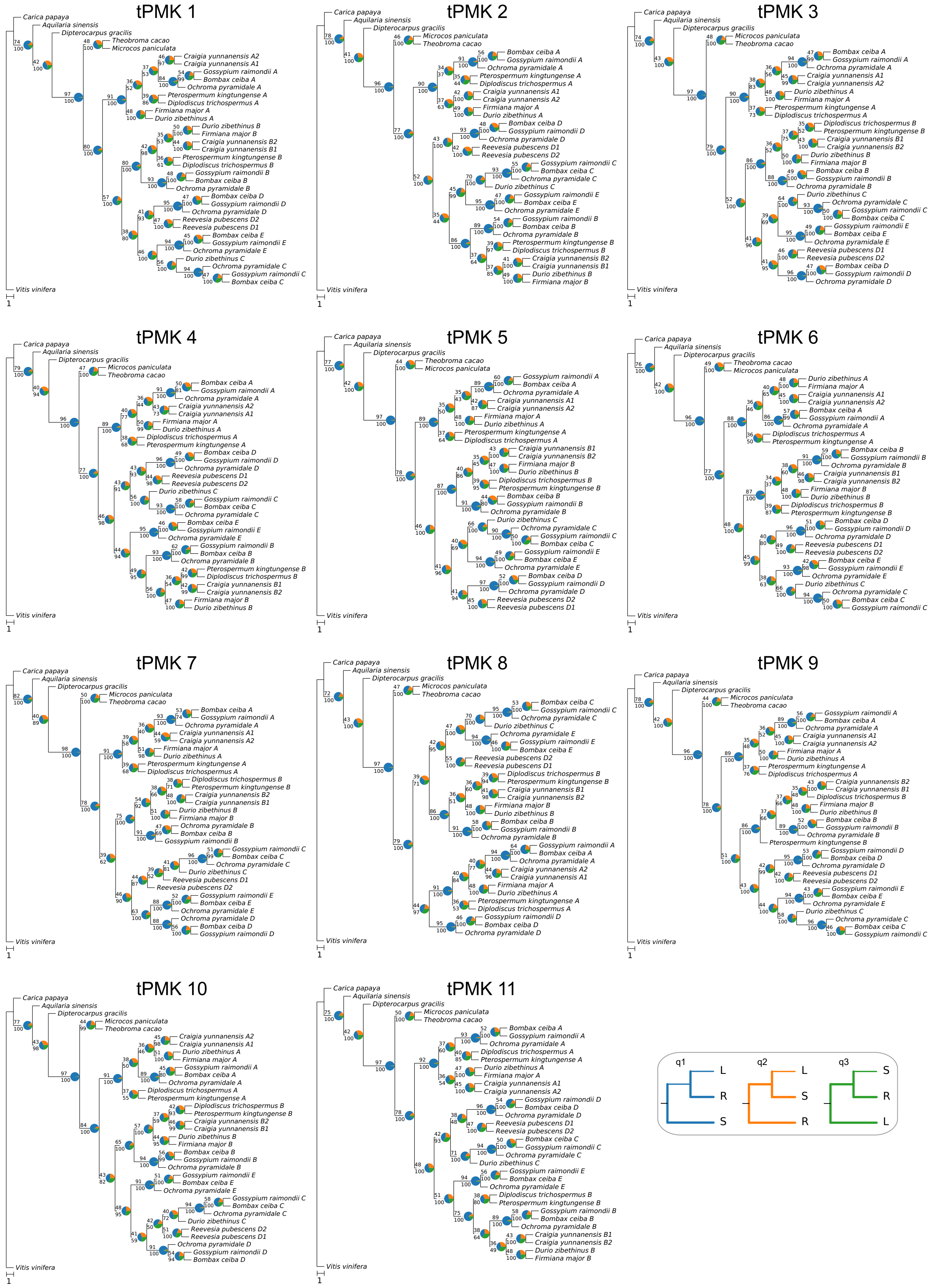

Supplementary Fig. 17. Chromosome-scale phylogenies for phased subgenomes showing phylogenetic relationships of each chromosome set. The numbers above the branches indicate the concordance percentage between gene-based and chromosome-scale trees; the numbers below the branches represent the local posterior probabilities calculated by ASTRAL. The pie charts at the nodes represent the frequencies of three gene tree topologies (q1, q2 and q3) calculated in ASTRAL. Source data are provided as a Source Data file.

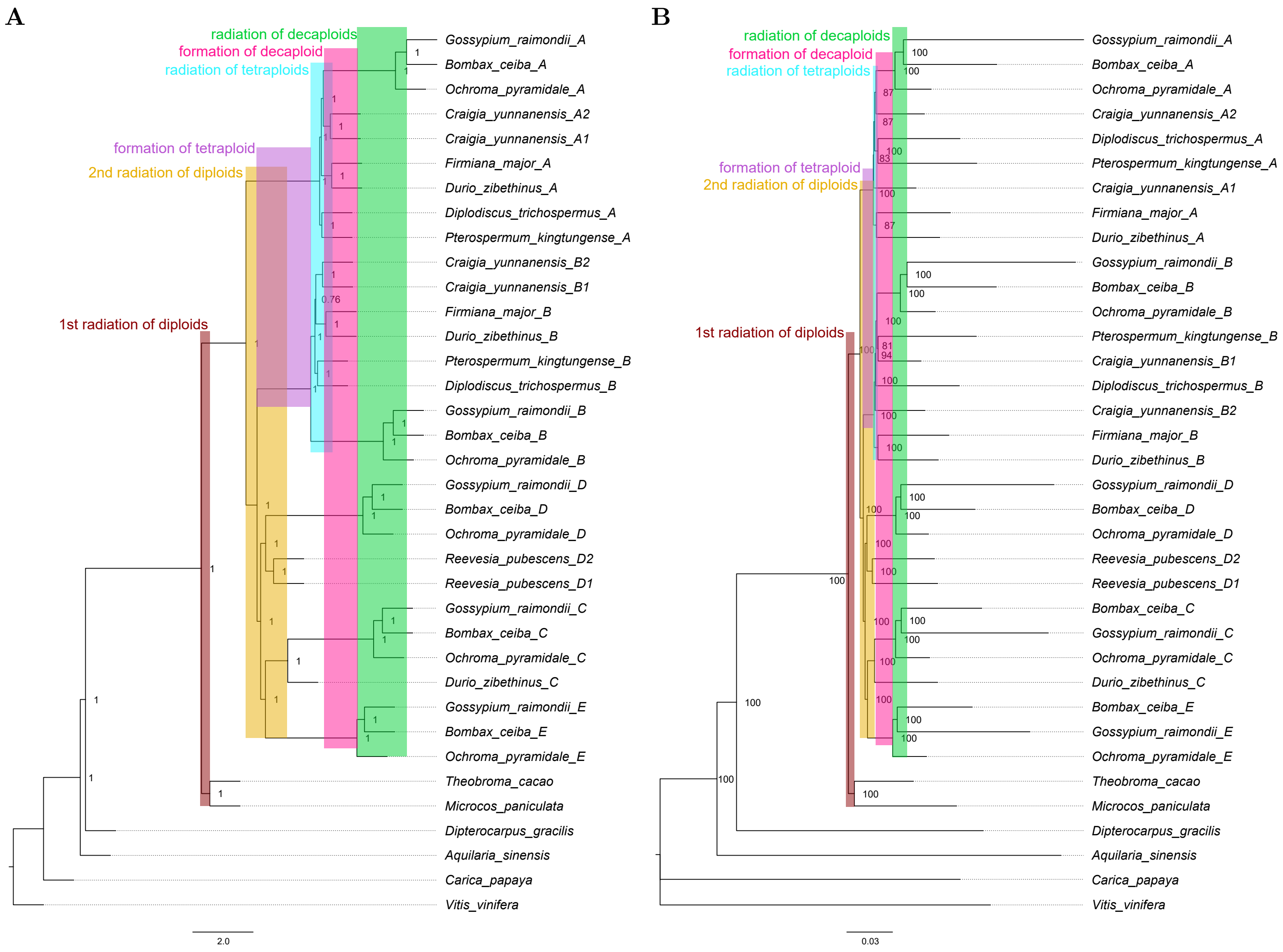

Supplementary Fig. 18. Subgenome phylogenies inferred using coalescent and concatenation methods. (**A**) The coalescent tree based on 5,194 gene trees, with up to 40% of taxa missing. The numbers at the nodes represent the local posterior probabilities calculated in ASTRAL. Bar, 2.0 coalescent units. (**B**) The maximum-likelihood tree based on a concatenated codon alignment with 1,073,289 sites and up to 20% gaps at each site. The numbers at the nodes are the bootstrap values calculated using IQ-TREE2. Bar, 0.03 nucleotide substitutions per site. Source data are provided as a Source Data file.

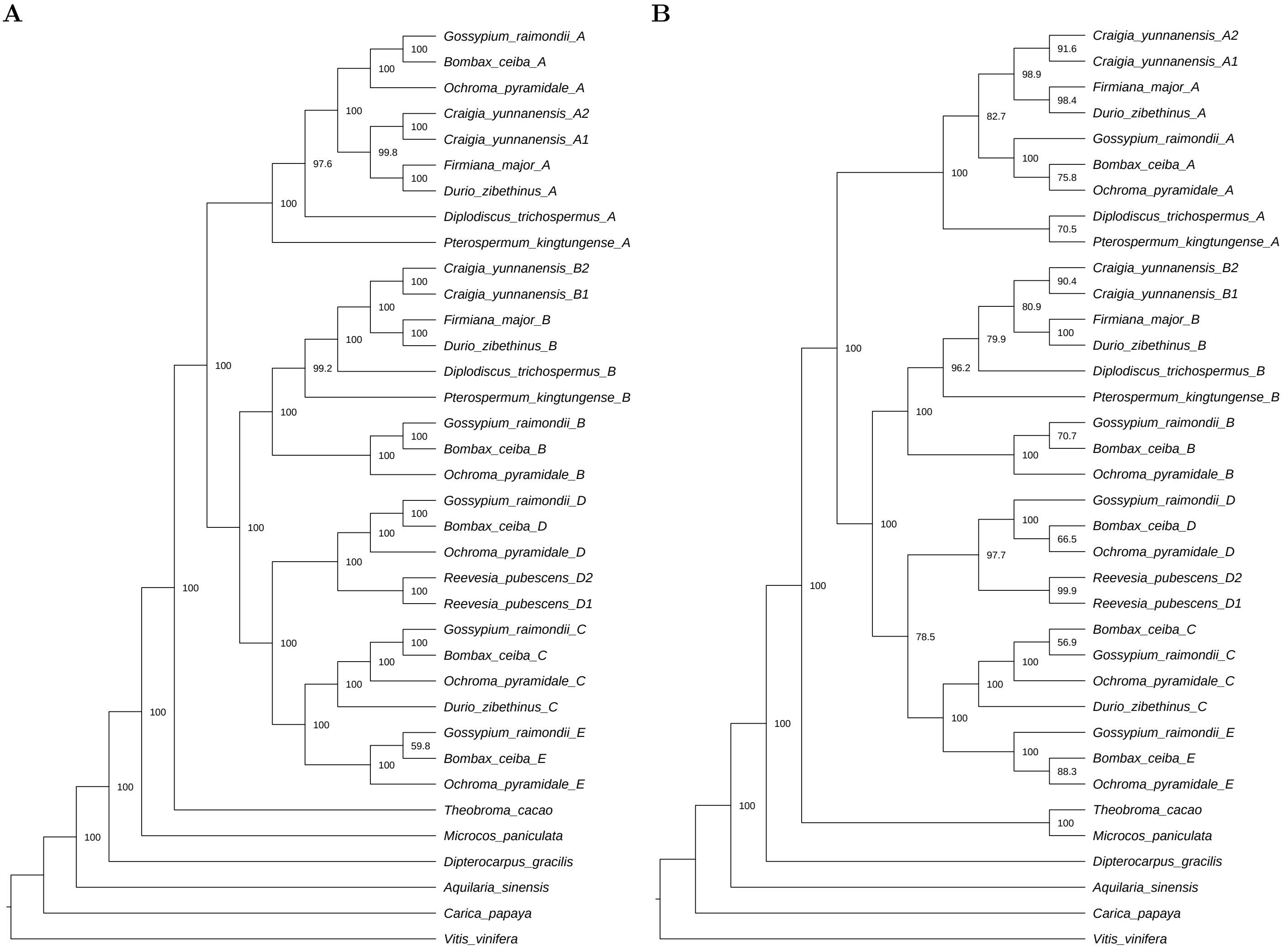

Supplementary Fig. 19. Site-based subgenome phylogenies. (**A**–**B**) Phylogenies reconstructed by CATER-pair (**A**) and CASTER-site (**B**). The trees are based on a concatenated codon alignment with 10,146,564 sites and up to 50% gaps at each site. The numbers at the nodes are local bootstraps calculated with CASTER (no branch lengths were output). Source data are provided as a Source Data file.

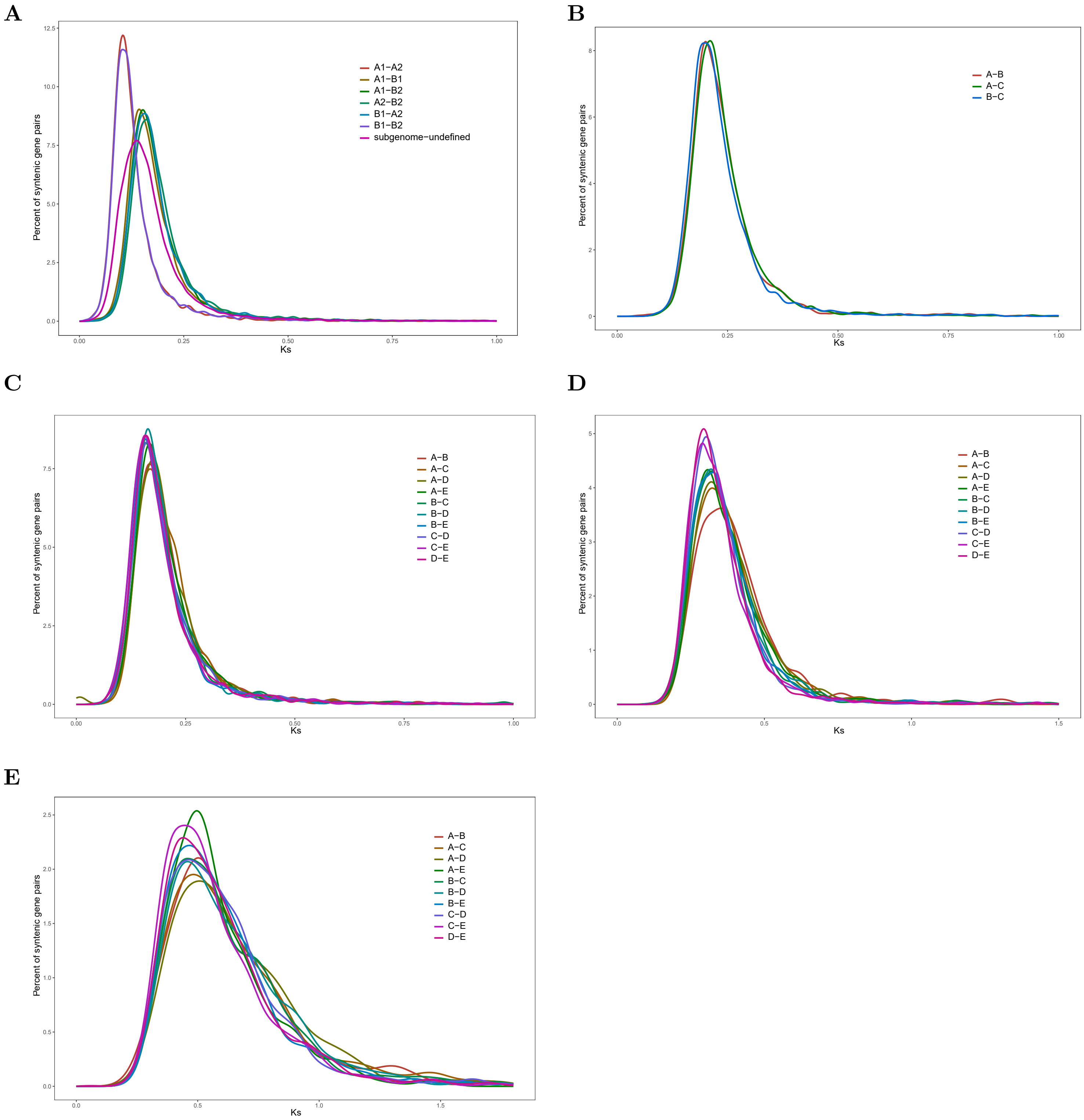

Supplementary Fig. 20. Subgenome-aware *K*s distributions. (**A**–**E**) *K*s distributions between subgenomes of *Craigia yunnanensis* (**A**), *Durio zibethinus* (**B**), *Ochroma pyramidale* (**C**), *Bombax ceiba* (**D**) and *Gossypium raimondii* (**E**). Source data are provided as a Source Data file.

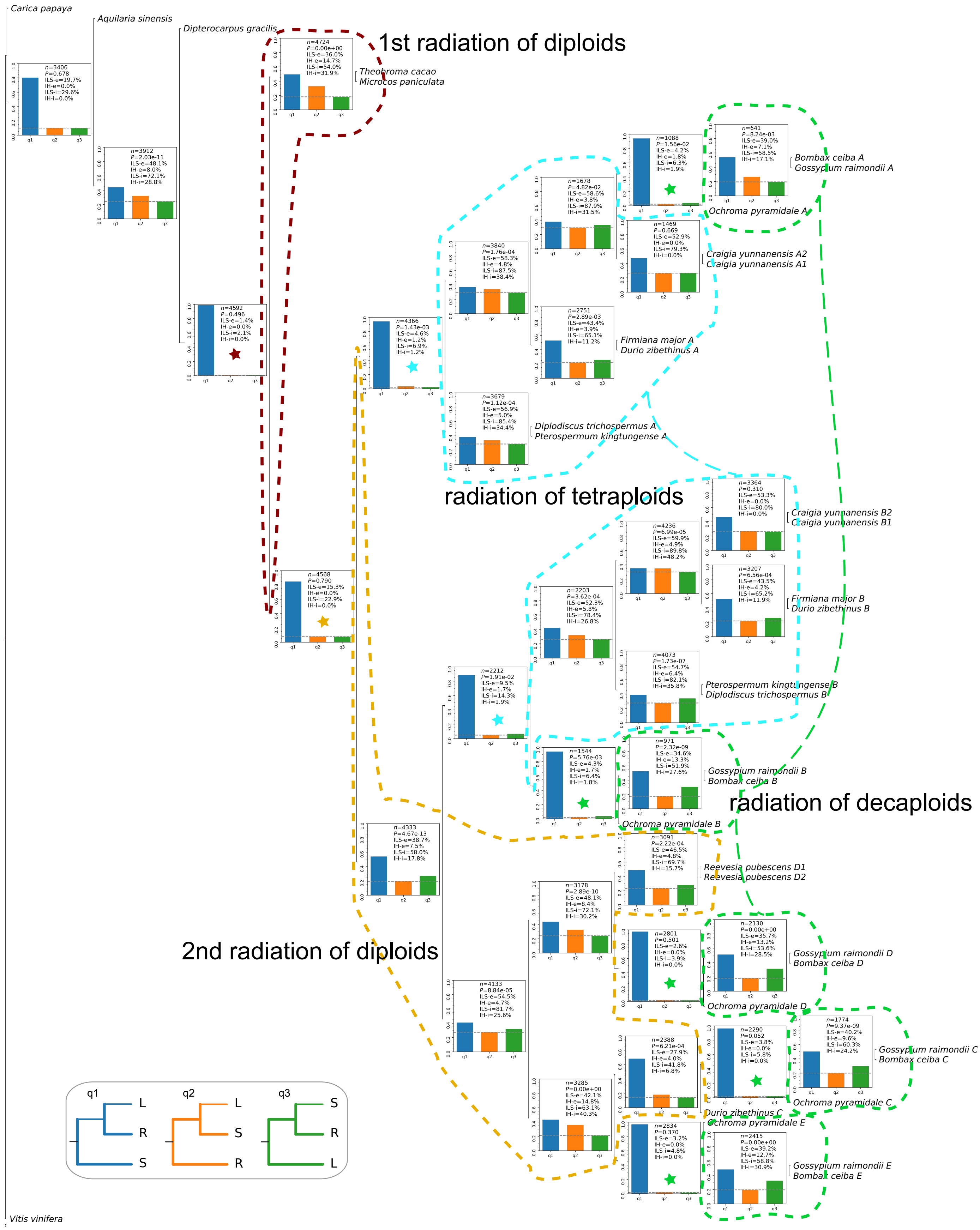

Supplementary Fig. 21. Visualization of the gene tree discordance across the coalescent-based subgenome phylogeny. The solid stars mark highly confident nodes having few gene tree discordances (q2 and q3) and low ILS and IH indices (ILS-i < 0.3 and IH-i < 0.05). Source data are provided as a Source Data file.

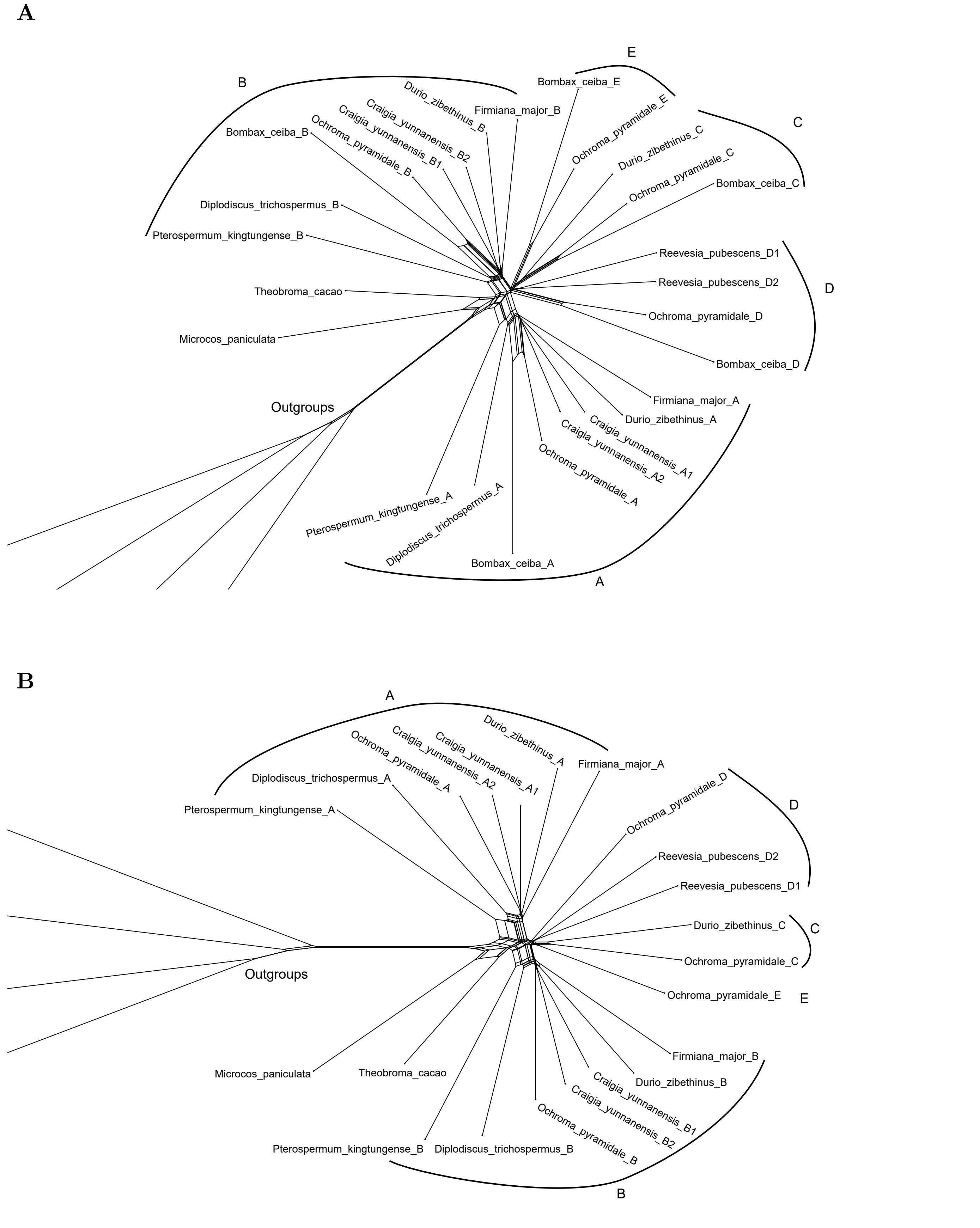

Supplementary Fig. 22. Split networks excluding subgenomes of *Bombax ceiba* and/or *Gossypium raimondii* to reduce the effect of potential long-branch attraction. (**A**) Split networks excluding subgenomes of *Gossypium raimondii*. (**B**) Split networks excluding subgenomes of *Bombax ceiba* and *Gossypium raimondii*. *Ochroma pyramidale* with the relatively short branch length was used to represent *Bombax ceiba* and *Gossypium raimondii* as they share the decapolyploidization event, and the gene tree discordance in their crown clade is low (see **Supplementary Fig. 21**). Source data are provided as a Source Data file.

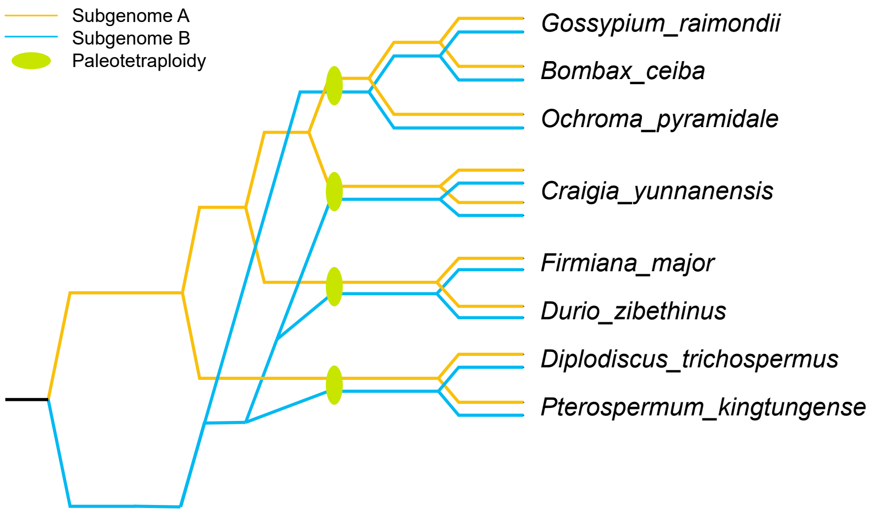

Supplementary Fig. 23. An alternative "multiple origins" hypothesis to account for discrepancies between the topology of the A and B subgenome trees, assuming the validity of the subgenome tree estimated by ASTRAL.

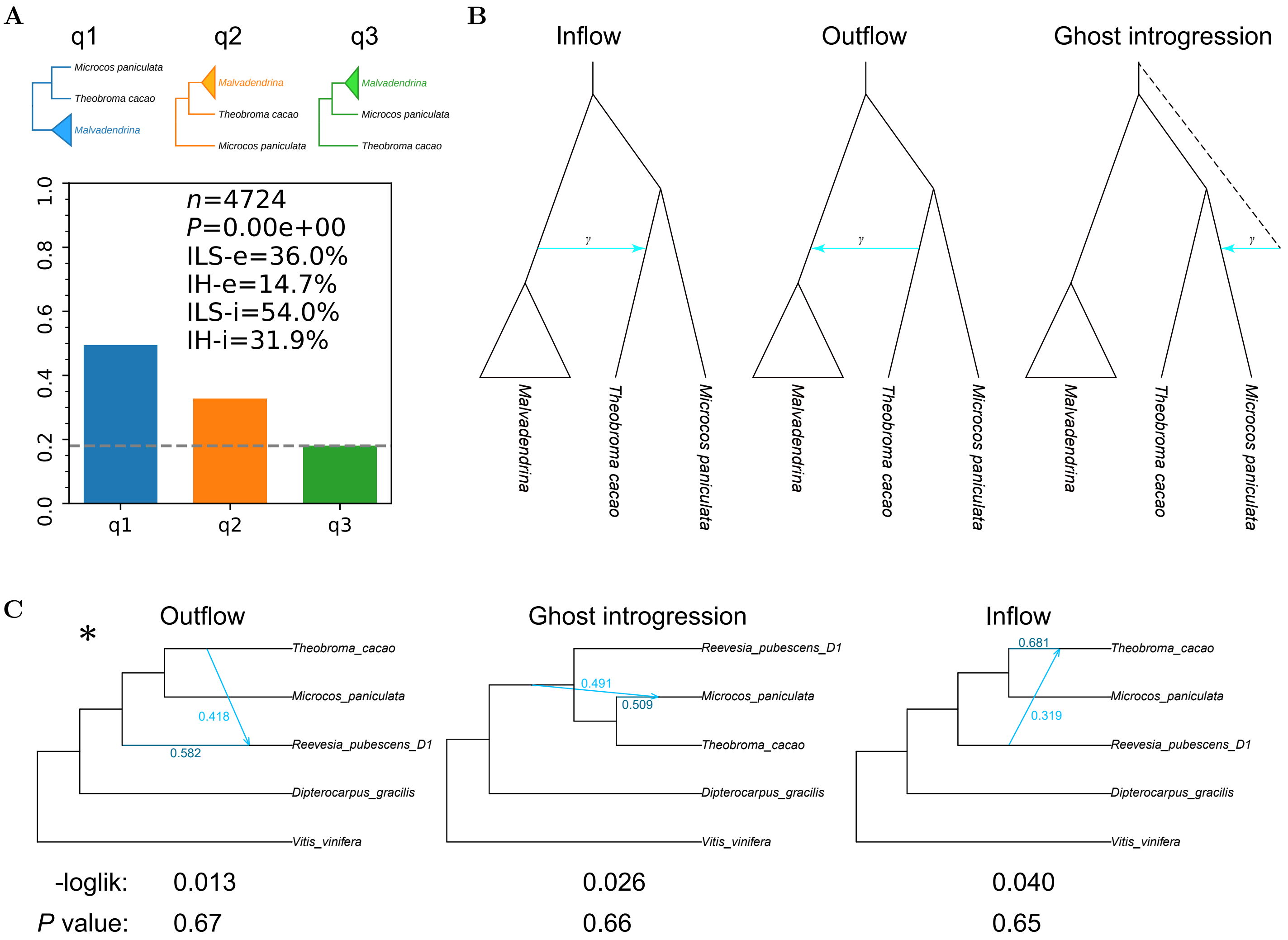

Supplementary Fig. 24. Introgression events during the first radiation of paleodiploids. (**A**) Patterns of gene tree discordance estimated by ASTRAL and visualized by PhyTop. (**B**) Three theoretical models proposed to explain the observed gene tree discordances. (**C**) Network reconstructions using SNaQ, complemented by pseudolikelihood evaluations and *P* values from the goodness-of-fit test. The asterisk (*) indicates the best model estimation. Source data are provided as a Source Data file.

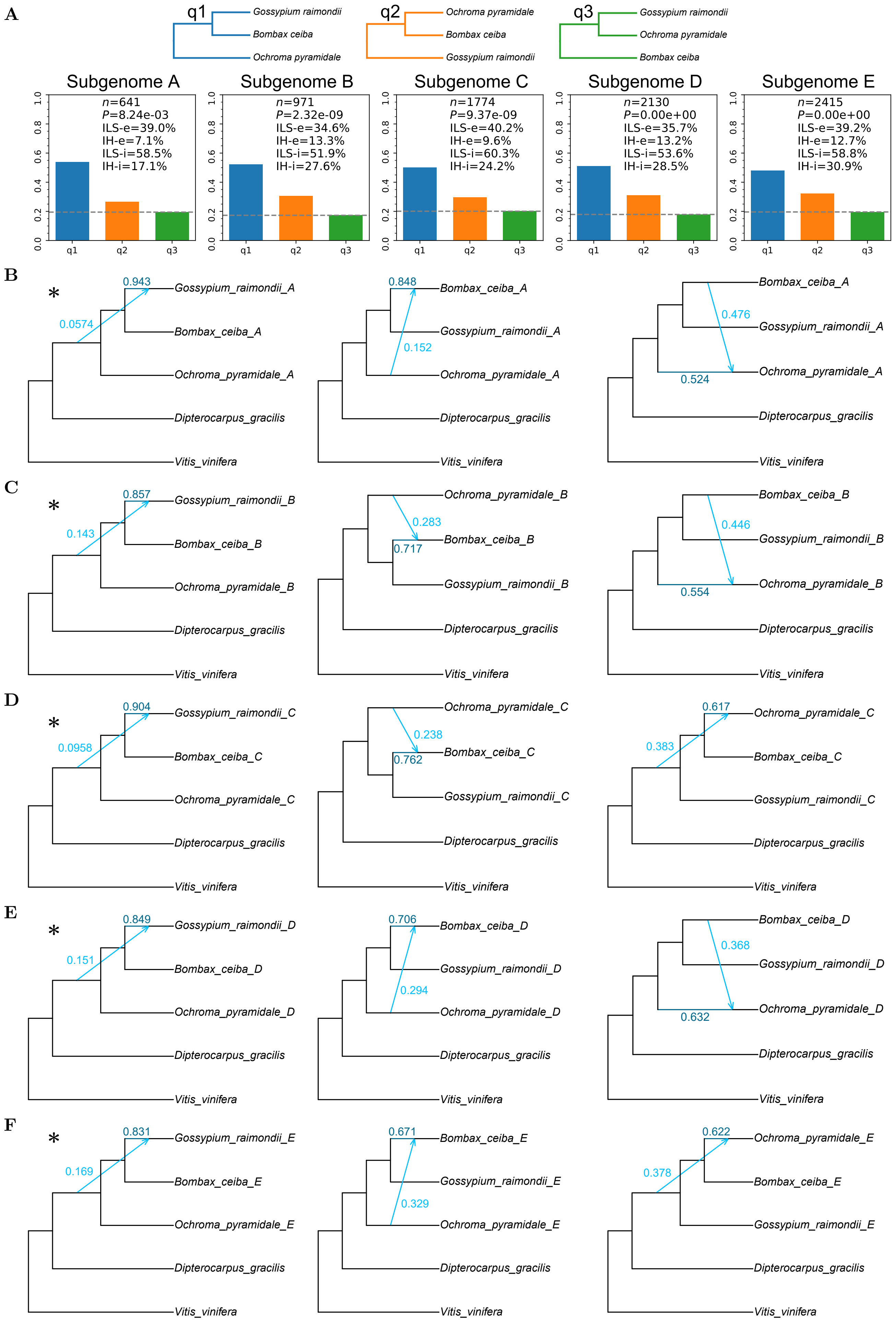

Supplementary Fig. 25. Detected introgression in the radiation of paleodecaploids. (**A**) Gene tree discordance patterns across five subgenomes. (**B**–**F**) Individual network reconstructions by SNaQ for each subgenome dataset. The asterisk (*) denotes the best model estimation. Source data are provided as a Source Data file.

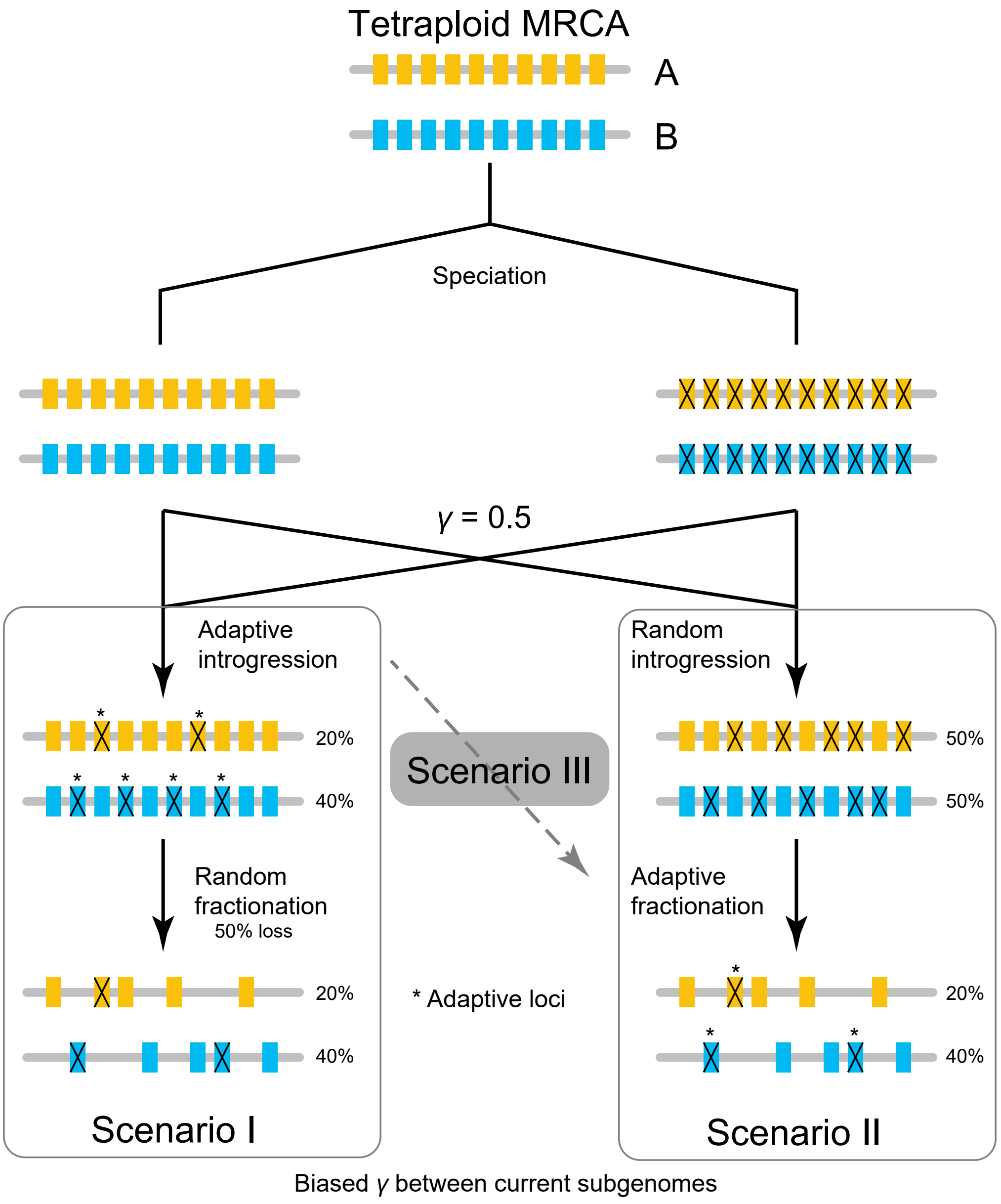

Supplementary Fig. 26. A hypothesized conceptual model elucidating the variance in observed inheritance probability (γ) between different subgenomes in paleoallopolyploids. Scenario I attributes biased γ to adaptive introgression, while scenario II associates it with adaptive fractionation and emphasizes the role of adaptation (or selection) of unknown temporal origin in these differences. Scenario III combines adaptive introgression and adaptive fractionation.

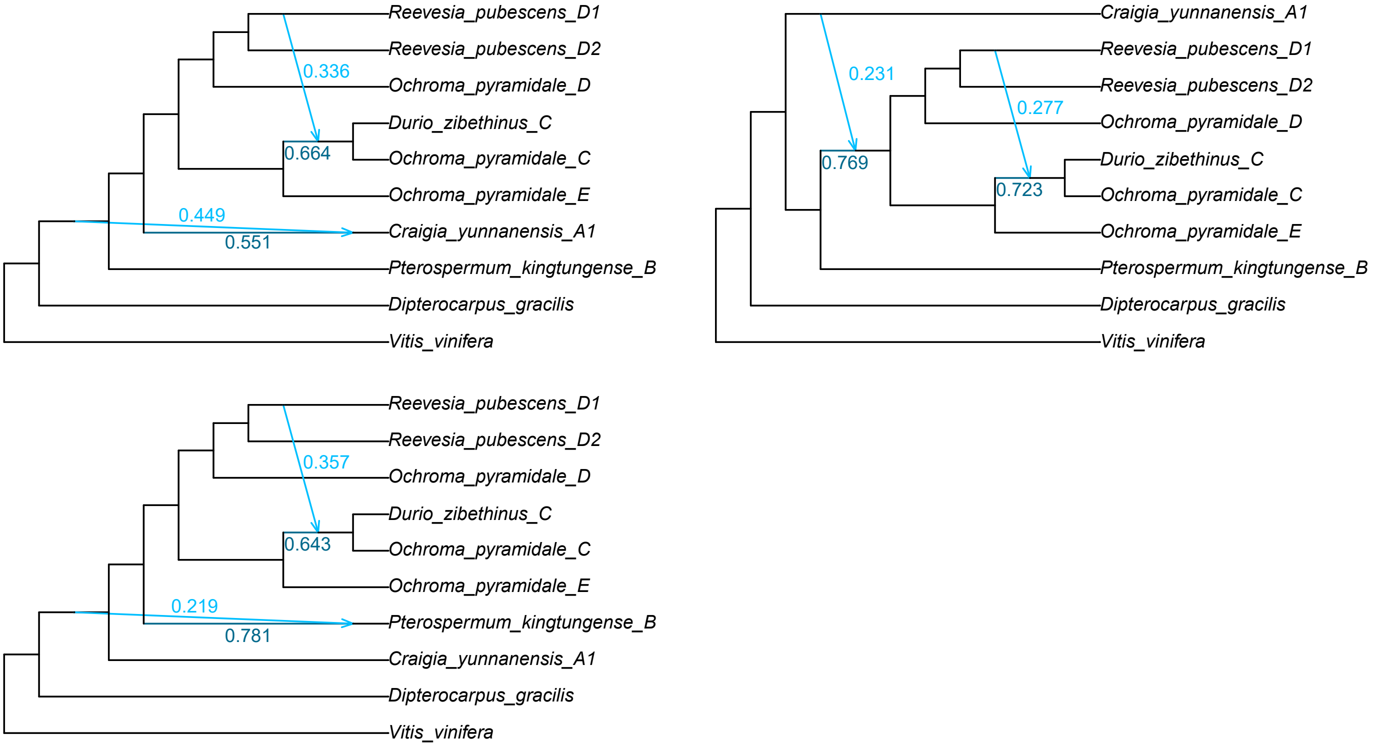

Supplementary Fig. 27. Phylogenetic networks representing the second radiation in paleodiploids reconstructed by SNaQ. The first network is considered the best estimate by pseudolikelihood evaluations. Each paleodiploid lineage is represented by a subgenome. Note that none networks passed the goodness-of-fit assessment. Source data are provided as a Source Data file.

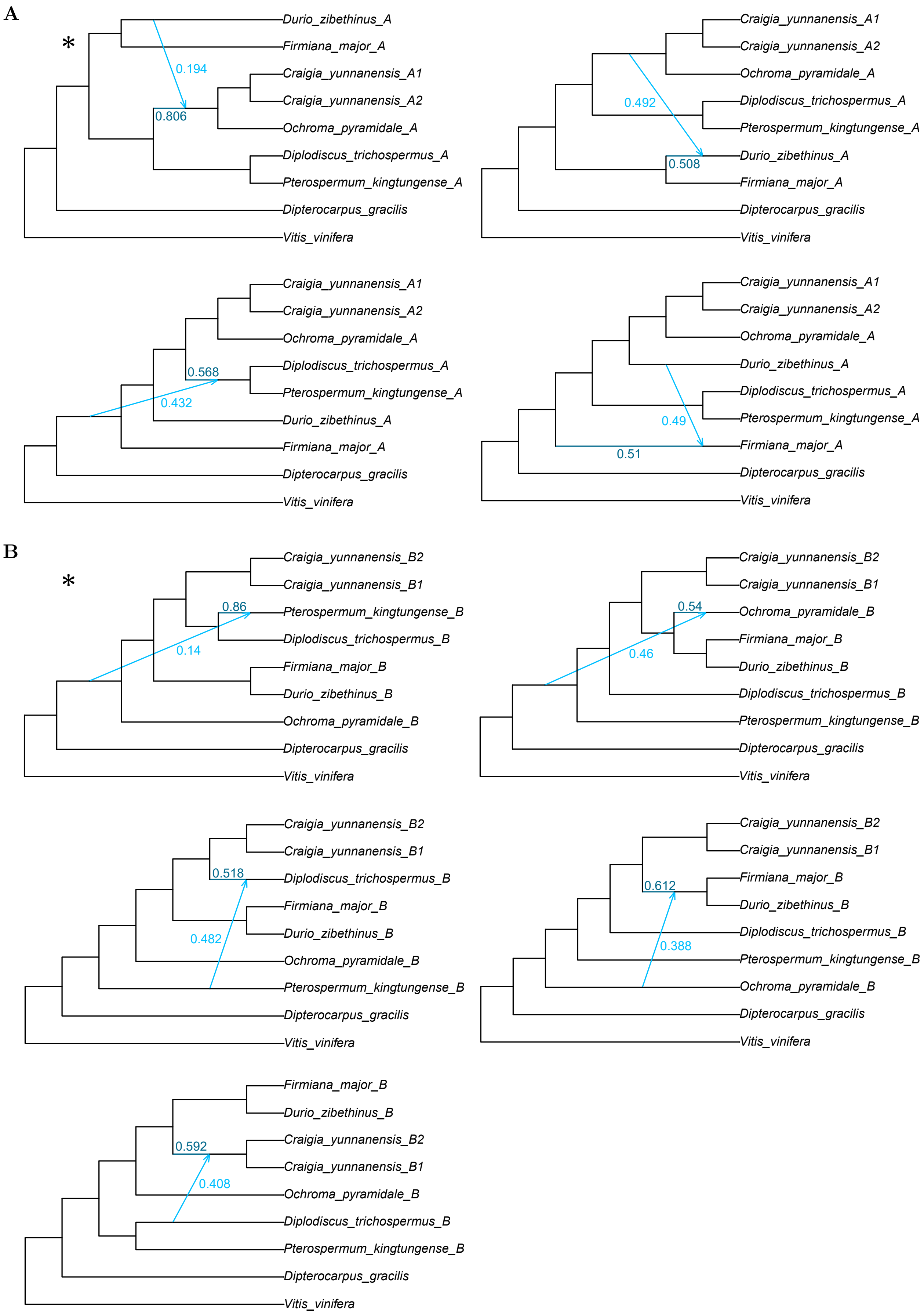

Supplementary Fig. 28. Phylogenetic networks reconstructed by SNaQ during the radiation of paleotetraploids. (A–B) Phylogenetic networks of A (**A**) and B (**B**) subgenomes. The asterisk (*) denotes the best estimated network by pseudolikelihood evaluations. The subgenomes of the Malvatheca clade were represented by the subgenomes of *Ochroma pyramidale*. Note that none networks passed the goodness-of-fit assessment. Source data are provided as a Source Data file.

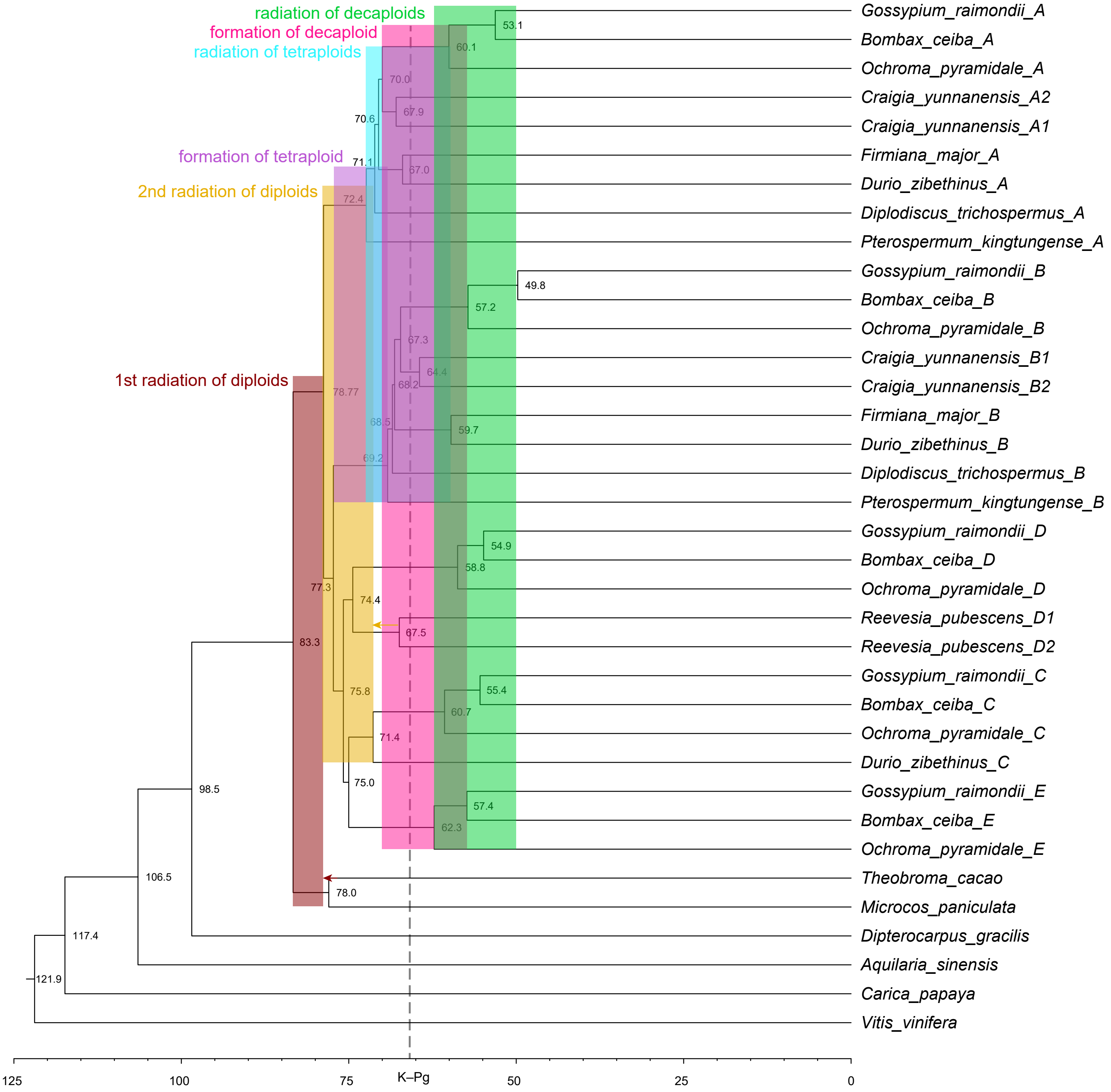

Supplementary Fig. 29. Divergence time estimation for subgenomes/species using MCMCTREE. Source data are provided as a Source Data file.

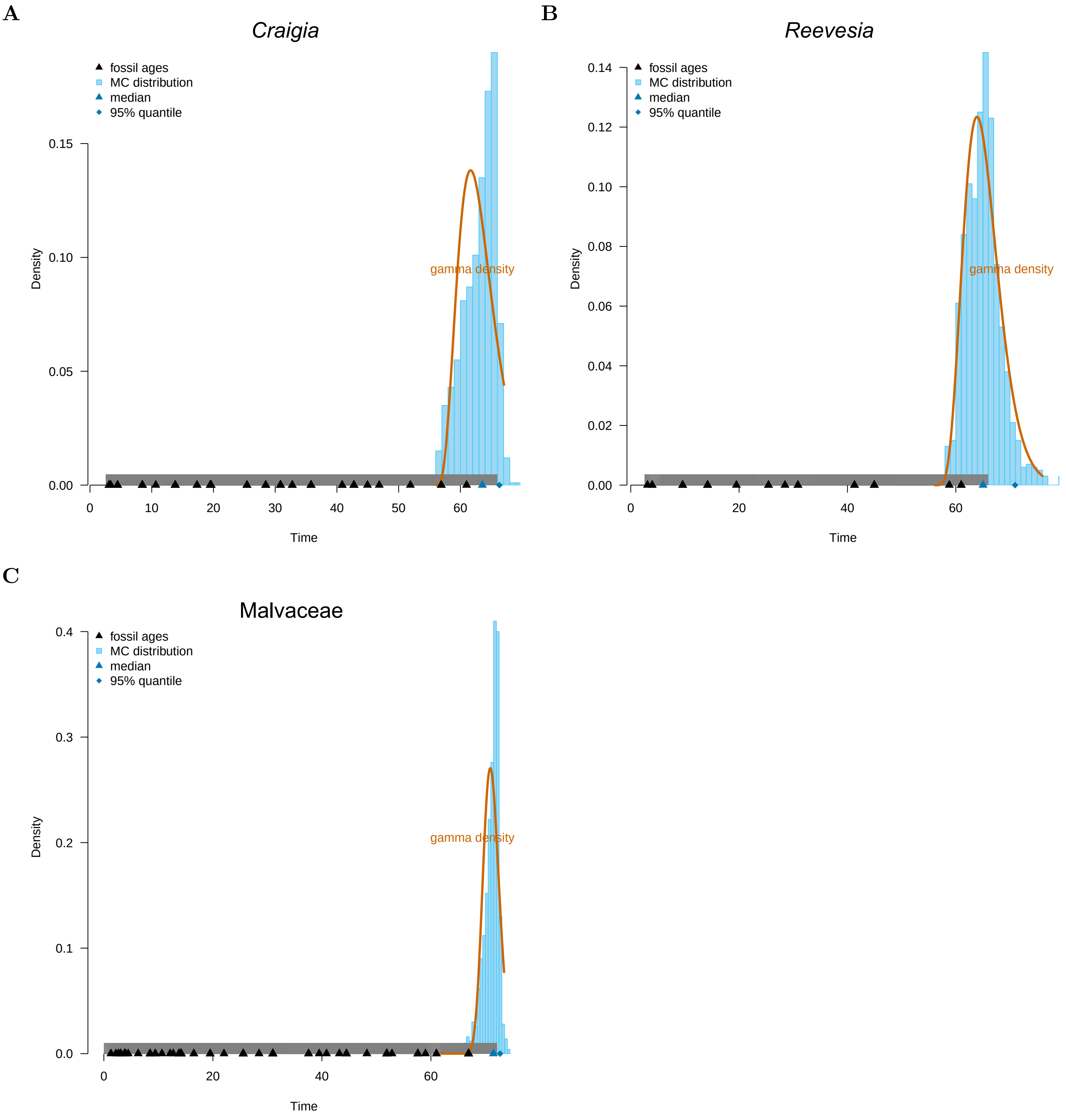

Supplementary Fig. 30. Dating of clade divergence based on fossil records of *Craigia*, *Reevesia* and Malvaceae by CladeDate. (**A**) Age distribution quantiles at 0%, 50% and 95% are 56.5, 63.6 and 66.3 Ma, respectively. (**B**) Age distribution quantiles at 0%, 50% and 95% are 57.3, 65.1 and 71.6 Ma, respectively. (**C**) Age distribution quantiles at 0%, 50% and 95% are 65.9, 71.4 and 72.9 Ma, respectively. The grey bars indicate the age intervals of the fossils, with the black triangles marking the midpoints. The sky-blue histograms show the age distributions by Monte Carlo resampling. The blue symbols represent the quantiles of these distributions, and the red line indicates a fit of a gamma probability density to the age distributions. Source data are provided as a Source Data file.

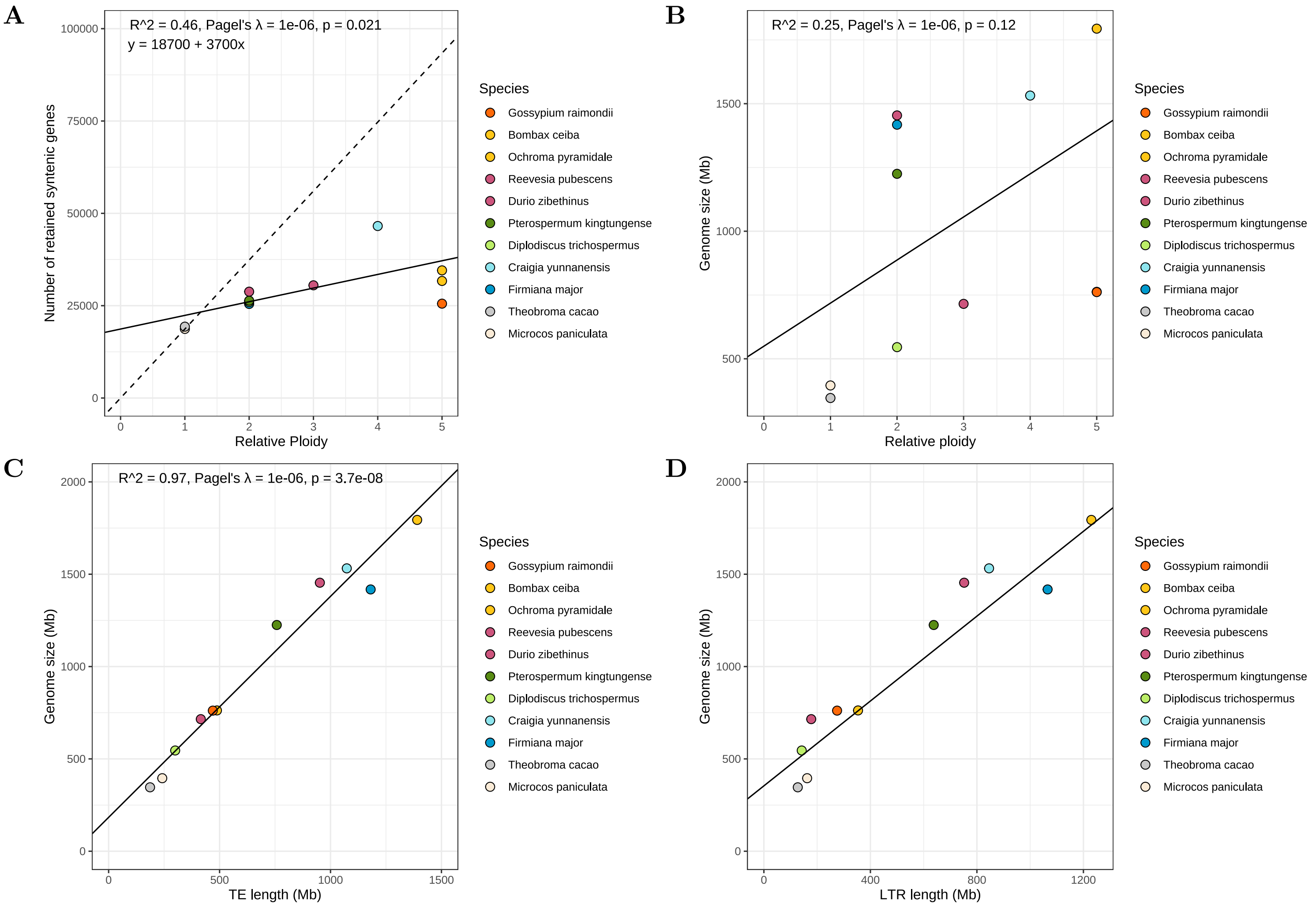

Supplementary Fig. 31. Relationships between the paleopolyploidiztions WGDs and different genomic features. (**A**) The correlation of relative ploidy to the number of retained syntenic genes. The dashed line indicates the expected gene numbers in case of no gene loss after paleopolyploidization, using cacao as the reference. (**B**) The correlation between relative ploidy and the genome size. (**C**) The correlation between the accumulated length of TEs and current genome size. (**D**) The correlation between the accumulated length of LTR-RTs and current genome size. Source data are provided as a Source Data file.

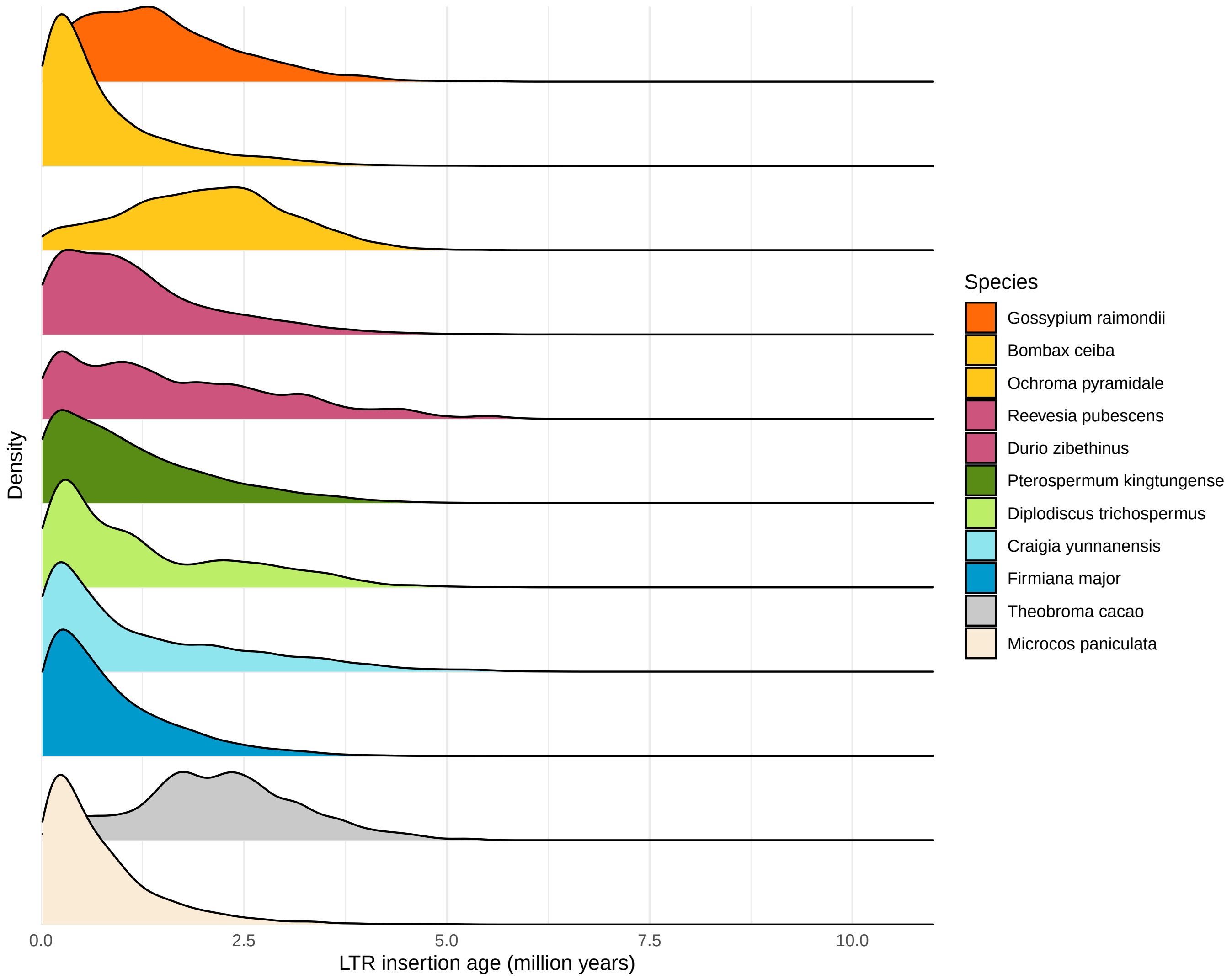

Supplementary Fig. 32. Insertion time estimates for LTR-RTs. Source data are provided as a Source Data file.

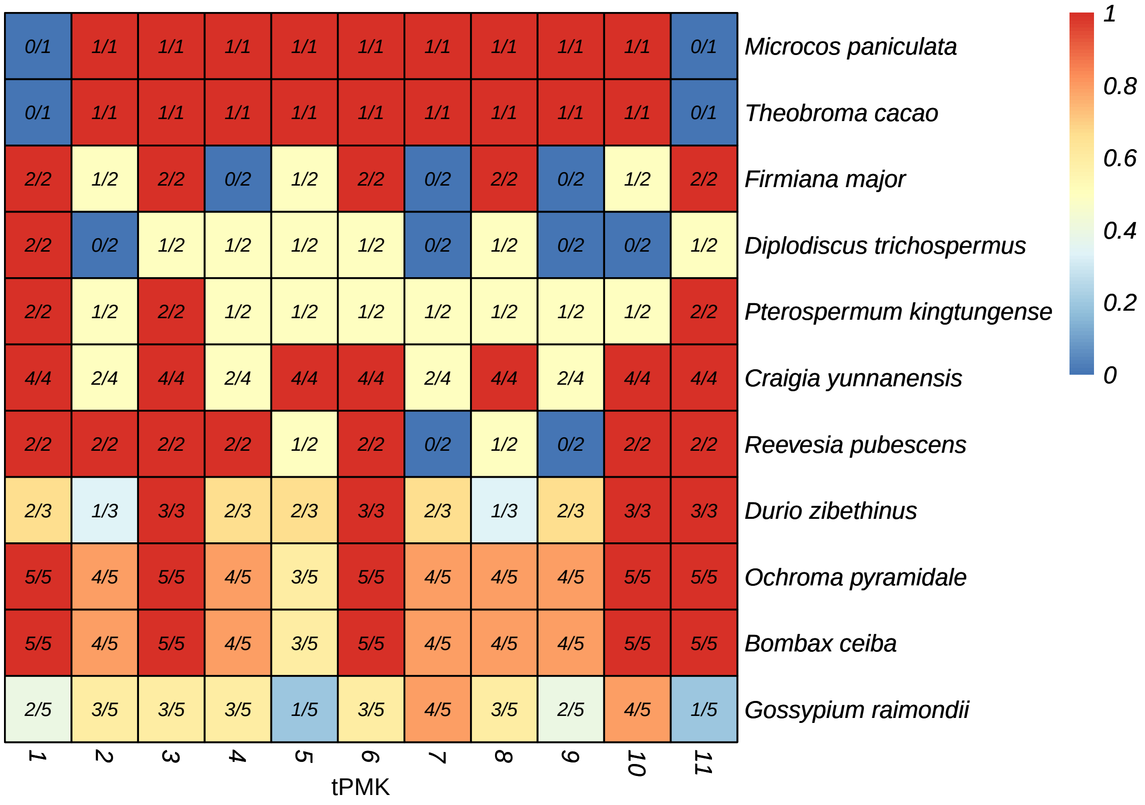

Supplementary Fig. 33. Summary of extant chromosomes that show intact chromosome-scale orthologous synteny to support the inference of tPMK. The number in each cell indicates the proportion of subgenomes in each genome supporting each tPMK chromosome.

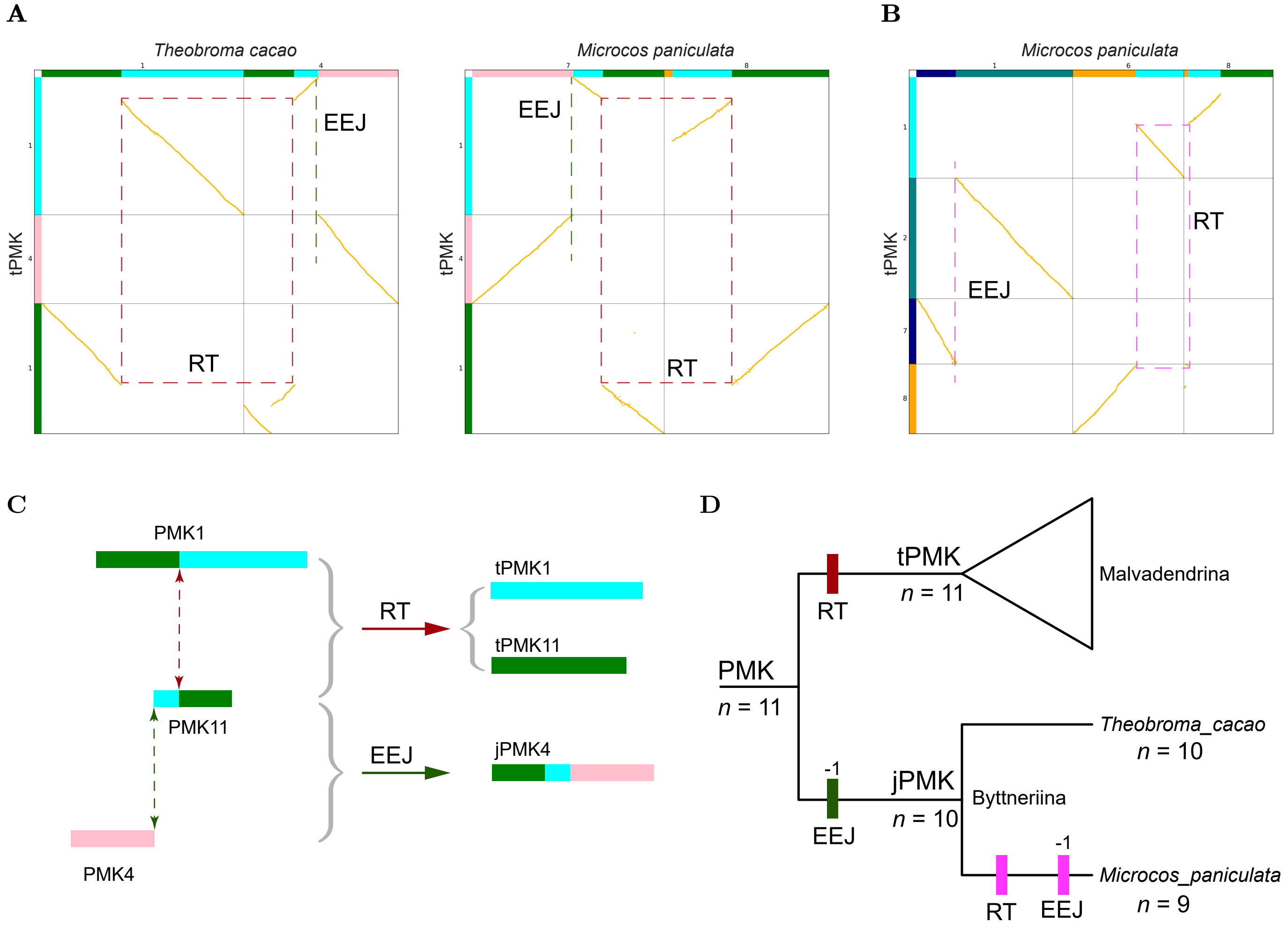

Supplementary Fig. 34. Insights into karyotype evolution in early diverging Malvaceae genomes. (**A**) Dot plots indicating two shared chromosomal rearrangements (CRs) in *Microcos paniculata* and *Theobroma cacao*, relative to tPMK. (**B**) Dot plots highlighting two lineage-specific CRs in *M. paniculata*. The chromosome breakpoints are marked by dashed squares or lines. A RT results in two breakpoints on both proto-chromosomes and extant chromosomes, while an EEJ leads to one breakpoint on an extant chromosome and no breakpoints on proto-chromosome. (**C**) A model illustrating the karyotype evolution from PMK to tPMK and jPMK. The structure of proto-chromosomes PMK4 and PMK11 was inferred based on chromosome-scale orthologous synteny between PMK/tPMK and outgroup genomes (**Supplementary Fig. 16**). (**D**) Proposed evolutionary trajectories. '-1' indicates a reduction of chromosome number (descending dysploidy) by one. Source data are provided as a Source Data file.

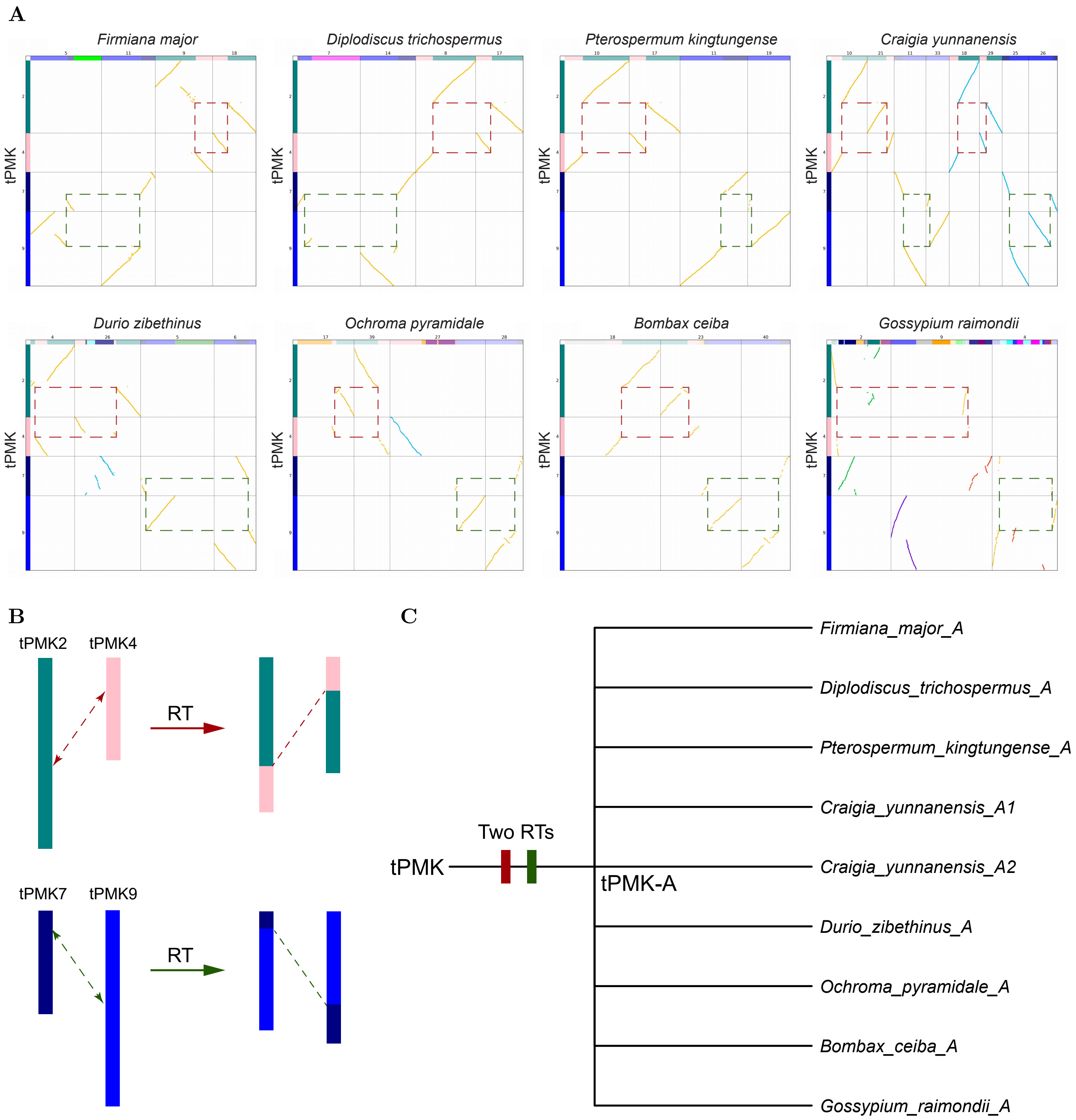

Supplementary Fig. 35. Characterization of inter-chromosomal rearrangements shared by all A-genome species. (**A**) Dot plots showing two CRs in all A-genome species, with breakpoints highlighted by dashed squares. (**B**) The origin of two reciprocal translocations (RTs) in A-genome species. (**C**) The two RTs in a phylogenetic context. Source data are provided as a Source Data file.

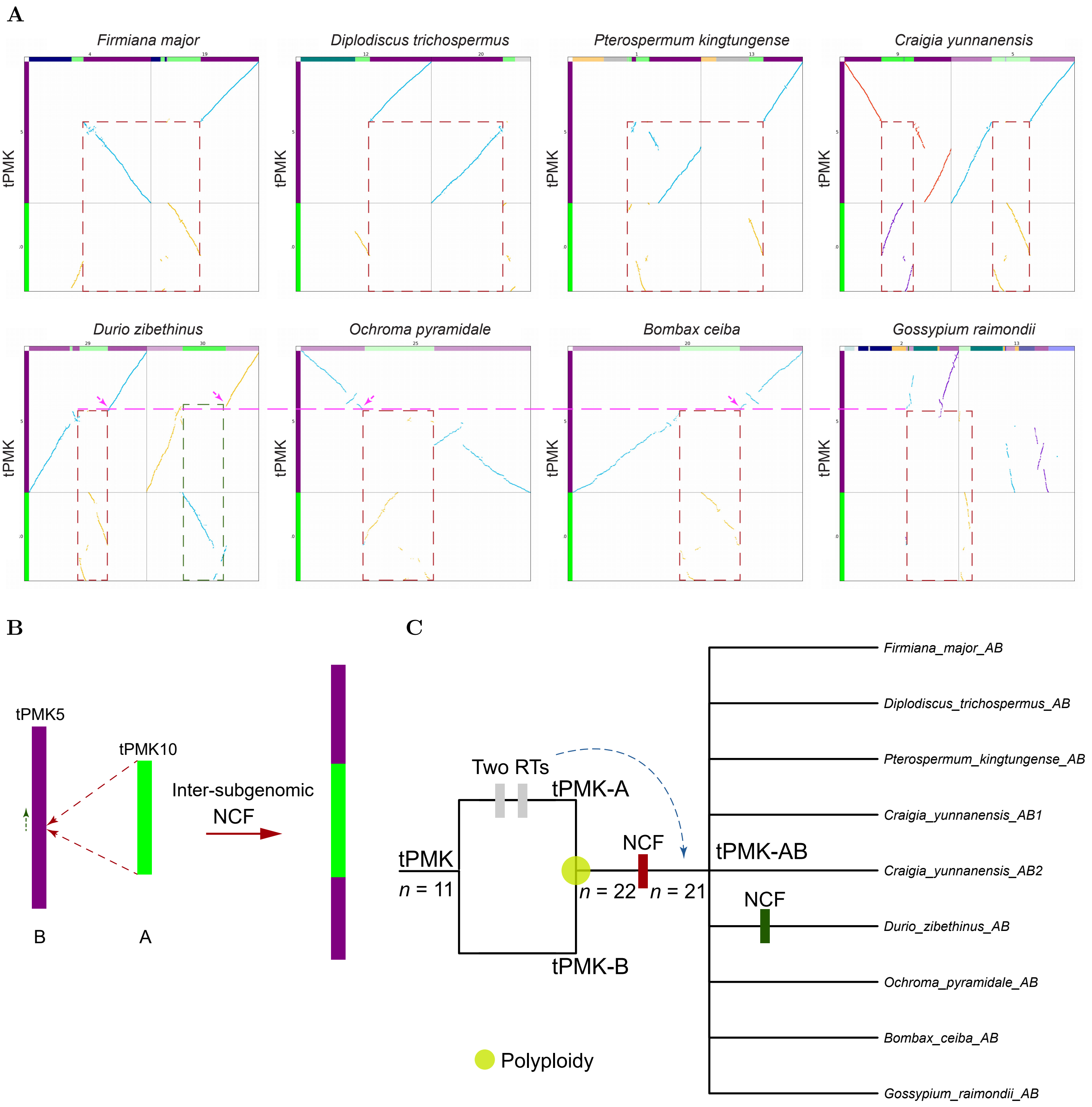

Supplementary Fig. 36. An inter-subgenomic chromosomal rearrangements (CRs) common to all AB subgenomes. (**A**) Dot plots illustrating shared CRs, with breakpoints highlighted by dashed squares. A NCF has one breakpoint on two different proto-chromosomes and two breakpoints on the extant chromosome. A dashed purple line indicates the difference between two similar NCFs in the subgenomes of *Durio zibethinus*. (**B**) A model of karyotype evolution detailing the NCFs. (**C**) Proposed evolutionary pathways. The dashed arrow indicates uncertainty about the position of two RTs in relation to the formation of the AB tetraploid genome. Source data are provided as a Source Data file.

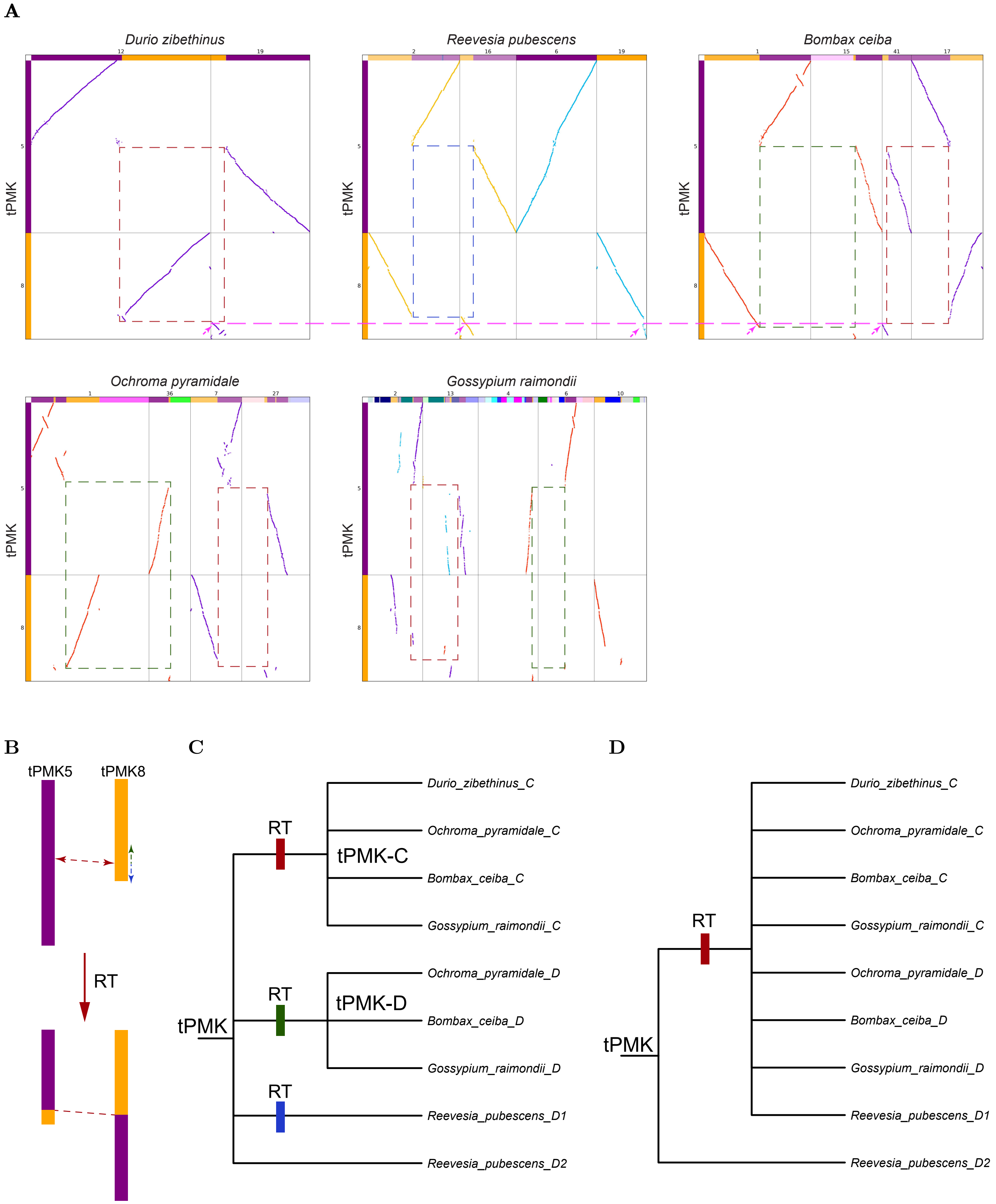

Supplementary Fig. 37. Common chromosomal rearrangements (CRs) identified in C and D subgenomes. (**A**) Dot plots displaying shared CRs. Dashed squares mark breakpoints of reciprocal translocations (RTs), and a dashed purple line and arrows highlight the differences between three similar RTs across lineages. (**B**) A model for the karyotype evolution through RT events. (**C**–**D**) Two alternative evolutionary trajectories, where the independent scenario (C) is supported by the differences between breakpoints (A). Source data are provided as a Source Data file.

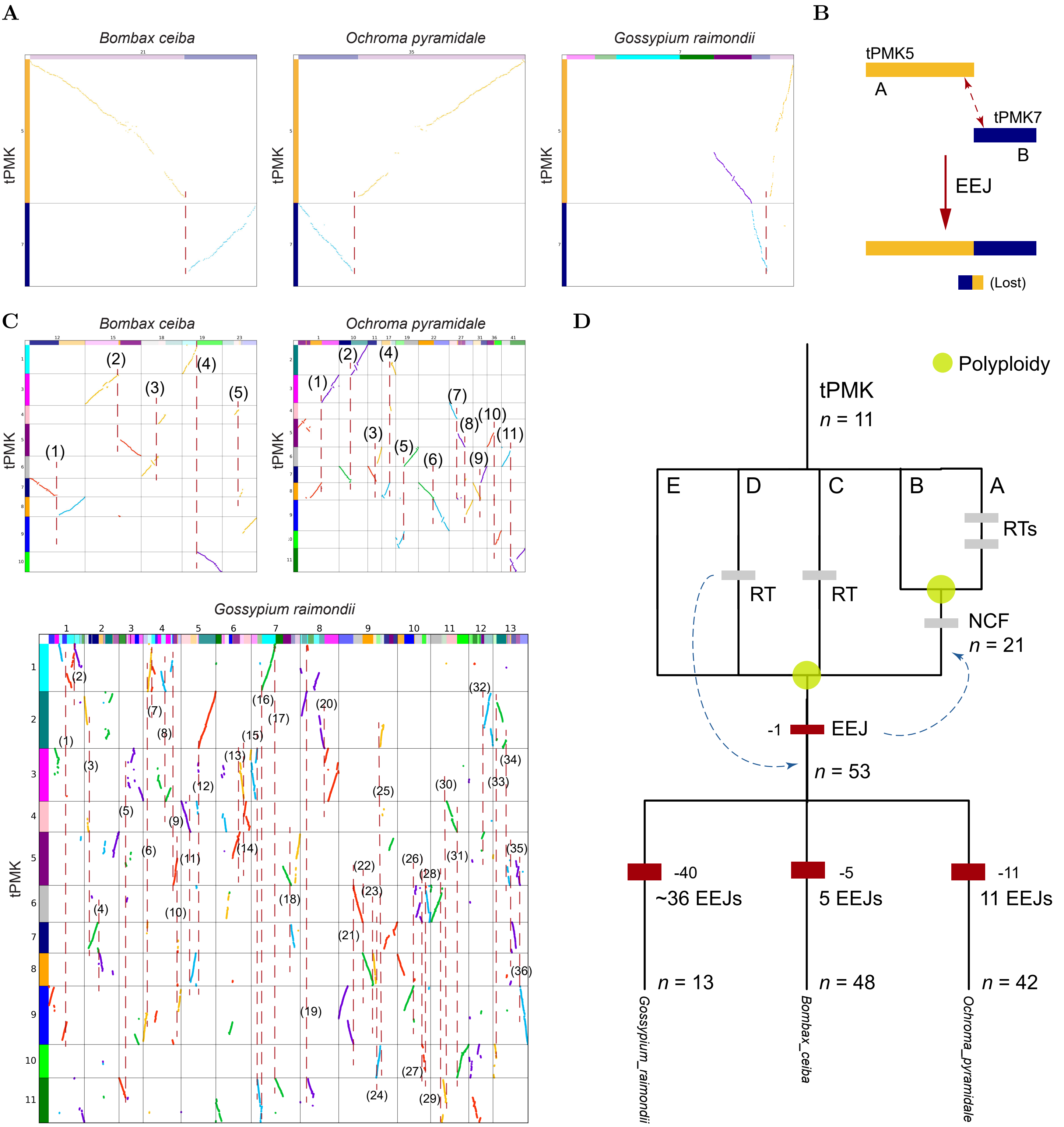

Supplementary Fig. 38. Karyotype evolution in the Malvatheca clade. (**A**) Dot plots reveal shared chromosomal rearrangements (CRs). The breakpoints are outlined by dashed lines. (**B**) The origin of tPMK5/7 fusion chromosome via an EEJ. (**C**) Dot plots showing species-specific CRs, with each breakpoint numbered sequentially. The number of CRs in the *G. raimondii* genome is likely underestimated due to the highly fragmented synteny. (**D**) Inferred evolutionary pathways. The dashed arrows indicate the uncertainty of the position of the CRs relative to the origin of the ABCDE decaploid genome. Source data are provided as a Source Data file.

A

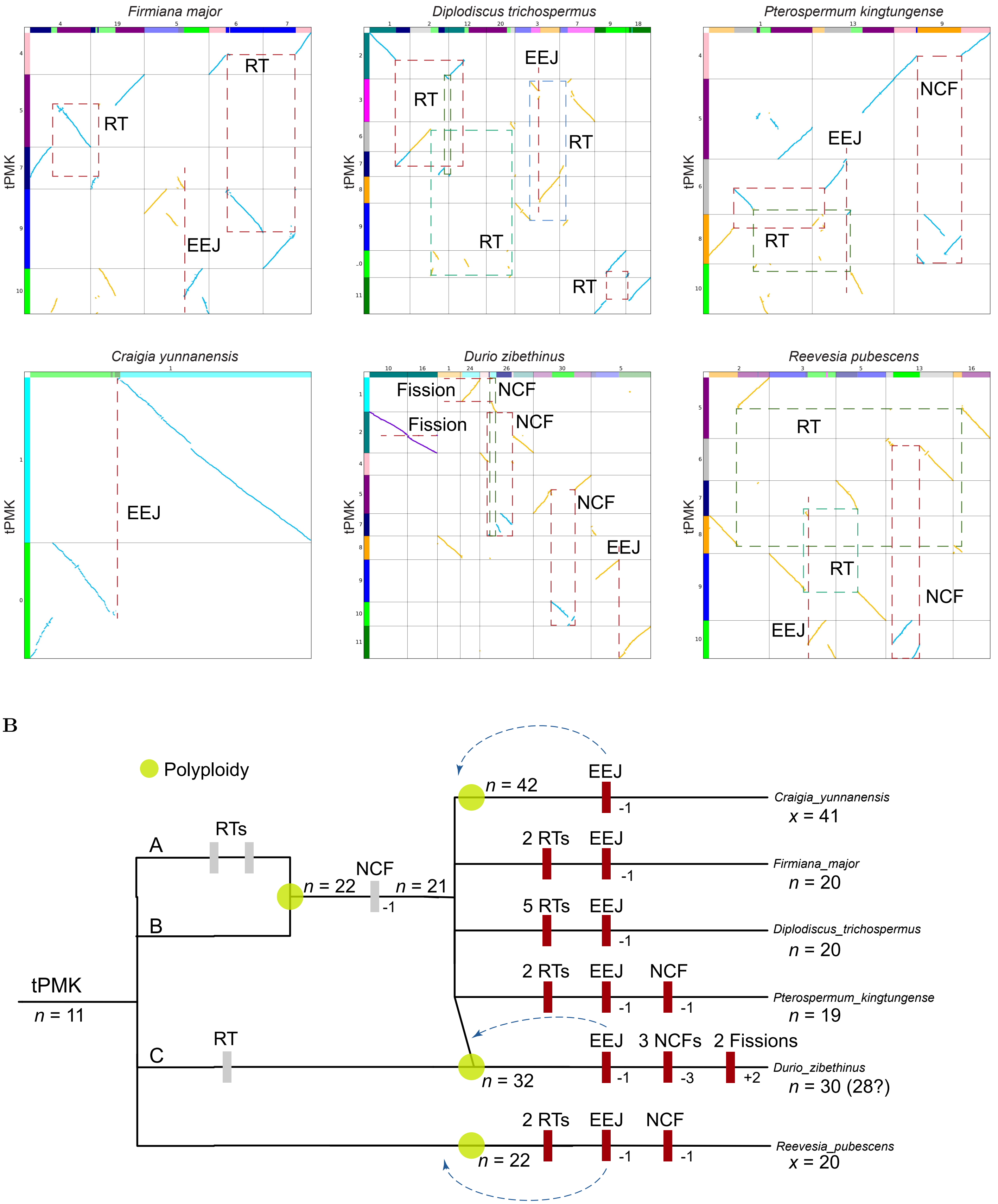

Supplementary Fig. 39. Species-specific chromosomal rearrangements (CRs) in non-Malvatheca genomes. (**A**) Dot plots showing unique CRs, with dashed squares or lines highlighting the breakpoints. (**B**) Proposed evolutionary trajectories based on CRs in (A). The dashed arrows indicate that some CRs may alternatively occur prior to the WGD. Source data are provided as a Source Data file.

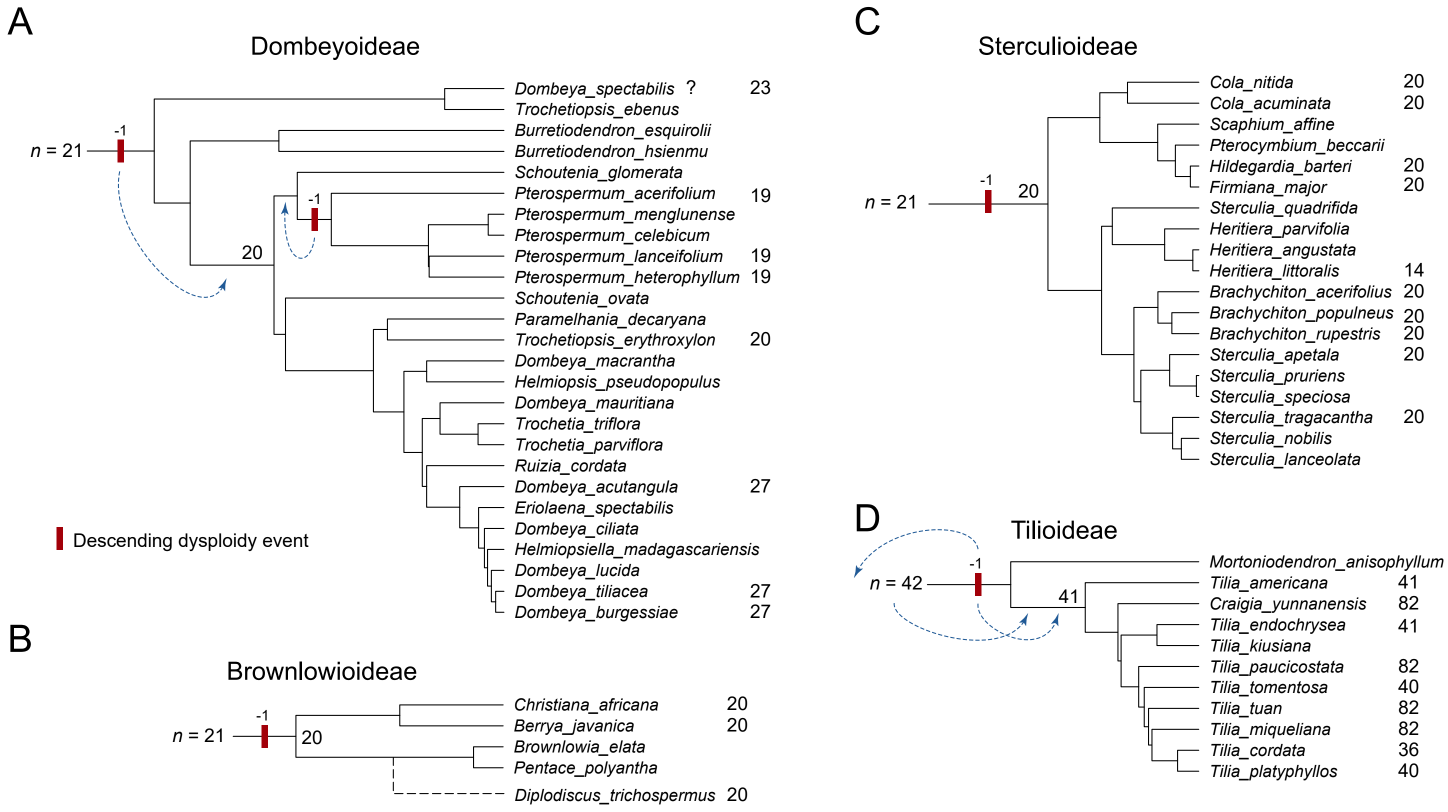

Supplementary Fig. 40. Inferred ancestral chromosome numbers and descending dysploidy events in crown groups of four subfamilies. (**A**–**D**) Descending dysploidy of the Dombeyoideae (**A**), Brownlowioideae (**B**), Sterculioideae (**C**) and Tilioideae (**D**). The phylogenetic trees and the modern chromosome numbers were adopted from PhytoPhylo[12] and the CCDB database[13], respectively. The dashed arrows suggest the uncertain placements due to lacking data for some basal taxa.

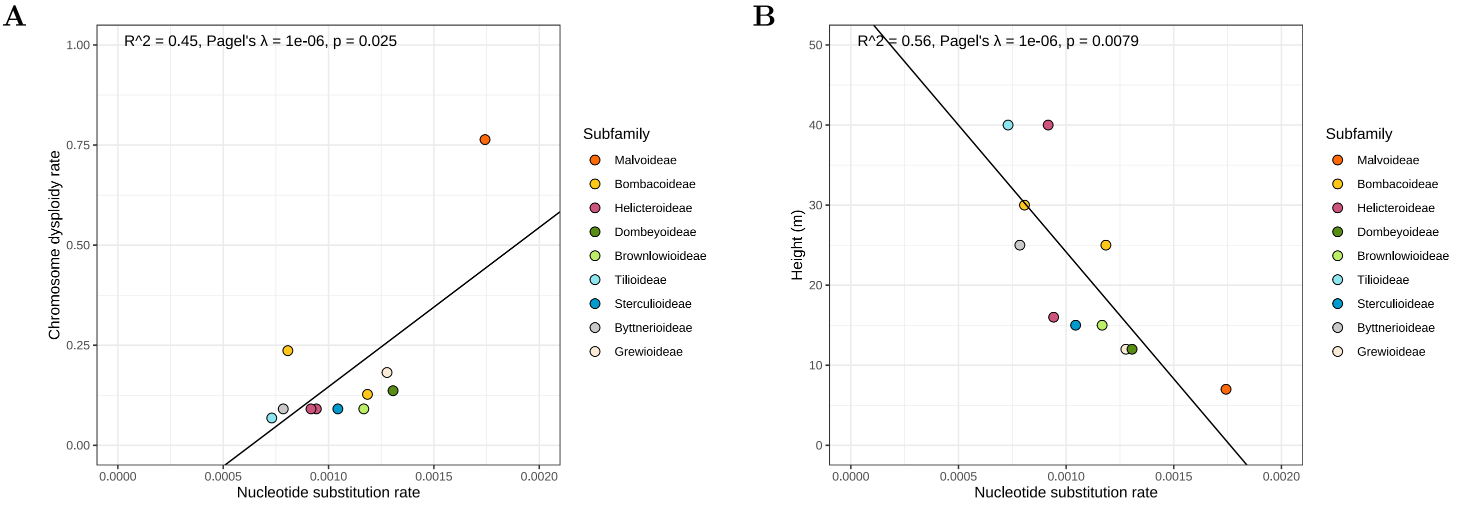

Supplementary Fig. 41. Association between degree of diploidization and nucleotide substitution rate. (**A**) Nucleotide substitution rate (substitutions per site per million years) versus dysploidy rate. (**B**) Nucleotide substitution rate (substitutions per site per million years) versus maximum plant height. Source data are provided as a Source Data file.

A

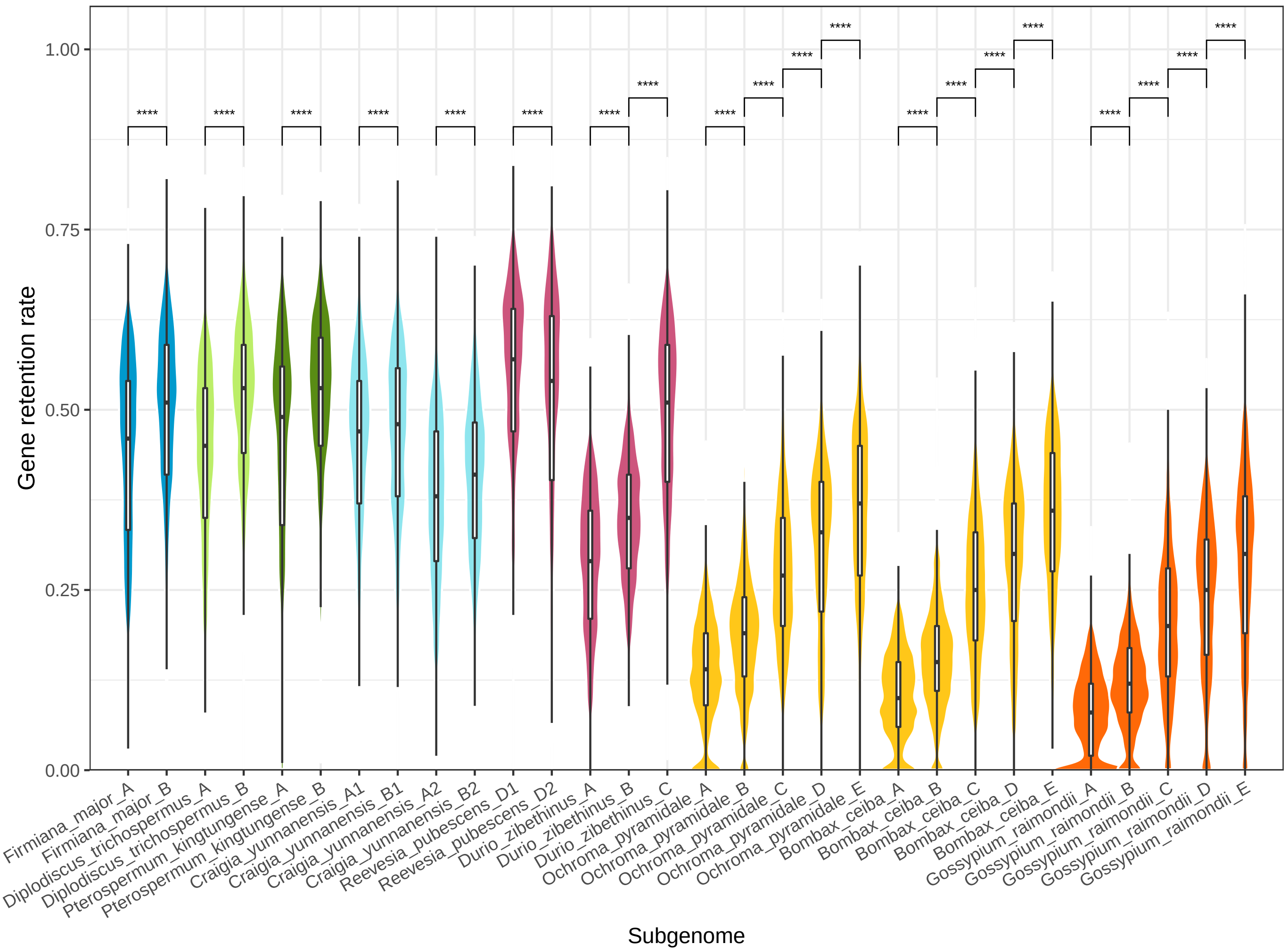

B

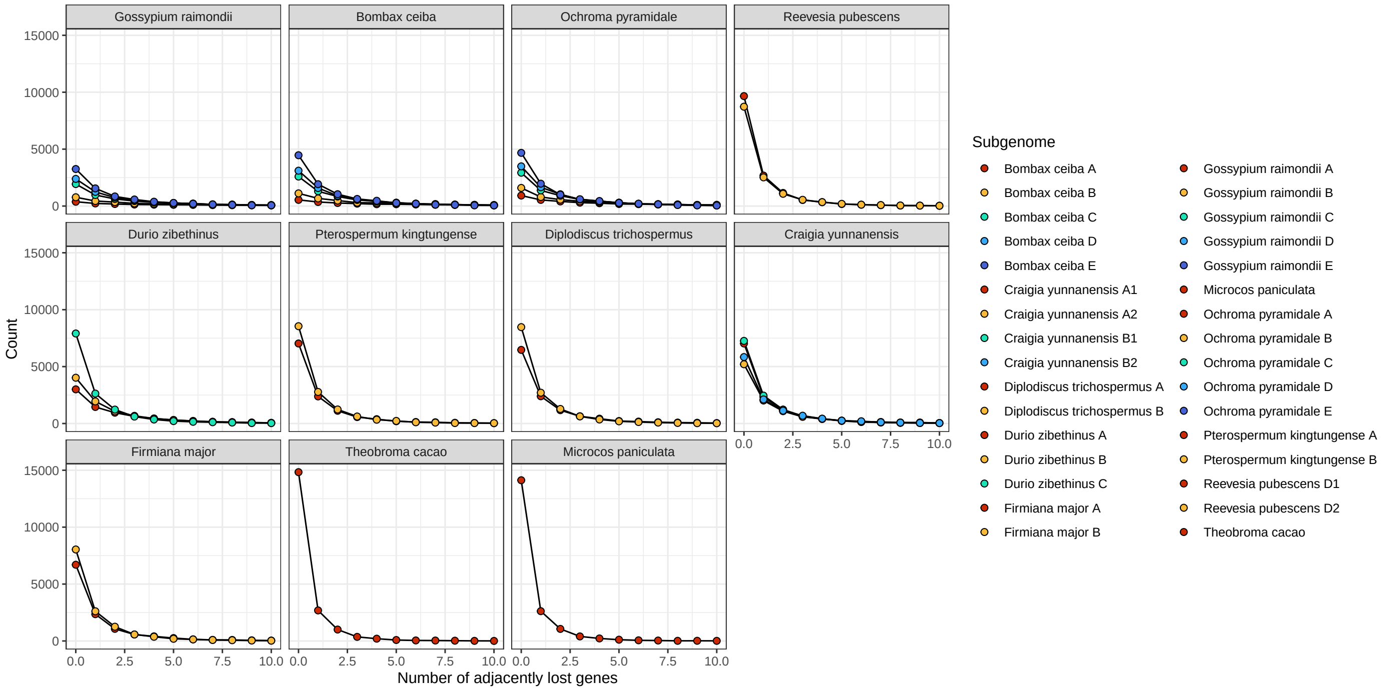

Supplementary Fig. 42. Patterns of gene retention or loss. (**A**) Gene retention rates across subgenomes by 100-gene sliding windows. **** $P <= 0.0001$; two-tailed, unpaired Wilcoxon rank-sum test. (**B**) The numbers of adjacently lost genes in each subgenome. Source data are provided as a Source Data file.

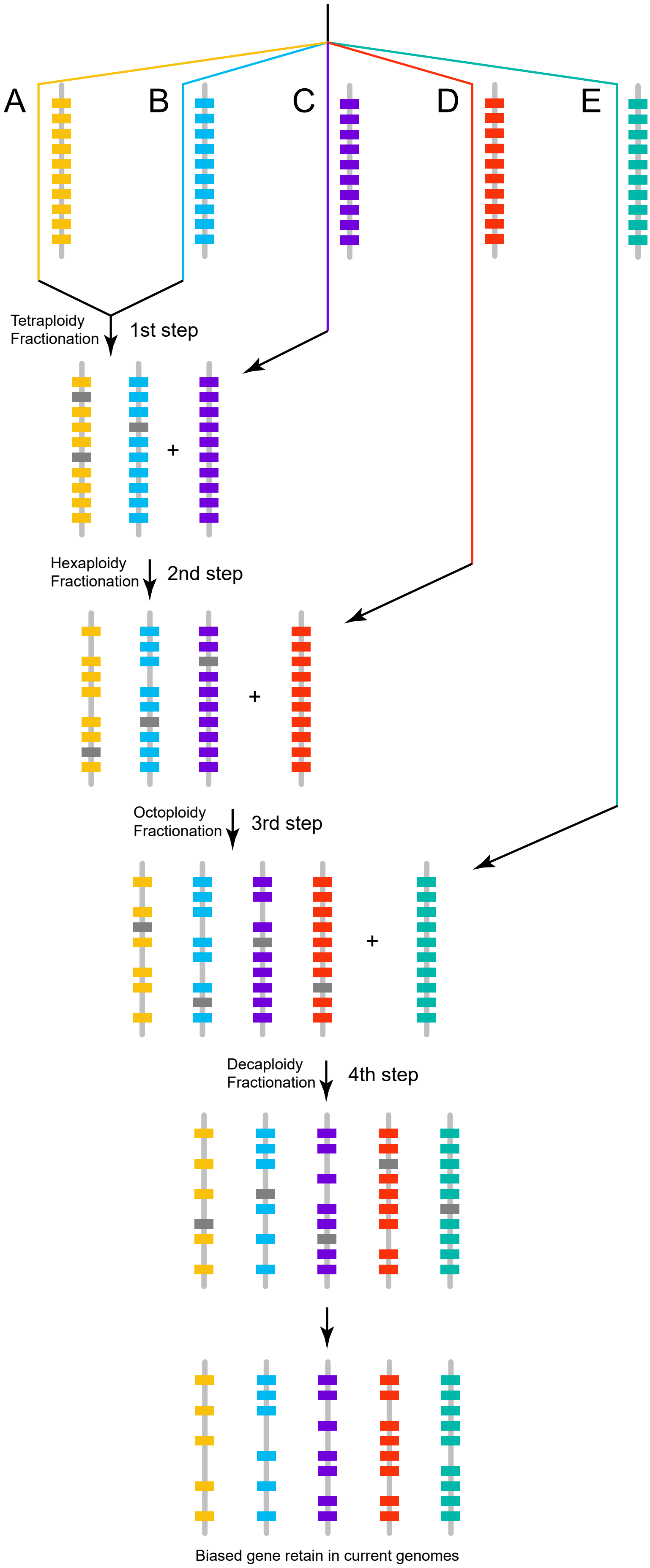

Supplementary Fig. 43. A hypothesized "four-step" model to elucidate the biased fractionation patterns (retention rate: A < B < C < D < E) observed in paleoallodecaploids, extending the previous two-step model.

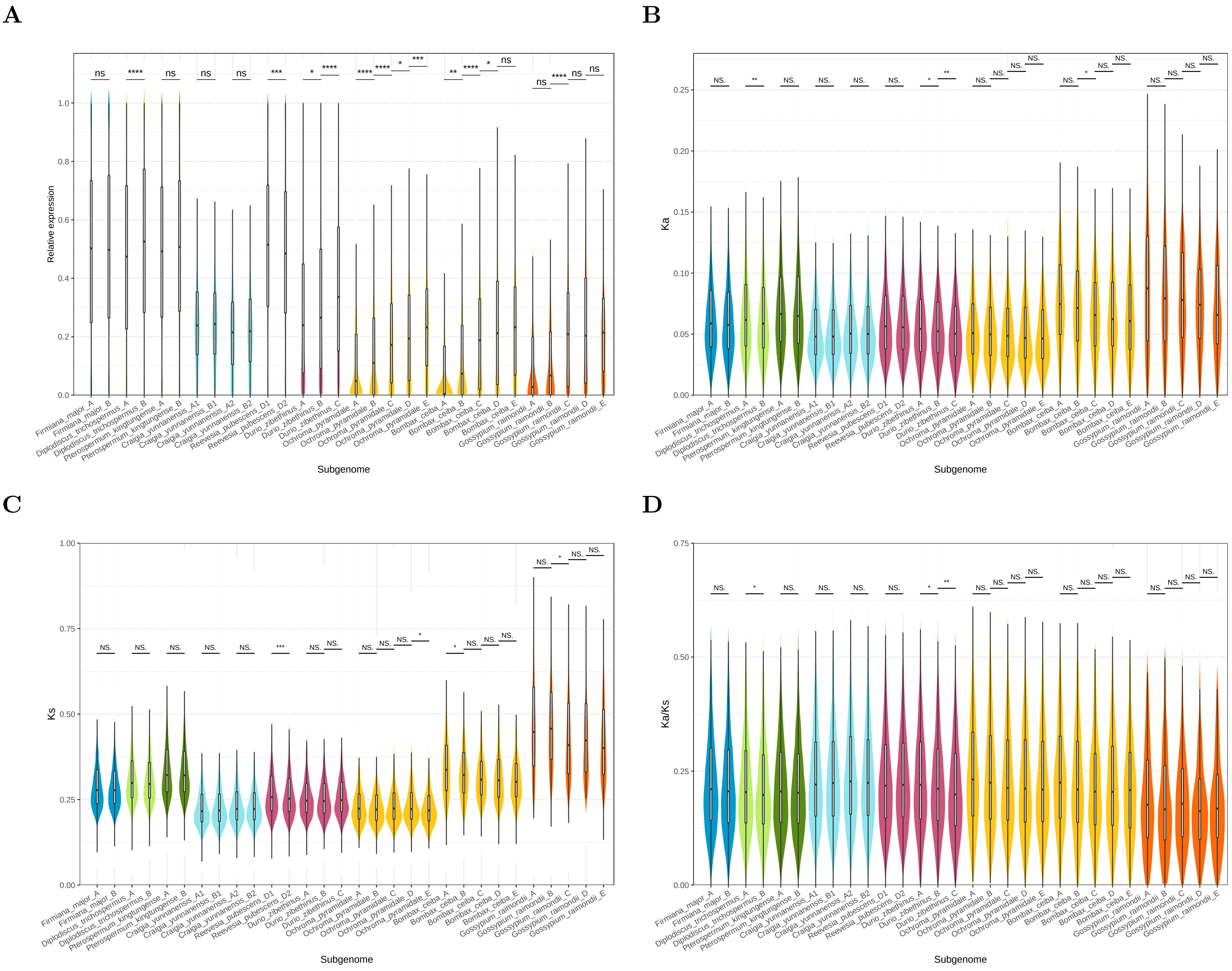

Supplementary Fig. 44. Comparison of subgenome homoeologs. (**A**–**D**) Comparison in terms of relative expression (**A**), *K*a (**B**), *K*s (**C**), and *K*a/*K*s (**D**). **** $P <= 0.0001$; *** $P <= 0.001$; ** $P <= 0.01$; * $P <= 0.05$; NS, $P > 0.05$; two-tailed, unpaired Wilcoxon rank-sum test. Source data are provided as a Source Data file.

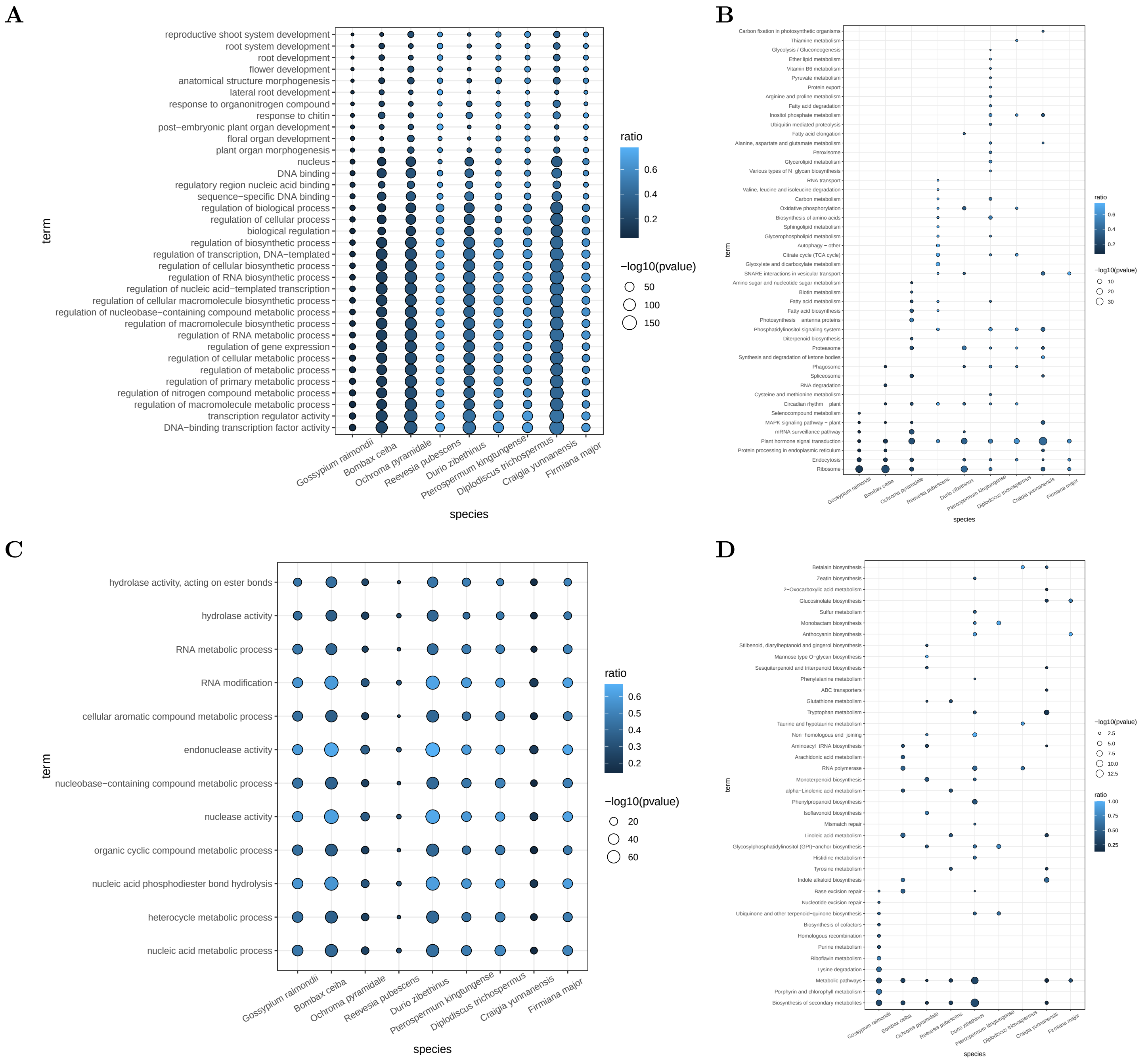

Supplementary Fig. 45. Function enrichment analyses for genes with high retention or loss rate. (**A**) GO enrichment for highly retained (at least 80% copies retained) genes. (**B**) KEGG pathway enrichment for highly retained (at least 80% copies retained) genes. (**C**) GO enrichment for single-copy genes. (**D**) KEGG pathway enrichment for single-copy genes. Only terms significantly enriched across all species were shown for GO enrichment. Source data are provided as a Source Data file.

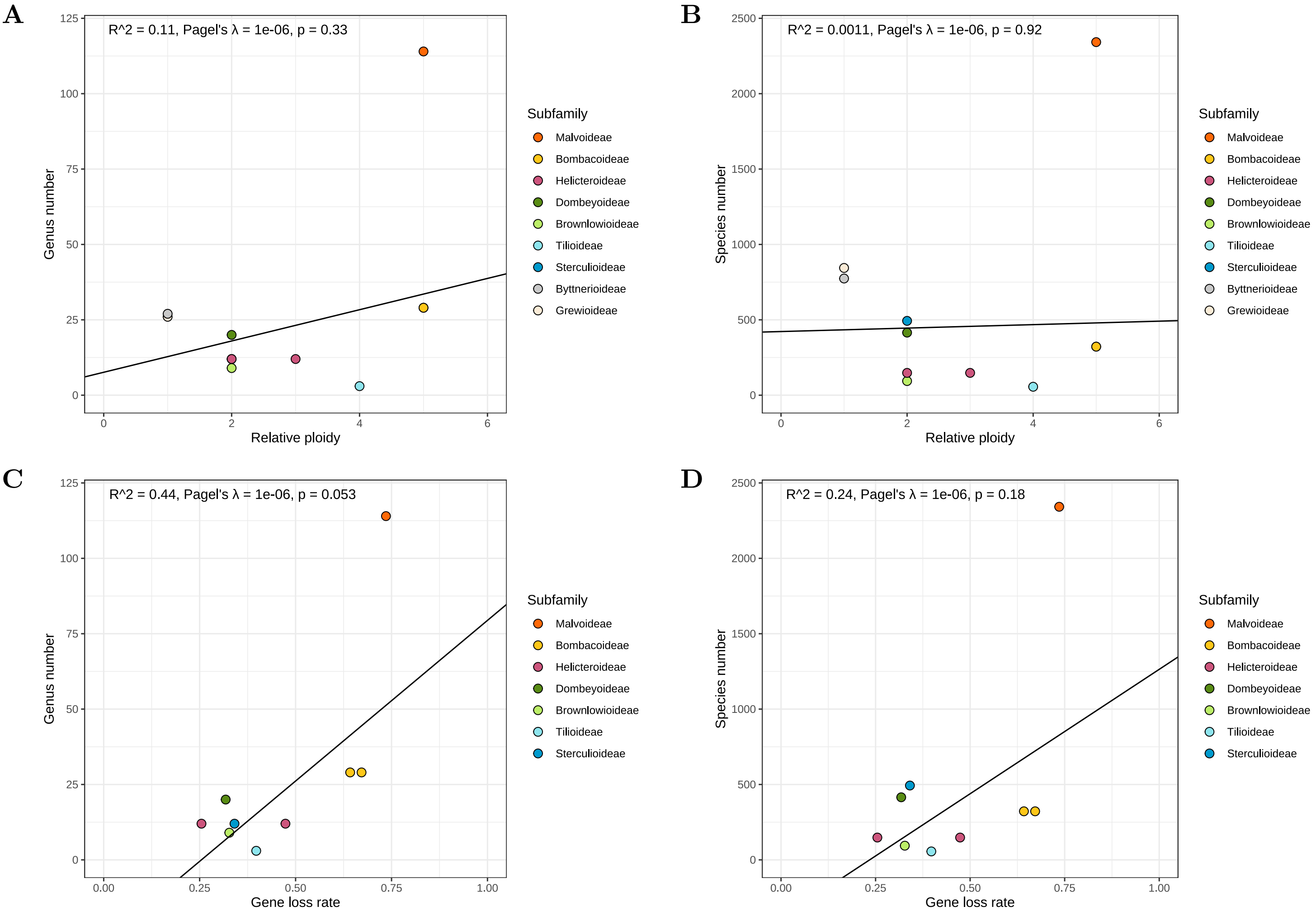

Supplementary Fig. 46. Correlations between genus/species richness and levels of polyploidization/diploidization. (A–B) Relative ploidy, reflecting the degree of polyploidization, plotted against the richness of genera (A) and species (B). (**C**–**D**) Gene loss rate quantifying the extent of PPD at the gene level (x-axis) plotted against the number of genera (C) and species (D) extracted from the World Flora Online (WFO) database. Source data are provided as a Source Data file.

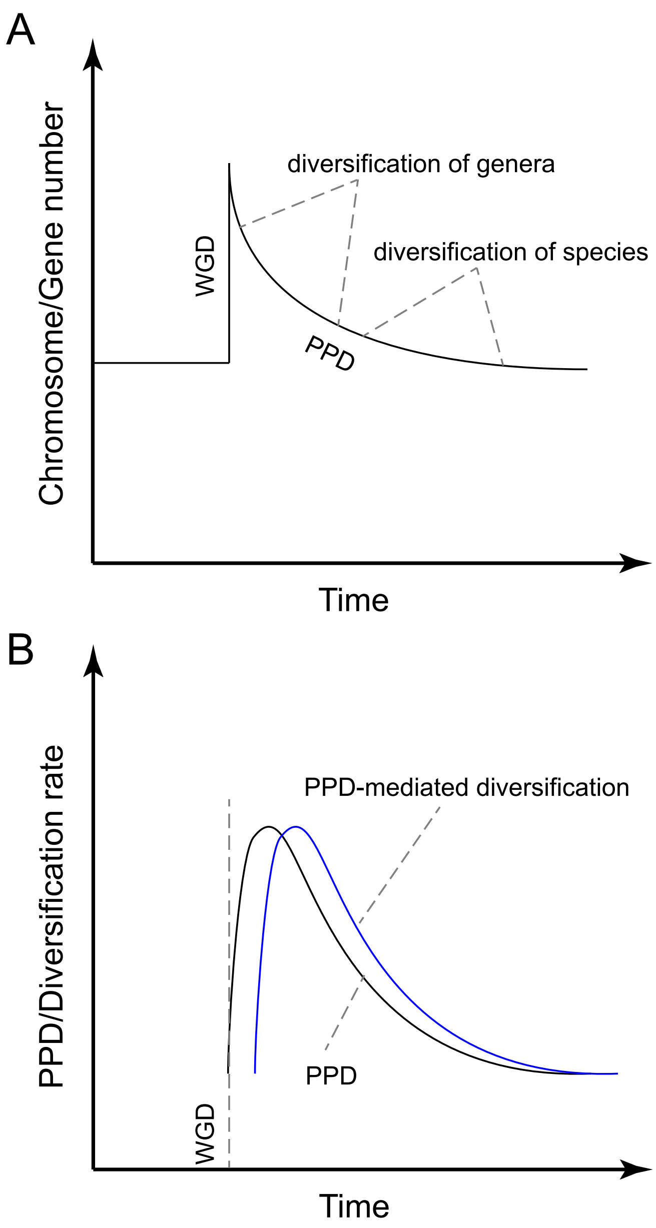

Supplementary Fig. 47. Schematic diagram to show a hypothesized model of PPD dynamics over time. (**A**) The model showing dysploidy rate or PPD rate gradually decreasing over time. (**B**) The model showing a lag time between dysploidy and diversification.

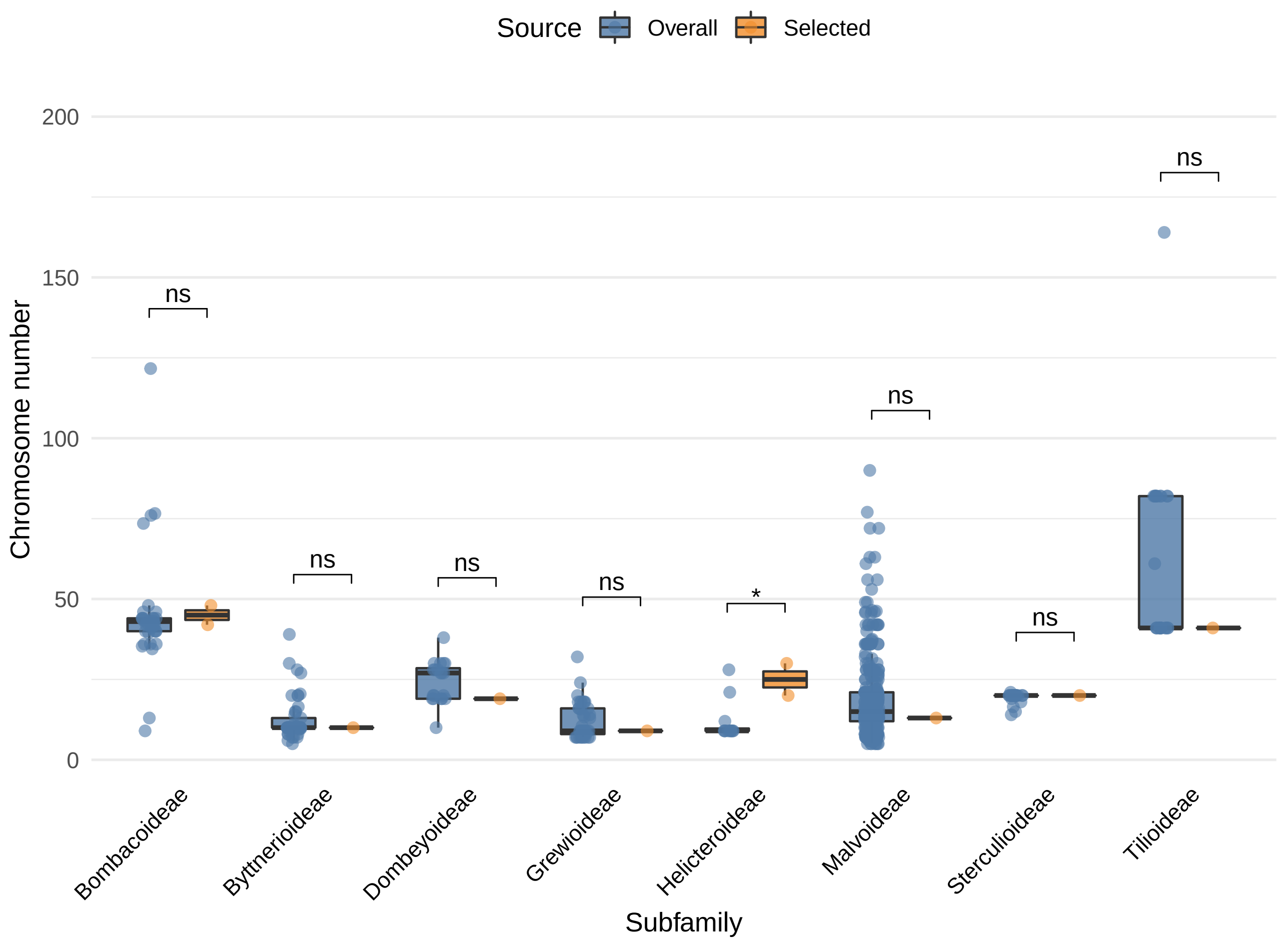

Supplementary Fig. 48. Comparison of chromosome counts of the selected species with those of the overall subfamily, based on data from literature[14]. *, $P <= 0.05$; ns, $P > 0.05$; two-tailed Wilcoxon rank-sum test.

Supplementary Table 1. Summary of genome sequencing data generated in this study.

Species	Tissue	Strategy	Platform	Read number (M)	Base number (Gb)	Read mean length (bp)	Read N50 length (bp)	Coverage depth (×)	SRA accession
Diplodiscus trichospermus	leaf	WGS	PacBio HiFi	2.24	37.26	16,606	16,431	68	SRR25905085
	leaf	WGS	BGISEQ	701.66	105.25	150	-	191	SRR25905084
	leaf	Hi-C	BGISEQ	551.19	82.68	150	-	150	SRR25905077
	mix	Iso-Seq	ONT	20.48	17.41	849	951	-	SRR25905076
Pterospermum kingtungense	leaf	WGS	PacBio HiFi	5.97	90.31	15,130	15,049	73	SRR25905075
	leaf	WGS	BGISEQ	642.55	96.38	150	-	78	SRR25905074
	leaf	Hi-C	BGISEQ	1079.29	161.89	150	-	132	SRR25905073
	mix	Iso-Seq	ONT	14.78	16.65	1,127	1,287	-	SRR25905072
Craigia yunnanensis	leaf	WGS	PacBio HiFi	6.14	98.49	16,051	15,884	64	SRR25905071
	leaf	WGS	BGISEQ	393.38	59.40	150	-	39	SRR25905070
	leaf	Hi-C	BGISEQ	2461.77	369.27	150	-	241	SRR25905083
	mix	Iso-Seq	ONT	21.59	20.59	953	1,095	-	SRR25905082
Reevesia pubescens	leaf	WGS	PacBio HiFi	9.71	192.88	19,854	19,680	133	SRR25905081
	leaf	WGS	BGISEQ	1025.53	153.83	150	-	106	SRR25905080
	leaf	Hi-C	BGISEQ	5770.64	865.60	150	-	597	SRR25905079
	mix	Iso-Seq	ONT	17.89	20.33	1,136	1,261	-	SRR25905078

Supplementary Table 2. Summary of nuclear genome assembly.

Assembly	Species	Genome size (Mb)	Chromosome number	Number of chromosome sets	GC content (%)	Contig number	Contig N50 (Mb)	Scaffold number	Scaffold N50 (Mb)	Gap number	BUSCO
Haplotype-resolved	*Diplodiscus trichospermus*	1091.2	40	2	32.4	48	26.8	43	27.1	5	C:98.9%[S:0.0%,D:98.9%],F:0.6%,M:0.5%,n:1614
	Pterospermum kingtungense	2449.9	38	2	34.6	43	64.9	40	65.0	3	C:99.2%[S:0.7%,D:98.5%],F:0.4%,M:0.4%,n:1614
	Craigia yunnanensis	6124.2	164	4	34.4	211	34.6	173	39.4	38	C:99.6%[S:0.3%,D:99.3%],F:0.2%,M:0.2%,n:1614
	Reevesia pubescens	8795.4	121	6	33.0	152	69.8	123	77.7	29	C:99.6%[S:0.3%,D:99.3%],F:0.2%,M:0.2%,n:1614
Primary monoploid	*Diplodiscus trichospermus*	545.5	20	1	32.4	22	26.8	20	27.1	2	C:98.9%[S:89.0%,D:9.9%],F:0.6%,M:0.5%,n:1614
	Pterospermum kingtungense	1225.0	19	1	34.6	20	64.9	19	65.0	1	C:99.1%[S:90.1%,D:9.0%],F:0.5%,M:0.4%,n:1614
	Craigia yunnanensis	1531.8	41	1	34.3	49	34.5	41	41.0	8	C:99.4%[S:41.8%,D:57.6%],F:0.3%,M:0.3%,n:1614
	Reevesia pubescens	1454.0	20	1	33.0	25	72.6	20	77.7	5	C:99.3%[S:67.3%,D:32.0%],F:0.3%,M:0.4%,n:1614

Note: the assemblies of primary monoploid were used for downstream analyses.

Supplementary Table 3. Summary of gene annotation for nuclear genome primary assembly.

Species	PCG number	mRNA number	tRNA number	rRNA number	other ncRNA number	BUSCO
Diplodiscus trichospermus	33,126	42,921	576	549	826	C:97.3%[S:88.9%,D:8.4%],F:1.4%,M:1.3%,n:1614
Pterospermum kingtungense	39,297	50,531	900	6,337	1,630	C:98.1%[S:90.3%,D:7.8%],F:1.1%,M:0.8%,n:1614
Craigia yunnanensis	57,219	69,869	796	213	3,029	C:97.6%[S:50.3%,D:47.3%],F:1.2%,M:1.2%,n:1614
Reevesia pubescens	39,788	47,670	688	400	1,787	C:97.2%[S:71.4%,D:25.8%],F:1.1%,M:1.7%,n:1614

Supplementary Table 4. Summary of TE annotation.

Order	Superfamily	*Diplodiscus trichospermus*			*Pterospermum kingtungense*			*Craigia yunnanensis*			*Reevesia pubescens*		
		Number	Length(bp)	Percent	Number	Length(bp)	Percent	Number	Length(bp)	Percent	Number	Length(bp)	Percent
LTR		196,230	142,048,340	26.04	836,414	637,814,241	52.06	1,158,209	845,685,624	55.21	851,918	752,084,148	51.72
	Copia	50,100	37,687,001	6.91	70,462	67,370,745	5.5	122,585	91,071,154	5.95	197,259	181,271,568	12.47
	Gypsy	64,687	64,323,239	11.79	312,589	284,189,047	23.2	467,987	418,563,368	27.33	258,276	268,594,064	18.47
	Retrovirus	330	169,240	0.03	78	91,572	0.01	158	203,520	0.01	-	-	-
	unknown	81,113	39,868,860	7.31	453,285	286,162,877	23.36	567,479	335,847,582	21.93	396,383	302,218,516	20.78
LINE		1,831	1,023,535	0.19	594	313,030	0.03	2,417	1,123,299	0.07	925	476,715	0.03
DNA		279,460	66,507,603	12.19	106,741	41,013,175	3.35	114,921	40,641,065	2.65	68,070	32,750,185	2.25
	Helitron	279,460	66,507,603	12.19	106,741	41,013,175	3.35	114,921	40,641,065	2.65	68,070	32,750,185	2.25
TIR		201,814	64,229,054	11.77	100,874	52,573,354	4.29	322,862	129,518,958	8.46	234,588	118,956,463	8.18
	EnSpm_CACTA	45,079	16,949,712	3.11	19,189	12,324,958	1.01	82,442	32,241,999	2.1	60,353	40,707,258	2.8
	MuDR_Mutator	94,616	25,572,957	4.69	46,756	23,141,954	1.89	147,030	61,927,514	4.04	136,005	56,445,366	3.88
	PIF_Harbinger	9,559	2,878,089	0.53	7,393	2,852,639	0.23	30,612	10,933,623	0.71	7,717	3,935,582	0.27
	Tc1_Mariner	9,235	2,278,314	0.42	3,295	922,313	0.08	15,501	4,313,627	0.28	5,371	3,598,377	0.25
	hAT	43,325	16,549,982	3.03	24,241	13,331,490	1.09	47,277	20,102,195	1.31	25,142	14,269,880	0.98
polinton		-	-	-	-	-	-	186	90,154	0.01	50	19,434	0
Unknown		56,908	18,392,039	3.37	17,893	9,683,484	0.79	136,040	39,155,628	2.56	63,092	27,069,171	1.86
Simple repeat		140,536	6,007,473	1.1	283,901	12,425,327	1.01	275,829	12,946,891	0.85	353,737	16,039,850	1.1
Low complexit		34,743	1,729,068	0.32	73,331	3,911,894	0.32	74,938	4,095,919	0.27	85,777	4,548,537	0.31
total		911,522	299,937,112	54.98	1,419,748	757,734,505	61.85	2,085,402	1,073,257,538	70.07	1,658,157	951,944,503	65.47

Supplementary Table 5. Summary of organelle genome assembly and annotation.

Source	Species	Genome size (bp)	GC content (%)	Contig number	PCG number	tRNA number	rRNA number
Plastid	*Diplodiscus trichospermus*	158,658	37.2	1	78	25	4
	Pterospermum kingtungense	162,016	36.5	1	79	25	4
	Craigia yunnanensis	162,586	36.5	1	79	25	4
	Reevesia pubescens	161,789	36.8	1	78	25	4
	Durio zibethinus	143,046	34.5	1	74	25	4
	Ochroma pyramidale	161,376	36.8	1	79	25	4
Mitochondrion	*Diplodiscus trichospermus*	651,338	45.0	2	37	19	3
	Pterospermum kingtungense	532,574	44.8	1	37	20	3
	Craigia yunnanensis	620,710	45.2	8	36	18	3
	Reevesia pubescens	825,793	44.7	1	37	18	3
	Durio zibethinus	2,235,832	44.6	3	35	21	3
	Ochroma pyramidale	632,995	45.0	3	37	20	3

Supplementary Table 6. Summary of monoploid genome assemblies of 11 Malvaceae species analyzed.

Species	Subfamily	Genome size (Mb)**	Chromosome number (*x*)**	Scaffold N50 (Mb)	Genome BUSCO (%)	PCG number	PCG BUSCO (%)	Relative ploidy (*p*)	Source
Microcos paniculata	Grewioideae	395	9	45.6	98.5	24,820	97.6	1	ref.[15]
Theobroma cacao (cocao)	Byttnerioideae	346	10	34.4	98.3	29,452	99.4	1	ref.[16]
Firmiana major	Sterculioideae	1 420	20	80.3	98.7	31,965	98.2	2	ref.[17]
Diplodiscus trichospermus	Brownlowioideae	545	20	27.1	98.9	33,126	97.3	2	this study
Pterospermum kingtungense	Dombeyoideae	1 230	19	65.0	99.1	39,297	98.1	2	this study
Craigia yunnanensis *	Tilioideae	1 530	41	41.0	99.4	57,219	97.6	4	this study
Reevesia pubescens *	Helicteroideae	1 450	20	77.7	99.3	39,788	97.2	2	this study
Durio zibethinus (durian)	[Helicteroideae] Durionoideae	715	30	22.7	98.5	35,832	98.8	3	ref.[3]
Ochroma pyramidale	Bombacoideae	1 790	42	55.1	99.6	44,752	92.7	5	ref.[18]
Bombax ceiba	Bombacoideae	762	48	16.6	99.4	39,399	97.7	5	ref.[19]
Gossypium raimondii (cotton)	Malvoideae	761	13	62.2	99.2	37,505	98.9	5	ref.[1]

PCG, protein-coding gene.

* neoautopolyploid genomes: only one (monoploid) genome was selected for subsequent analyses.

** monoploid genome size and chromosome number inferred from the pseudochromosome-level assemblies.

Supplementary Table 7. Summary of public genome resources used in this study.

Species	Genome type	Repository	Accession/Version/URL
Bombax ceiba	All	Figshare	https://doi.org/10.6084/m9.figshare.28408301.v2
Firmiana major	All	GWH	GWHDTXC00000000
Microcos paniculata	All	GWH	GWHDTXB00000000
Theobroma cacao	Nuclear	JGI	v1.1
Durio zibethinus	Nuclear	RefSeq	GCF_002303985.1
Ochroma pyramidale	Nuclear	Figshare	https://doi.org/10.6084/m9.figshare.22344934.v1
Gossypium raimondii	Nuclear	JGI	v2.1
Dipterocarpus gracilis	Nuclear	CNGB	https://db.cngb.org/search/assembly/CNA0034139
Aquilaria sinensis	Nuclear	GigaDB	https://dx.doi.org/10.5524/100702
Vitis vinifera	Nuclear	JGI	v2.1
Carica papaya	Nuclear	GWH	GWHBFSD00000000
Carica papaya	Mitochondrion	RefSeq	NC_012116.1
Gossypium raimondii	Mitochondrion	RefSeq	NC_029998.1
Theobroma cacao	Mitochondrion	RefSeq	NC_066894.1
Vitis vinifera	Mitochondrion	RefSeq	NC_012119.1
Carica papaya	Plastid	RefSeq	NC_010323.1
Gossypium raimondii	Plastid	RefSeq	NC_016668.1
Theobroma cacao	Plastid	RefSeq	NC_014676.2
Vitis vinifera	Plastid	RefSeq	NC_007957.1
Durio zibethinus	Raw reads	SRA	PRJNA400310
Ochroma pyramidale	Raw reads	GSA	PRJCA017171
Gossypium raimondii	RNA-seq reads	SRA	SRR8267559–SRR8267561

Note:

RefSeq https://www.ncbi.nlm.nih.gov/nuccore/
JGI https://phytozome.jgi.doe.gov/
GWH https://ngdc.cncb.ac.cn/gwh/
SRA https://www.ncbi.nlm.nih.gov/sra/
GSA https://ngdc.cncb.ac.cn/gsa/

Supplementary Table 8. Metrics for the genomes in the family.

Species	Relative ploidy (*p*)	Chromosome number (*x*)	Genome size (Mb)	LTR length (Mb)	TE length (Mb)	Number of syntenic genes	Chromosome dysploidy rate	Gene loss rate	Nucleotide substitution rate per million years	Plant height (m)	Subfamily	Species number of the subfamily	Genus number of the subfamily
Microcos paniculata	1	9	395.405	162.349	241.703	18,709	0.1818	0.0316	0.001278	12	Grewioideae	844	26
Theobroma cacao	1	10	346.165	127.246	186.715	19,320	0.0909	0.0000	0.000785	25	Byttnerioideae	774	27
Firmiana major	2	20	1417.596	1065.531	1180.909	25,472	0.0909	0.3408	0.001044	15	Sterculioideae	493	12
Diplodiscus trichospermus	2	20	545.541	142.048	299.937	25,994	0.0909	0.3273	0.001167	15	Brownlowioideae	94	9
Pterospermum kingtungense	2	19	1225.042	637.814	757.735	26,360	0.1364	0.3178	0.001306	12	Dombeyoideae	415	20
Craigia yunnanensis	4	41	1531.786	845.686	1073.258	46,568	0.0682	0.3974	0.000730	40	Tilioideae	56	3
Reevesia pubescens	2	20	1454.030	752.084	951.945	28,797	0.0909	0.2547	0.000942	16	Helicteroideae	148	12
Durio zibethinus	3	30	715.230	177.744	415.325	30,514	0.0909	0.4735	0.000917	40	Helicteroideae	148	12
Ochroma pyramidale	5	42	1794.081	1229.792	1390.519	34,562	0.2364	0.6422	0.000807	30	Bombacoideae	322	29
Bombax ceiba	5	48	762.389	353.541	488.206	31,697	0.1273	0.6719	0.001185	25	Bombacoideae	322	29
Gossypium raimondii	5	13	761.406	274.837	469.555	25,550	0.7636	0.7355	0.001743	7	Malvoideae	2342	114

Supplementary References

1. Paterson, A. H. et al. Repeated polyploidization of *Gossypium* genomes and the evolution of spinnable cotton fibres. *Nature*. **492**, 423–427 (2012).
2. Wang, X. et al. Comparative genomic de-convolution of the cotton genome revealed a decaploid ancestor and widespread chromosomal fractionation. *New Phytol.* **209**, 1252–1263 (2016).
3. Teh, B. T. et al. The draft genome of tropical fruit durian (*Durio zibethinus*). *Nat. Genet.* **49**, 1633–1641 (2017).
4. Wang, J. et al. Recursive paleohexaploidization shapes the durian genome. *Plant Physiol.* **179**, 209–219 (2019).
5. Conover, J. L. et al. A Malvaceae mystery: A mallow maelstrom of genome multiplications and maybe misleading methods? *J. Integr. Plant Biol.* **61**, 12–31 (2019).
6. Sun, P. et al. Subgenome-aware analyses reveal the genomic consequences of ancient allopolyploid hybridizations throughout the cotton family. *Proc. Natl. Acad. Sci. U. S. A.* **121**, e2313921121 (2024).
7. Bayer, C. et al. Support for an expanded family concept of Malvaceae within a recircumscribed order Malvales: a combined analysis of plastid *atp*B and *rbc*L DNA sequences. *Bot. J. Linnean Soc.* **129**, 267–303 (1999).
8. Alverson, W. S., Whitlock, B. A., Nyffeler, R., Bayer, C. & Baum, D. A. Phylogeny of the core Malvales: evidence from *ndh*F sequence data. *Am. J. Bot.* **86**, 1474–1486 (1999).
9. Nyffeler, R. & Baum, D. A. Phylogenetic relationships of the durians (Bombacaceae-Durioneae or /Malvaceae/Helicteroideae/Durioneae) based on chloroplast and nuclear ribosomal DNA sequences. *Plant Syst. Evol.* **224**, 55–82 (2000).
10. Baum, D. A. & Oginuma, K. A review of chromosome numbers in Bombacaceae with new counts for *Adansonia*. *Taxon*. **43**, 11–20 (1994).
11. Colli-Silva, M. et al. Taxonomy in the light of incongruence: An updated classification of Malvales and Malvaceae based on phylogenomic data. *Taxon*. **74**, 361–385 (2025).
12. Qian, H. & Jin, Y. An updated megaphylogeny of plants, a tool for generating plant phylogenies and an analysis of phylogenetic community structure. *J. Plant Ecol.* **9**, 233–239 (2016).
13. Rice, A. et al. The Chromosome Counts Database (CCDB) - a community resource of plant chromosome numbers. *New Phytol.* **206**, 19–26 (2015).
14. Marinho, R. C. et al. Do chromosome numbers reflect phylogeny? New counts for Bombacoideae and a review of Malvaceae s.l. *Am. J. Bot.* **101**, 1456–1465 (2014).
15. Liu, D., Tian, X., Shao, S., Ma, Y. & Zhang, R. Haplotype-resolved chromosomal-level genome assembly of Buzhaye (*Microcos paniculata*). *Sci. Data*. **10**, 901 (2023).

16. Motamayor, J. C. et al. The genome sequence of the most widely cultivated cacao type and its use to identify candidate genes regulating pod color. *Genome Biol.* **14**, r53 (2013).
17. Yang, J., Zhang, R., Ma, Y., Ma, Y. & Sun, W. Genome assembly of *Firmina major*, an endangered savanna tree species endemic to China. Preprint at https://doi.org/10.1101/2024.09.09.610897 (2024).
18. Sahu, S. K. et al. Chromosome-scale genomes of commercial timber trees (*Ochroma pyramidale*, *Mesua ferrea*, and *Tectona grandis*). *Sci. Data*. **10**, 512 (2023).
19. Yuan, G. et al. A telomere-to-telomere reference genome assembly of the red silk cotton tree (*Bombax ceiba*). *Sci. Data*. **12**, 1250 (2025).
